# Tissue- and Condition-Specific Biosynthesis of Ascorbic Acid in *Glycine max* L.: Insights from Genome-Wide Analyses of Pathway-Encoding Genes, Expression Profiling, and Mass Fraction Determination

**DOI:** 10.3390/ijms26104678

**Published:** 2025-05-14

**Authors:** Shahid Aziz, Thais Andrade Germano, Maria Adriele dos Santos de Sousa Do Nascimento, Clesivan Pereira dos Santos, Birgit Arnholdt-Schmitt, Maria Raquel Alcântara de Miranda, Mara Menezes de Assis Gomes, Luis Miguel Mazorra Morales, Ricardo Antônio Ayub, Jurandi Gonçalves de Oliveira, José Hélio Costa

**Affiliations:** 1 Department of Biochemistry, Institute of Chemistry, University of São Paulo, São Paulo 05508-000, SP, Brazil; 2 Functional Genomics and Bioinformatics, Department of Biochemistry and Molecular Biology, Federal University of Ceara, Fortaleza 60451-970, CE, Brazil; 3 Functional Cell Reprogramming and Organism Plasticity’ (FunCROP), Non-Institutional Competence Focus (NICFocus), Coordinated from Foros de Vale de Figueira, 7050-704 Alentejo, Portugal; 4 Centro de Ciências de Chapadinha, Universidade Federal do Maranhão, Boa Vista 65292-000, MA, Brazil; 5 Department of Biochemistry and Molecular Biology, Federal University of Ceara, Fortaleza 60451-970, CE, Brazil; 6 Laboratório de Melhoramento Genético Vegetal, Centro de Ciências e Tecnologias Agropecuárias, Universidade Estadual do Norte Fluminense Darcy Ribeiro (UENF), Campos dos Goytacazes, Rio de Janeiro 28013-602, RJ, Brazil; maramag@uenf.br (M.M.d.A.G.);; 7 Plant Physiology Institute, National University of La Plata, La Plata 1900, Argentina; 8 Laboratório de Biotecnologia Aplicada a Fruticultura, Departamento de Fitotecnia e Fitossanidade, Universidade Estadual de Ponta Grossa, Ponta Grossa 84010-330, PR, Brazil; rayub@uepg.br

**Keywords:** vitamin C, antioxidant, L-galactose, D-galacturonate, L-gulose, Myo-inositol, gene duplication

## Abstract

Ascorbic acid (AsA) is an essential plant metabolite that acts primarily as an antioxidant, regulates cell division and elongation, and enhances stress tolerance. Despite its crucial physiological role, the biosynthesis of AsA in *G. max*, a major crop of significant commercial importance, remains largely unexplored. This gap highlights the need for a thorough investigation of AsA biosynthesis pathways and their role in optimizing the nutritional value and stress tolerance of soybeans. This study identified 41 key genes linked to four AsA biosynthesis pathways in *G. max*, highlighting specific gene duplications compared to *Arabidopsis*. Their expression levels were assessed by analyzing a diverse set of RNA-Seq data from the NCBI database. Additionally, to cross-validate the expression levels of genes and the accumulation levels of AsA in the principal tissues, *G. max* plants were grown under controlled conditions following the protocols from selected RNA-seq experiments. Genes associated with the D-mannose/L-galactose pathway exhibited ubiquitous expression, and the expression patterns of genes from alternative pathways reflected their responsiveness to specific tissues or environmental conditions. Germination and leaf development were accompanied by strong expression of gene members from all pathways, whereas leaf aging was characterized by downregulation. Specific gene members, such as *GMP_2a* (D-mannose/L-galactose pathway), *GulLO_1f* (L-gulose pathway), and *MIOX_3a* (Myo-inositol pathway) were highly stress-responsive and linked to stress-resistant genotypes and cultivars. Consistent with gene expression analyses, the quantification of AsA revealed the highest mass fractions in young leaves and germinating seeds. However, AsA mass fractions were significantly reduced or unchanged under stress conditions, depending on the type of stress and the duration of exposure. Overall, this study validated the relevance of AsA biosynthesis pathways in soybeans, highlighting key genes that could be targeted to enhance stress tolerance and improve ascorbate production, thereby boosting the nutritional value of soybeans.

## 1. Introduction

Ascorbic acid (AsA), also known as vitamin C, is a vital eukaryotic antioxidant widely found in plants. Humans and other animal groups that have lost the terminal enzyme (L-gulono-1,4-lactone oxidase, or GulLO) catalyzing the last step of the AsA biosynthetic pathway cannot synthesize it, and for those, it has become a vitamin. All AsA auxotrophs, including humans, rely entirely on dietetic sources to meet their nutritional requirements of AsA [1]. For the majority of living species, AsA acts as a vital cofactor for numerous life-sustaining enzymes associated with a plethora of key processes, such as physiological and biochemical processes, including regulation of the cell cycle, photosynthetic pathways, and redox signaling [1,2]. In plants, AsA scavenges free radicals, thereby protecting DNA, proteins, and lipids from oxidative damage, which in turn increases yield through the improvement of cell health and resistance to stress [1,2]. The effectiveness of AsA in driving biochemical interactions is largely influenced by its concentration rather than its absolute content. Its higher concentration in specific tissues or cellular compartments improves molecular collision rates, allowing for efficient electron donation. However, diluted systemic distribution may limit bioavailability at critical sites, reducing therapeutic impact and highlighting that achieving sufficient concentration at action sites is crucial for efficacy [3,4].

The physiological significance of AsA in biotic/abiotic stress tolerance is well documented. In tomatoes, the exogenous application of AsA increased fruit ripening [5], while an AsA-deficient mutant of Arabidopsis showed earlier senescence than wild-type plants [6,7]. AsA applied together with drought stress-controlled ROS production and improved plant development by increasing the activity of antioxidant enzymes and stimulating drought stress tolerance in *Capsicum annuum* L. plants [8]. As a major component of the ascorbate–glutathione (AsA-GSH) cycle, AsA plays a substantial role in ROS detoxification [9]. Concerning conditions, AsA-GSH was differentially activated during stress, germination, and tissue development depending on the cellular compartment/genotype, revealing that cellular AsA level fluctuations can profoundly affect plant growth [10]. In common beans, water stress reduced photosynthetic pigments, carbonic anhydrase activity, antioxidant activities, growth, and seed yield, while, in contrast, AsA foliar spray enhanced all studied traits, with a positive correlation to increases in AsA dose [11]. Upon priming with AsA, aged oat seeds showed an effective alleviation of the detrimental effects of aging [12]. The above studies indicate that the harmful effects of abiotic or biotic stressors can be mitigated by improving the endogenous biosynthesis level of AsA or by its exogenous application.

**Figure 1:**
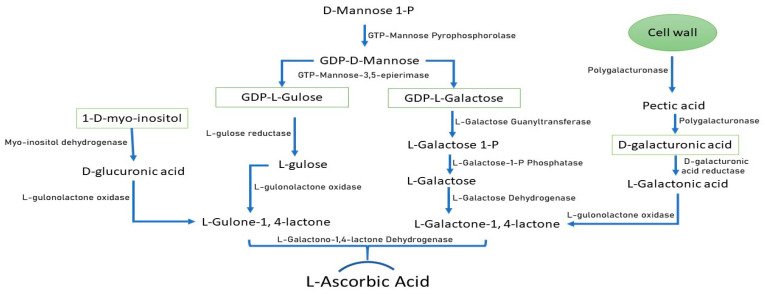
Overview of pathways leading to L-ascorbic acid (AsA) biosynthesis in plants. From left to right: Myo-inositol pathway, L-gulose pathway, L-galactose pathway, and D-galacturonic acid pathway.

In plants, AsA is synthesized through well-defined biochemical pathways [13,14] (Figure 1). The principal pathway for AsA biosynthesis is D-mannose/L-galactose (also called the Smirnoff–Wheeler pathway), which was proposed about 25 years ago and continues to be the major route to AsA synthesis in plant species [13]. This pathway is primarily regulated by eight key enzymes: Phosphomannose isomerase (PMI), Phosphomannomutase (PMM), GDP-mannose pyrophosphorylase (GMP), GDP-mannose 3′,5′-epimerase (GME), GDP-gulose phosphorylase (GGP), Gulose-1-phosphate phosphatase (GPP), Galactose dehydrogenase (GDH), and Galactonate dehydrogenase (GLDH) [15]. Furthermore, it has been observed that these genes exhibit light-responsive promoter motifs (such as the ATC, I-box, GT1 motif, etc.) [16]. Furthermore, it was shown that the GGP gene’s 5′ untranslated region contains a conserved cis-acting upstream open reading frame (5′-uORF), which promotes the post-transcriptional regulation of the gene [17].

D-galacturonate is another alternative pathway, derived from D-galacturonic acid, a component of pectin, a part of the plant’s primary cell wall [18]. Lower AsA content resulted from reduced pectin solubilization in the cell walls of transgenic strawberry fruit, which also had a decreased expression of an endogenous pectate lyase gene [18]. Transforming lettuce and tobacco with cDNA encoding L-gulono-1,4-lactone oxidase increased vitamin C leaf content by 4- to 7-fold [19]. Moreover, the expression of *GalUR* was shown to be associated with changes in the AsA level of strawberry fruit during the ripening process, as well as with differences in the AsA content of various *Fragaria species*, indicating that the D-galacturonic acid pathway predominantly operates in developing fruits [18]. The 2–3 times higher AsA levels in transgenic *Arabidopsis* were linked to the overexpression of *FaGalUR*, indicating that it is possible to engineer this gene to produce higher vitamin C levels in plants [18]. Feeding experiments on Arabidopsis, lettuce, potato, and tomato plants demonstrate enhanced AsA levels when pure L-gulose is supplied to plant tissues [20,21,22,23].

In 2003, the L-gulose pathway, also known as the L-gulose shunt, was proposed based on evidence of GDP-D-mannose conversion to GDP-L-gulose by GDP-D-mannose 3,5-epimerase (GME) and GDP-D-mannose [15]. In Arabidopsis lines overexpressing *GuILO*, drastically elevated biomass accumulation was seen in both aerial and root tissues compared to wild-type plants and empty vector controls growing under the same conditions [24]. These high-AsA lines also tolerated heat, cold, and salt better. Interestingly, the high-AsA lines did not exhibit the same severity of symptoms at the tested concentrations of protected pyrene (PYR). However, the wild-type plants experienced stunted growth of aerial tissue, fewer root hairs, and hindered leaf expansion [24]. There are multiple isoforms of *L-GulLO* in the Arabidopsis genome, with unclear activity [20]. AtGulLO5, a purified enzyme, exhibits similar properties to bacterial isozymes in terms of substrate specificity, subcellular localization, electron acceptor use, and activity, distinguishing it from plant and mammalian GulLOs. With the overexpression of L-GulLO genes of Arabidopsis in tobacco cells, higher AsA concentration was observed only when the cultures were supplied with L-gulono-1,4-lactone, revealing that this pathway is substrate-dependent [25].

The last alternative pathway that contributes to AsA biosynthesis is the Myo-inositol pathway [26]. Molecular and biochemical evidence of the Myo-inositol pathway as a possible biosynthetic route using myoinositol (MI) as the initial substrate was suggested [26]. The enzymatic activity of the MIOX gene was verified in bacterially produced recombinant proteins, and it was discovered on chromosome 4 (MIOX4) of the Arabidopsis ecotype Columbia. The Arabidopsis genome has four MIOX genes, which were given the names *AtMIOX1*, *AtMIOX2*, *AtMIOX4*, and *AtMIOX5* based on where on the chromosomes they are located [27]. During development, the four MIOX genes were differently regulated, with *MIOX2* being the most prevalent [27]. The involvement of *MIOX* in AsA synthesis and the possibility of exploiting this gene for the agronomic and nutritional development of crops were confirmed with a 2- to 3-fold increase in AsA, and the increased level was attributed to the overexpression of *AtMIOX4* [24,26]. Recently, five MIOX protein-coding genes and *MIOX* motifs were found in tomato (*S. lycopersicum*). When compared to controls, transgenic lines created by overexpressing the tomato *SlMIOX4* demonstrated a substantial increase in total AsA in the leaves and red fruits [28].

A recently published review article analyzing feeding studies on AsA biosynthesis via the three alternative pathways, namely D-galacturonate, L-gulose, and Myo-inositol, showed over a 100% increase in AsA content due to the overexpression of their key encoding genes [14]. Apart from the mentioned pathways, the regeneration of AsA occurs through the Foyer–Halliwell–Asada cycle and contributes to AsA level maintenance in the cell [29]. Moreover, metabolic pathways, including de novo biosynthesis, degradation, and recycling of AsA, collectively contribute to AsA biosynthesis in plants [30]. In addition, AsA concentration is also controlled by transcription factors (TFs), particularly under stress conditions, e.g., the leucine zipper domain (bZIP) transcription factor (TF) triggers AsA content in kiwifruits exposed to cold stress [16]. In Arabidopsis, the binding of ABA INSENSITIVE 4 (ABI4) TF to the promoter region of the VITAMIN C DEFECTIVE 2 (VTC2) gene inhibits AsA biosynthesis and, as a result, negatively regulates salt tolerance [31]. In summary, the relative contribution of AsA biosynthesis pathways and their specific role needs further elucidation; the overall AsA content varies depending on the plant species [32,33,34,35], cultivar, and genotype [36], tissue type [37], developmental and fruit ripening stage [38], and environmental conditions [24].

The current study identified genes associated with the four AsA biosynthesis pathways and analyzed their expression levels across tissues, developmental stages, and under stress conditions. Subsequently, we determined the AsA levels in the dried mass of selected tissues of *G. max* during development and stress conditions to validate the relevance of these pathways in soybeans. The identified genes and pathways might be targeted to increase AsA synthesis, with consequences for both nutritional plant quality and stress resilience in soybean crops, and they may have implications for agricultural practices in maximizing nutrient content under fluctuating environmental conditions.

## 2. Results

### 2.1. Identification and Phylogenetic Relationship of Genes Encoding AsA Biosynthesis Pathways in Soybean

**Figure 2:**
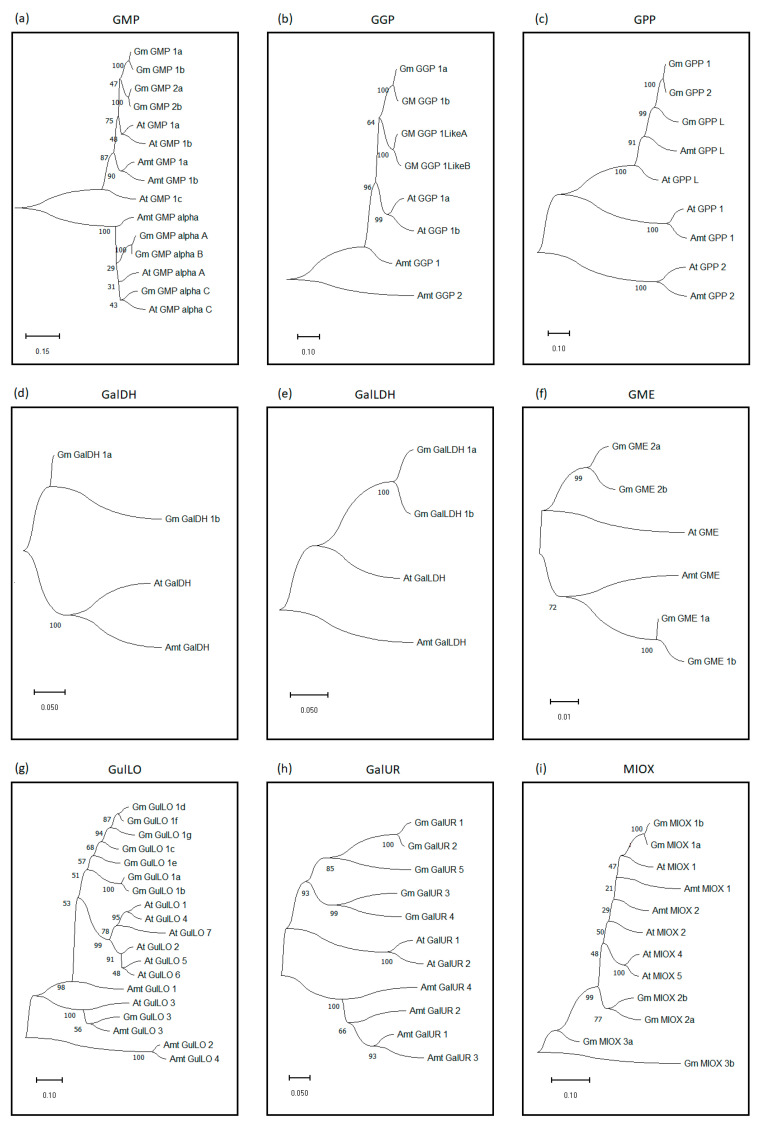
Phylogenetic relationship among the four AsA pathway protein sequences from *G. max* and *Arabidopsis thaliana* with *Amborella trichopoda* as outgroups (**a**–**i**). In the phylogenetic trees, branching corresponding to *G. max* is denoted with the prefix Gm and *Arabidopsis thaliana* is denoted with At, while the branches corresponding to *Amborella trichopodia* start with Amt. Horizontal distances are proportional to evolutionary distances according to the scale shown in the lower left corner at the bottom of each figure. The number near the branch indicates the bootstrap value.

Through BLASTn, using genes (cDNAs) that encode AsA enzymes in *Arabidopsis* as a query, 41 orthologous sequences were identified in the soybean genome available in the GenBank database (Supplementary Table S1). The cDNAs and the corresponding proteins for each gene were retrieved from RefSeq mRNA and/or protein databases and subsequently used in gene expression and phylogenetic analyses. Phylogenetic trees were constructed by aligning cDNA sequences from *G. max* (41), *A. thaliana* (26), and *Amborella* (23) (Figure 2, Supplementary Table S1), encoding the main proteins (GMP, GGP, GPP, GalDH, GalLDH, GME, GulLO, GalUR, and MIOX) involved in AsA synthesis. The protein sequences encoding the principal G. mannose/L. galactose pathway-encoding genes (GGP (GGP_1a, 1b, 1LikeA, and 1LikeB), GPP (GPP_L, 1 and 2), GalDH (GalDH_1a and 1b), GalLDH (GalLDH_1a and 1b), and GME (GME_1a, 1b, 2a, 2b)) were well clustered with their previously reported homologous genes in *A. thaliana* (Figure 2a–f). GulLO (GulLO_1a, 1b, 1c, 1d, 1e, 1f, 1g, and 3) from the L-Gulose pathway (Figure 2g), the D-Galacturonate pathway (GalUR (GalUR_1, 2, 3, 4 and 5)) (Figure 2h), and the MIOX_1a, 1b, 2a, 2b, 3a, and 3b genes were clustered (Figure 2i), whereas the *Amborella* sequences taken as an outgroup delineated the evolutionary relationship and highlight the distinct clustering of *G. max* and *A. thaliana* genes to each distinct pathway. Except for GPP, in which *Arabidopsis* and *G. max* comprise three gene members (Figure 2c), all other gene families showed a higher number of gene members in *G. max* than in *A. thaliana*.

### 2.2. Gene Expression Analyses Indicate That All Proposed AsA Biosynthesis Pathways Play an Active Regulatory Role in G. max Species

**Figure 3:**
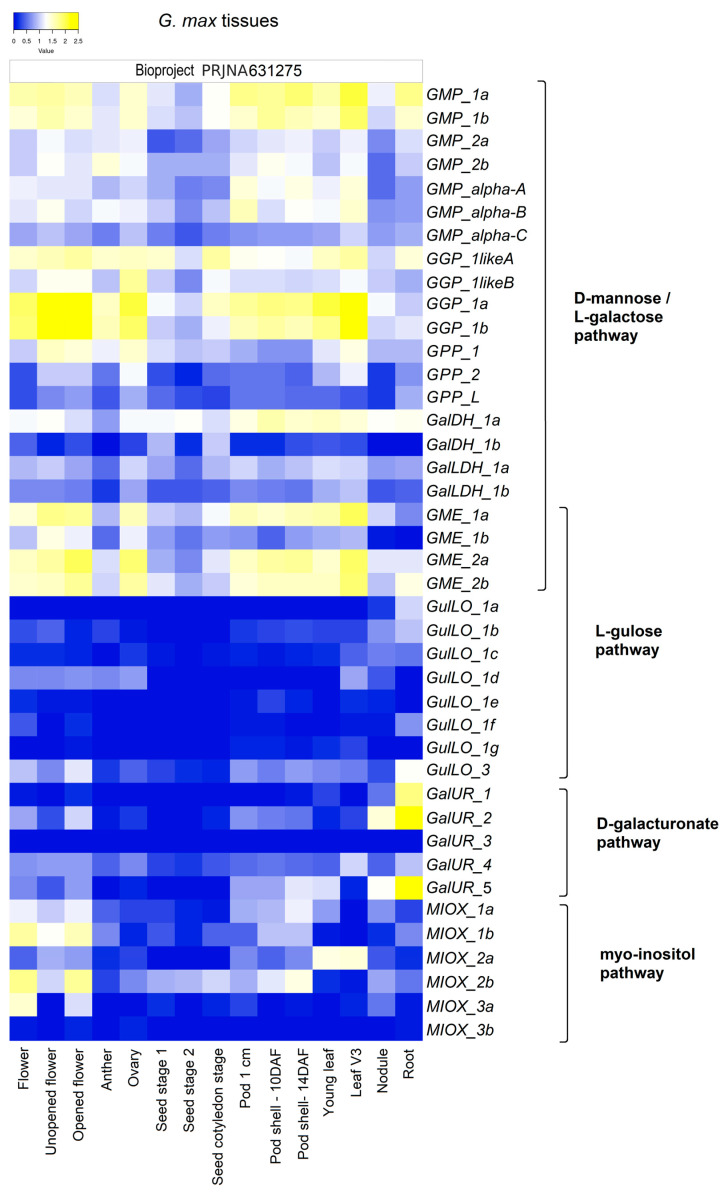
General view of the expression of the genes involved in AsA biosynthesis from the D-mannose/L-galactose, L-gulose, D-galacturonate, and Myo-inositol pathways in various tissues of *G. max*. The data are represented as log2 of the RPKM values (Log2RPKM) with a scale ranging from 0 (indicating low gene expression in blue) to 2.5 (indicating high gene expression in yellow). The RPKM values of the genes are shown in Supplementary Table S3.

The substantial expression level of pathway-specific genes across tissues (flower, anther, ovary, pod, leaf, root, root nodule, and seed) and in different physiological conditions (somatic embryogenesis, seed germination, and developmental stages) under ordinary environmental conditions evidences the active regulatory role of the four suggested AsA biosynthesis pathways in *G. max* (Figure 3). In Supplementary Table S3, the RPKM values reflect the expression levels of specific genes across tissues and conditions. Some gene members of the D-mannose/L-galactose pathway showed tissue-specific expression, such as *GGP_1a* and *1b* in the leaves and cotyledon and during somatic embryogenesis seed development; *GME_1a* was observed to be well expressed during seed germination, seed development, and in cotyledons; in addition, *GGP_1-likeA* was also well expressed in seed development. For the other pathways, a notable emphasis was observed on genes associated with the galacturonate pathway, particularly *GalUR_2* and 7, in roots and during seed germination. The expression analyses indicated that gene families associated with the D-mannose/L-galactose pathway were consistently detected, with at least one gene member exhibiting Log2(RPKM) values above 1.2 (on a scale ranging from 0 to 2.5) in each tissue evaluated. The genes with the highest expression level from the pathways included *GMP_1a* and *1b*, *GGP_1a* and *1b*, *GPP_1*, *GalDH_1a*, *GalLDH_1a*, and *GME_1a*, *2a*, and *2b*. Notably, these genes were most highly expressed in flowers, pods, and leaves (Figure 3). Regarding the gene families associated with the other three pathways, they were less expressed and showed specific gene members linked to particular tissues. In the L-gulose pathway, the majority of genes were minimally detected. Among them, *GulLO_3* was the most highly expressed gene and, together with *GulLO_1c*, *was* ubiquitously expressed. However, *GulLO_1a* was detected only in roots (including nodules); *GulLO_1d* was expressed in flowers (including anthers and ovaries), leaves (V3), and roots; and *GulLO_1b*, *1e*, *1f*, and 1*g* were not detected in seeds. For the galacturonate pathway, *GalUR_4* was a ubiquitously expressed gene despite its low mRNA levels, while *GalUR_1*, *2*, and 5 were highly expressed in the roots and not detected in the seeds, whereas *GalUR_3* was not detected in any of the tissues. Finally, from the Myo-inositol pathway, *MIOX_1a*, *1b*, and *2b* were ubiquitously detected in all tissues, with the highest mRNA levels in the flowers. *MIOX_2a* was predominant among the MIOX family members detected in leaves, whereas *MIOX_2b* was primarily expressed in seeds. *MIOX_3b* was either not detected or only barely detected in some tissues (Figure 3; Supplementary Table S3).

### 2.3. Expression Profile of Genes Linked to AsA Biosynthesis Pathways During Germination

**Figure 4:**
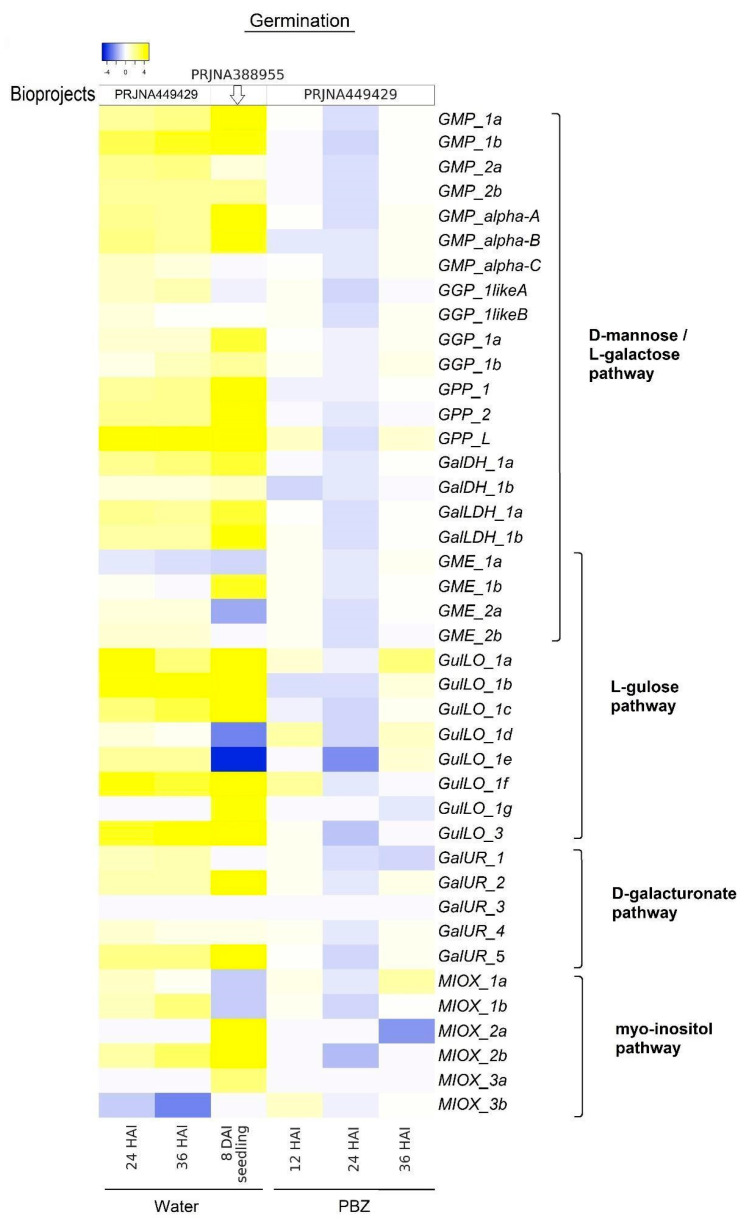
Heatmaps depicting the log2 fold value of genes linked to D-mannose/L-galactose, L-gulose, D-galacturonate, and Myo-inositol pathways in *G. max* during germination in water and in the presence of paclobutrazol (PBZ), with yellow indicating high gene expression levels and blue representing low gene expression levels. For both conditions, log2 fold change values are calculated considering the initial points (12 h of water) as a reference point, and statistical analyses are shown in Supplementary Table S4. The analyses are based on RNA-Seq data from bioprojects PRJNA449429 and PRJNA388955, each with three biological replicates. AsA gene expression was normalized using the RPKM method, as described in the Materials and Methods section.

In Figure 4, the heatmap (Log2FC values) shows the expression profile of genes involved in AsA biosynthesis during seed germination, both in water and in the presence of paclobutrazol (PBZ), a gibberellin (GA) biosynthesis inhibitor. In seeds, under water conditions (sown seeds), the majority of genes were upregulated at 24 and 36 h after imbibition (HAI) (compared to the initial time point of 12 HAI) (Figure 4, PRJNA449429), as well as in seedlings at 8 days after imbibition (DAI) (compared to the dry seeds) (Figure 4, PRJNA388955). Only a few genes were downregulated, such as *GME_1a* at 24 and 36 HAI (not significant) and at 8 DAI (significant), as well as *GGP_1likeA*, *MIOX_1a*, and *1b* in seedlings at 8 DAI (significant). On the other hand, treatment with paclobutrazol (PBZ) markedly reduced the expression of the majority of the genes from all the pathways at 24 HAI (Figure 4, PRJNA449429). In general, these differential expressions were significant at *p* < 0.05 (Supplementary Table S4).

### 2.4. Gene Expression Levels of AsA Biosynthetic Pathways Concerning Tissue Development

**Figure 5:**
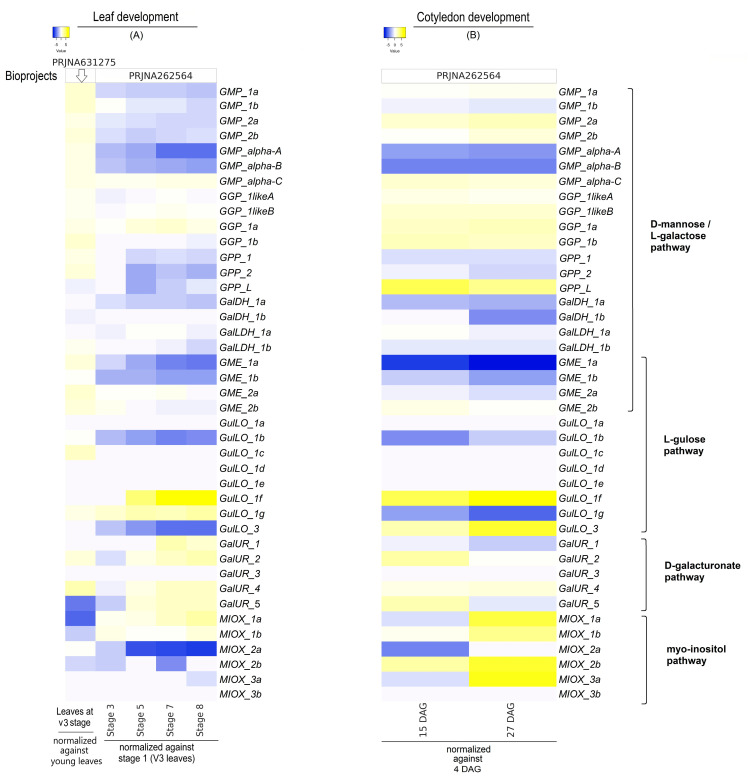
Heatmaps illustrating log2 fold change values highlight expression levels of AsA biosynthesis-associated genes from D-mannose/L-galactose, L-gulose, D-galacturonate, and Myo-inositol pathways in *G. max* throughout the developmental stages of leaves (**A**) and cotyledons (**B**). Blue and yellow shadings represent higher and lower expression levels, respectively. The data represent log2 fold change values relative to the first stage for each bioproject. For leaves, log2 fold change values were obtained concerning the V3 stage (bioproject PRJNA631275) and stage 1 (bioproject PRJNA262564). For cotyledons at 15 and 27 DAG, log2 fold change values were calculated concerning cotyledons at 4 DAG (bioproject PRJNA262564). Statistical analyses are shown in Supplementary Table S5.

**Figure 6:**
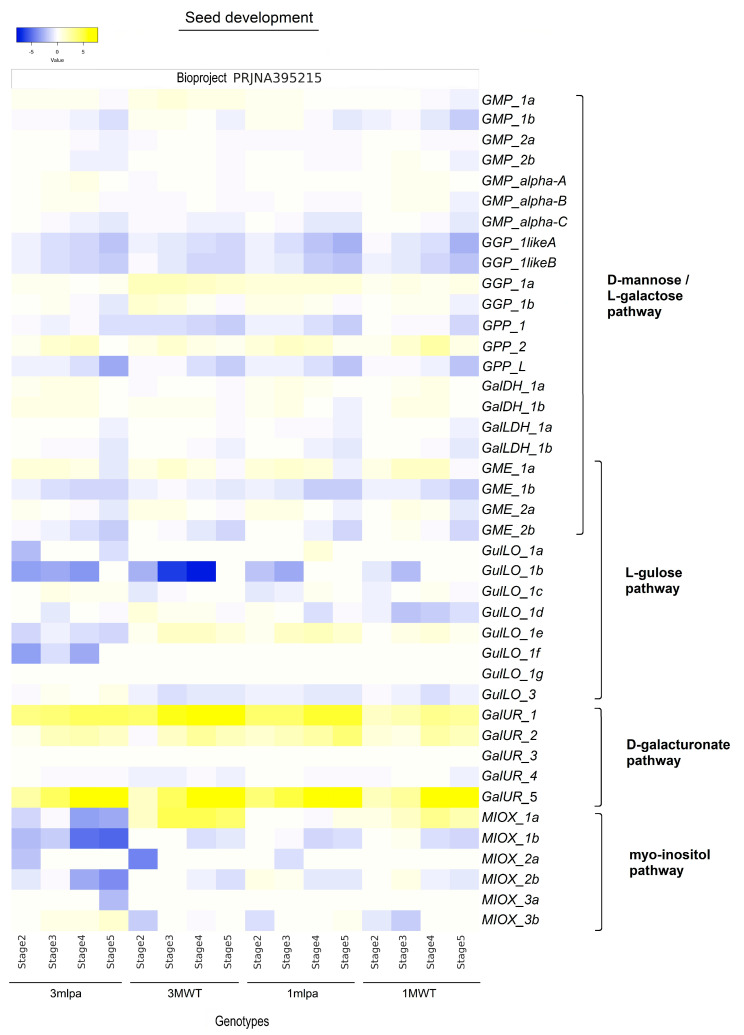
Heatmaps of log2 fold change values showing the differential expression of AsA biosynthesis genes from D-mannose/L-galactose, L-gulose, D-galacturonate, and Myo-inositol pathways in *G. max* during seed development. Yellow and blue colors represent gene expression levels that are high and low, respectively. Log2 fold change values were obtained at stage 1 for each genotype (bioproject PRJNA395215). Statistical analyses are shown in Supplementary Table S5.

The expression analyses of genes evaluated during the development of leaves, cotyledons, and seeds are shown in Figure 5 and Figure 6. In leaves (Figure 5A), several genes encompassing three AsA biosynthesis pathways were highly expressed at stage 1 (V3 stage) compared to young leaves (bioproject PRJNA631275), but the transcript levels of the majority of genes decreased during the leaf senescence period (stages 3 to 8 compared with stage 1). Among the D-mannose/L-galactose pathway-associated genes, all *GMP*, *GME* (except *GME_1b*), and *GGP* gene members, as well as *GPP_1* and *GPP_2*, showed increased transcript levels. In contrast, the genes linked to the two last steps of the pathway, *GalDH* (*1a* and *1b*) and *GalLDH* (*1a* and *1b*), did not show significant changes (Supplementary Table S5) at stage 1 (V3 stage). As for leaf senescence (stages 3 to 8 compared to stage 1) (bioproject PRJNA262564), only *GMP_alpha_C* and *GGP_1a* were found to be induced. Remarkably, while *GalDH* (*1a* and *1b*) expression significantly decreased, *GalLDH* (*1a* and *1b*) did not change during leaf senescence (Figure 5 and 6, Supplementary Table S5). For the gulose pathway, *GulLO_1c* mRNA levels increased only at stage 1 (V3) and, together with *GulLO_1d*, *1e*, and *1f*, reduced with leaf age and were minimally detected from leaf stages 3 to 8 (Supplementary Table S5). In addition, although *GulLO_3* was expressed across the time points, its expression was reduced during leaf senescence (stages 3 to 8). However, the *GulLO_1f* and 1*g* transcript levels increased across the leaf stages (5 to 8). For the galacturonate pathway, only *GalUR_4* mRNA levels increased at leaf stage 1 (V3 stage), and *GalUR_2*, *4*, and *5* expression increased in the leaf from stage 7 to 8. Last, for the Myo-inositol pathway, gene expression did not appear to be stimulated in leaves at the vegetative stage (compared to young leaves). *MIOX_2a* was the mainly expressed gene in leaves, but its expression decreased drastically from leaf stages 3 to 8. *MIOX_1a* and *1b* were expressed in young leaves, but their expression decreased at stage 1 and then increased at stage 8. Other genes from this pathway were barely detected. Overall, these expression levels were found to be significant at *p* < 0.05 (Supplementary Table S5).

Concerning cotyledons (Figure 5B), gene expression was evaluated at 15 and 27 DAG (days after germination) in comparison to the first time point (4_DAG). In parallel to leaf aging, some genes presented similar expression profiles in cotyledons, but some differences were observed. For instance, similar to leaves, mRNA levels of *GMP_alpha_A* and *alpha_B*, *GPP_1a* and *1b*, *GalDH_1a*, *GME_1a* and *1b*, *GulLO_1b* and *1c*, *and MIOX_2a* decreased, while *GMP_alpha_C*, *GulLO_1f*, and *GalUR_4* transcript levels increased. However, cotyledons showed a different profile compared to leaves, mainly in terms of the induced transcript levels of all the *GGP* gene members, including *MIOX_1a*, *1b*, *2b*, *3a*, and *GulLO_3*. In addition, *GulLO_1a*, *1d*, *1e*, *1g*, *and MIOX3b* were scarcely detected or not detected in cotyledons. Generally, these expression levels were significant at *p* < 0.05 (Supplementary Table S5).

For seed development (Figure 6; stages 2 to 5 in comparison to stage 1), comparative analyses of the four experimental genotypes (3mlpa, 3MWT, 1mlpa, and 1MWT) revealed some differences. However, a similar expression profile among genotypes was observed with increased transcript levels of *GalUR_1*, *2*, and *5*, *GGP1a*, *GPP_2*, and *GME_1a* (first stages) and with decreased mRNA levels of *GGP_1likeA* and *1likeB*, *GPP_L*, *GME_1b* and *2b*, and *MIOX_1b* and *2b*. Overall, these data were significant at *p* < 0.05 (Supplementary Table S5).

### 2.5. Expression Levels of Genes Involved in AsA Biosynthesis in G. max Roots and Leaves Under Abiotic Stresses

**Figure 7:**
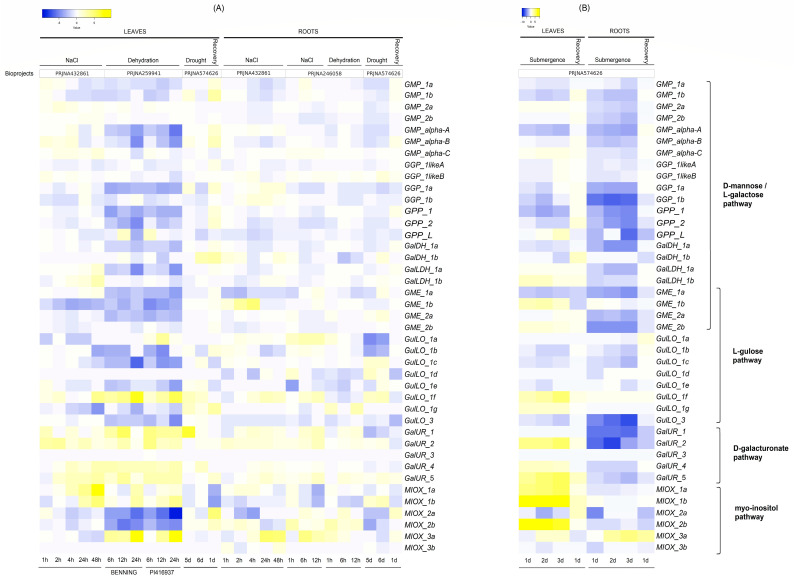
Log2 fold change values are represented in the heatmap to highlight the expression levels of genes from D-mannose/L-galactose, L-gulose, D-galacturonate, and Myo-inositol pathways in different tissues of *G. max* subjected to biotic stress such as (**A**) NaCl, dehydration, drought, and (**B**) submergence. In the heat map, blue indicates low gene expression levels, while yellow indicates high expression levels. The colors reflect log2 fold change values for each treatment relative to the initial time point (0 h) in each bioproject/tissue, and significant differences (at *p* < 0.05) between treatments (control and stress) are highlighted in Supplementary Table S6.

The gene expression analyses of AsA biosynthesis pathways in the roots and leaves of *G. max* were performed under various abiotic stresses such as NaCl, dehydration, drought (Figure 7A), and submergence (Figure 7B). In general, these stress conditions influenced the expression of various genes associated with AsA biosynthesis pathways, leading to intrinsic upregulation and downregulation among them.

For salinity, under NaCl stress (0 to 48 h), the leaves appeared to activate the D-mannose/L-galactose pathway (Figure 7A) rapidly. The GMP family genes of the pathway, including *GMP_1a*, *1b*, *alpha_B*, and one gene from the GGP family, *GGP_1likeB*, showed highly significant upregulation compared to the control during the initial 4 h of stress exposure. Interestingly, *GalLDH_1a* and *1b*, genes involved in the last step of this pathway, were upregulated later (24 to 48 h). In addition, *GME_1a* was also upregulated at 48 h; however, other GME gene members (*GME_1b*, *2a*, and *2b*) potentially involved in the D-mannose/L-galactose (or D-gulose) pathways were significantly reduced at 1 h after stress exposure and were reduced across the time points. Curiously, this early response also appeared with the galacturonate pathway genes *GalUR_1* and *2* at 2 h and *GalUR_4* and *5* upregulation at 4 h. Finally, AsA biosynthesis regulation in leaves under salinity could be associated with the strong upregulation of the Myo-inositol pathway-regulating genes, such as *MIOX_1a* (30-fold) and *1b* (41-fold), at 48 h. In the roots, D-mannose/L-galactose pathway stimulation seemed to be confined to *GME_1b* upregulation during the first 4 h and *GGP_1a* and *1b* later (Figure 7A). Interestingly, similar to the leaves, the D-galacturonate pathway responded to salt stress with high transcript accumulation from 1 h (*GalUR_1* and *2*) and 4 h (*GalUR_4* and *5*). In addition, the Myo-inositol pathway was also noted to be more active with *MIOX_1a* (first 4 h) and ultimately with *MIOX_3a* (after 4 h), a gene barely detected in *G. max* (Figure 5), but in salt-stressed roots, it was induced more than 30-fold. In general, these data were significant at *p* < 0.05 (Supplementary Table S6).

In the case of dehydration, the results of gene expression in leaf transcriptome data from two genotypes, Benning (drought-sensitive) and PI416937 (drought-tolerant), are shown in Figure 7A. Overall, dehydration reduced the mRNA levels of the D-mannose/L-galactose pathway, with no significant difference observed between genotypes. However, dehydration seemed to activate the L-gulose (mainly through *GulLO_1f* mRNA induction by 40 to 60-fold) and D-galacturonate (*GalUR_1*, *2*, *4*, and *5*) pathways early after 6 h, and finally the Myo-inositol pathway by high *MIOX* transcript (*MIOX_1a*, *1b*, and *3a*) induction at 24 h. In general, both genotypes appeared to respond similarly, but *GalUR* gene upregulation was more time-intensive in PI416937 (tolerant genotype). In roots, the D-mannose/L-galactose pathway also seemed less dehydration-responsive (or downregulated), while the L-gulose (by *GulLO_1a*, *1g*, and *GME_2a*) and Myo-inositol (*MIOX_2b*) pathways could be activated early at 1 h and the D-galacturonate pathway after 6 h (via *GalUR-1*, *2* and *5*) (Figure 7A). Mostly, these data were significant at *p* < 0.05 (Supplementary Table S6).

For drought, leaves and roots were analyzed after 5 and 6 days of stress exposure and 1 day of recovery (Figure 7A). In leaves, the gene expression of AsA biosynthesis pathways appeared mostly unaffected after a longer duration of stress exposure, while in roots, stress responses were more evident. Despite this unremarkable effect of drought in leaves, recovery largely stimulated genes from the D-mannose/L-galactose pathways. In roots, AsA biosynthesis genes from D-mannose/L-galactose were dominant and significantly downregulated at 5 and 6 days of drought; however, the L-gulose (by *GulLO_1c*, *1d*, *1f*, and *GME_2a*), D-galacturonate (by *GalUR_2* and *5*), and Myo-inositol (*MIOX_1b*) pathways could be activated, despite some downregulated genes in these pathways. Interestingly, recovery in roots generally reversed the downregulation or upregulation of AsA genes, indicating a vital drought response. Overall, these data were significant at *p* < 0.05 (Supplementary Table S6).

In submergence, leaves and roots exposed to stress for 1 to 3 days and recovery (1 day) revealed peculiar up-and downregulated genes (Figure 7B). In leaves, although most of the genes from the D-mannose/L-galactose pathway were downregulated, certain genes, such as *GMP_2a* and *alpha_C*, *GPP_L*, *GME_1b* and *2b*, and *GalLDH* (*1a* and *1b*), could still contribute to maintaining the activation of this pathway. However, the other pathways appeared to be more activated with the upregulation of key genes, such as *GulLO_1f* and *1g* for L-gulose, *GalUR_1*, *2*, *4*, and *5* for D-galacturonate, and *MIOX_1a*, *1b*, and *2b* for the Myo-inositol pathway. In roots, the large majority of genes were severely downregulated compared to leaves. Only *GulLO_1f* and *MIOX_3a* were upregulated. Curiously, recovery in leaves and roots typically reversed the down- and upregulation of AsA genes. The differential expression of most of the genes was found to be significant at *p* < 0.05 (Supplementary Table S6).

### 2.6. Expression Level of Genes Associated with AsA Biosynthesis in Leaves and Roots Under Biotic Stresses

**Figure 8:**
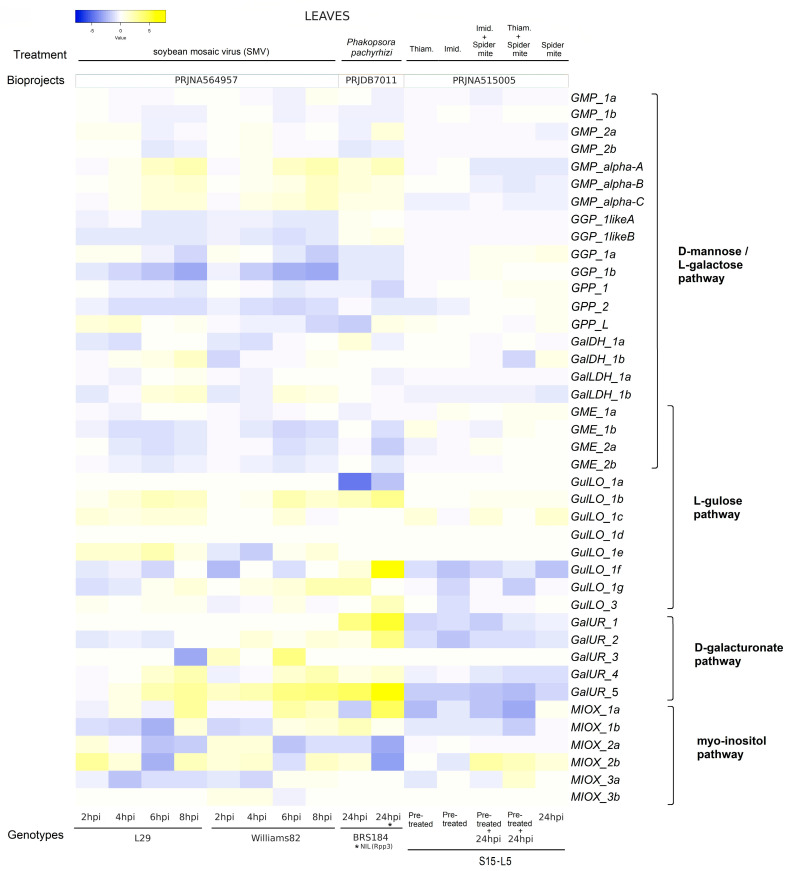
Heatmaps of log2 fold change values showing the differential expression of AsA biosynthesis genes from D-mannose/L-galactose, L-gulose, D-galacturonate, and Myo-inositol pathways in leaves of *G. max* under biotic stresses. Yellow and blue colors represent gene expression levels that are high and low, respectively. Leaves under SMV [in resistant (L29) and susceptible (Williams82) soybean cultivars]; *Phakopsora pachyrhizi* [in two genotypes, BRS184 (susceptible) and NIL (Rpp3) (resistant)]; and spider mite (+/− insecticides: thiamethoxam and imidacloprid) and insecticides. For leaves under SMV, the data represent values of log2 fold change with the first time point (0 h), while for leaves under *P. pachyrhizi* and spider mites/insecticides, the log2 fold change was calculated about the mock. Statistical analyses are shown in Supplementary Table S7.

**Figure 9:**
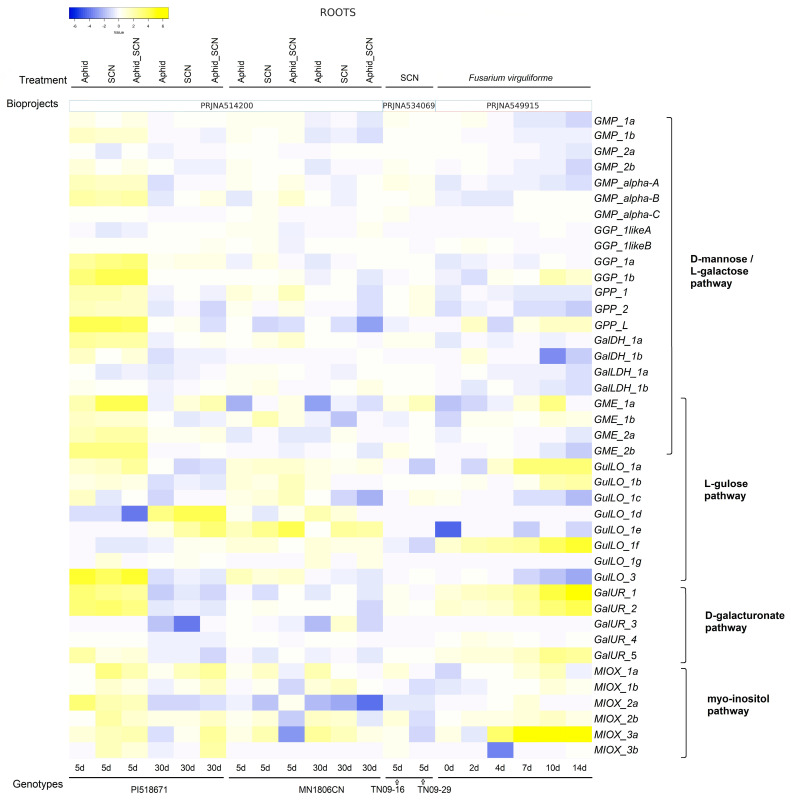
Heatmaps of log2 fold change values showing the expression level of genes from D-mannose/L-galactose, L-gulose, D-galacturonate, and Myo-inositol pathways in the root of *G. max* subjected to biotic stresses. Yellow and blue colors represent gene expression levels that are high and low, respectively. Roots infected with aphid and SCN (together and apart) in two genotypes: PI518671 (SCN- and SBA-susceptible) and MN1806CN (SCN-resistant and SBA-susceptible) and *F. virguliforme*. The log2 fold change was obtained concerning the mock with the same time point of the treatment. Statistical analyses are shown in Supplementary Table S7.

Figure 8 illustrates the expression levels of genes linked to AsA biosynthesis in the leaves and roots of *G. max* during exposure to different biotic stresses. In leaves, gene expression was evaluated in response to soybean mosaic virus (SMV), *Phakopsora pachyrhizi*, and spider mite (Figure 8; Supplementary Table S7), while the roots were analyzed in response to soybean cyst nematode (SCN), aphids (SBA), and *Fusarium virguliforme* (Figure 9; Supplementary Table S7).

Leaves of cultivars with contrasting responses to SMV, L29 (resistant), and Williams 82 (susceptible) revealed a similar up- and downregulation of genes, with peculiar differences in the D-mannose/L-galactose pathway (Figure 8).

The transcript levels of GMP_alpha_A, B, and C, as well as *GalLDH_1b*, increase mainly at 6 and 8 hpi, which would activate the D-mannose/L-galactose pathway in the leaves of both cultivars; however, specific mRNA accumulation of *GMP_2a*, *GalDH_1a*, and *1b*, and *GalLDH_1a* in the L29 cultivar (resistant) could support higher plasticity in AsA biosynthesis through this pathway than in the Williams 82 (susceptible) cultivar. Similarly, the increased transcript levels of genes associated with the L-gulose (*GulLO_1b*), D-galacturonate (*GalUR_4* and *5*), and Myo-inositol (*MIOX_2a* and *2b*) pathways also indicate that these pathways could be activated in both cultivars during SMV infection. *Phakopsora pachyrhizi* infection was also investigated in two contrasting genotypes, BRS184 (susceptible) and NIL (Rpp3) (resistant), and revealed some similarities with the gene expression pattern in response to SMV. The resistant genotype showed a higher number of upregulated genes (*GMP_alpha_A*, *B*, and *C*) of the D-mannose/L-galactose pathway than the susceptible genotype (*GMP_alpha_A*). Among the genes of other pathways, although both genotypes showed similar mRNA levels of *GalUR_1*, *2*, and *5* from the D-galacturonate pathway, they differed in the other two pathways, with increased mRNA levels of *GulLO_1f* and *3* from the L-gulose pathway in the case of the resistant genotype and *MIOX_1b* and *2b* from the Myo-inositol pathway in the susceptible genotype. Finally, the last experiment on leaves treated with spider mite (+/− insecticides: thiamethoxam and imidacloprid) and only insecticides revealed a very different expression profile compared to SMV and *Phakopsora pachyrhizi*, with a prevailing downregulation of genes from the D-galacturonate (*GalUR_2* and *5*) and Myo-inositol (*MIOX_1a*, *1b*) pathways in response to insecticides and upregulated genes linked to the D-mannose/L-galactose (*GGP_1a* and *GME_1a*), L-gulose (*GulLO_1c*), and Myo-inositol (*MIOX_2b*) pathways in response to spider mite (Figure 9). In general, these differential expressions were significant at *p* < 0.05 (Supplementary Table S7).

In roots, gene expression analysis in response to SCN (PRJNA534069) and aphids (SBA) in two genotypes [PI518671 (SCN- and SBA-susceptible, PRNA5142200) and MN1806CN (SCN-resistant and SBA-susceptible)] revealed a remarkable and extensive increase, principally in the transcription of the D-mannose/L-galactose pathway in the SCN-susceptible genotype at 5 days after exposure to the stress (Figure 9). This genotype responded similarly to all infections (aphid, SCN, and SCN–aphid) by upregulating a large majority of genes from the D-mannose/L-galactose pathway and some key genes from the other three alternative pathways (*GulLO_3*, *GalUR_1*, and *2*, *MIOX_1a*, *2a*, and *3a*). In SCN-resistant genotypes, this general response appeared substantially attenuated at 5 days after the exposure to stress; however, interestingly, the transcript levels of *GulLO_1f* and *MIOX_3a* were already elevated in control conditions and further increased (*MIOX_3a* was 10 to 60-fold higher) in stress conditions (except the SCNA_phid treatment) within the susceptible genotype. At 30 days, the genes generally showed attenuated upregulation and returned to baseline levels similar to the control or were slightly downregulated, particularly in the susceptible genotype (Supplementary Table S7). In the second experiment, roots infected with *Fusarium virguliforme* (0, 2, 4, 7, 10, and 14 days, PRJNA549915) revealed that genes of the D-mannose/L-galactose pathway appeared more downregulated despite an increase in some transcripts (*GGP_1b*, *GPP_L*, and *GME_1a*), mainly from 10 days of the stress exposure. However, the most imperative response occurred with genes from the L-gulose (*GulLO_1a*, *1b*, and *1f*), D-galacturonate (*GaIUR_1*, *GaIUR_2*, and *GaIUR-5*) and Myo-inositol (*MIOX_3a*) pathways, in which the transcript levels increased by at least 10-fold compared to the control conditions from 4 days of the stress exposure (Figure 9). The statistical significance analysis of these data is detailed in Supplementary Table S7.

### 2.7. AsA Mass Fraction Determination in Selected Soybean Tissues Under Development and Stress Conditions

**Figure 10:**
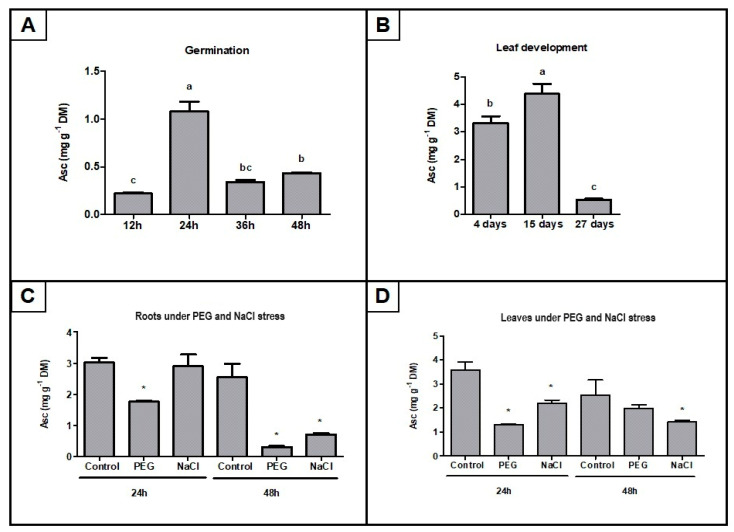
Mass fraction of AsA in *G. max* organs across developmental stages and under abiotic stress conditions: (**A**) seed germination; (**B**) leaf development; (**C**) roots under PEG and salinity stress; (**D**) leaves under PEG and salinity stress. Bar charts represent mean ± standard deviation (*n* = 3). Different letters indicate significant differences (*p* < 0.05) between germination/development time points determined by the Bonferroni test. Asterisks indicate significant differences (*p* < 0.05) between the control and stress conditions determined by the t-test for each mass fraction. PEG stands for polyethylene glycol, Asc stands for ascorbate, and DM stands for dry matter.

To validate the significance of the gene expression data of AsA biosynthesis pathways in soybeans, the AsA mass fraction in dry matter was determined in leaves and seeds across developmental stages and leaves and roots under abiotic stress conditions (Figure 10). During seed germination, AsA mass fractions increased from the initial stages at 12 h to 24 h (Figure 10A) and declined afterward. The comparison of developing leaves in Figure 10B revealed a significantly higher relative accumulation of AsA in leaves at 15 days than in leaves at 4 days. Both roots and leaves under PEG and NaCl presented a decline in AsA mass fractions compared to the control; however, this response varied regarding the time of exposure and stress condition (Figure 10C,D). In leaves, PEG decreased AsA levels only at 24 h, while in roots, NaCl reduced AsA levels only at 48 h (Figure 10C,D). The higher decline in AsA mass fraction under both stress conditions occurred in roots at 48 h (Figure 10C,D).

## 3. Discussion

Ascorbic acid (AsA, ascorbate, vitamin C) is well known for its indispensable roles in plant and human metabolism, primarily acting as an antioxidant, free radical scavenger, and cofactor for numerous enzymes [2]. However, humans cannot synthesize AsA. Thus, obtaining it from plant-based foods or pharmaceutical supplements is essential. In plants, AsA protects chloroplasts against ROS, is involved in ethylene synthesis, and acts as a cofactor of dioxygenases, which are associated with abscisic acid (AsA) and gibberellin synthesis besides auxin catabolism [1,2,39]. Consequently, this direct relationship between AsA and phytohormones denotes the critical involvement of AsA during all phases of plant growth and development, in addition to abiotic and biotic plant responses. Therefore, studies on AsA biosynthesis regulation in plants can benefit crop improvement that promotes healthy plants with high yield stability, as well as the development of plants with higher levels of AsA.

**Figure 11:**
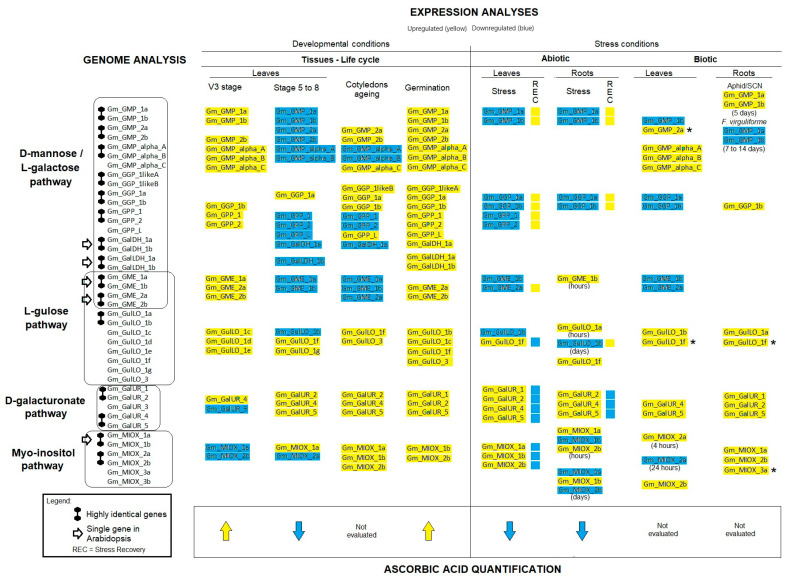
Schematic representation of the main findings of this study: on the left, highly identical gene pairs and duplicated genes of *G. max* compared with those of *Arabidopsis thaliana* were identified in genomic analysis. The up- and downregulated genes involved in the four ascorbic biosynthesis pathways in *G. max* during developmental and stress conditions are shaded in yellow and blue, respectively. Ascorbic acid quantification consistently validated the gene expression analyses. Genes marked with asterisks are highly stress-induced in stress-resistant genotypes.

This study aimed to gain insight into the evolution and regulation of AsA biosynthesis genes under both development stages and stress conditions to identify genes that could be potentially advantageous for biotechnological application, followed by AsA-level quantification to cross-validate the expression analyses of the identified genes. The key findings of this study are illustrated in Figure 11. In addition, this study advances the concept of the connection between AsA biosynthesis and ROS scavenger activity of the AsA-GSH cycle [10].

The genome-wide analyses identified 41 genes encoding the key enzymes (GMP, GGP, GPP, GalDH, GalLDH, GME, GulLO, GalUR, and MIOX) potentially involved in the four AsA biosynthesis pathways in *G. max* against the total of 26 in *Arabidopsis* (Figure 2, Supplementary Table S1). This higher gene number in *G. max* seemed to be due to duplication events (Figure 2). Among these genes, some paralogous pairs revealed high nucleotide sequence identities between them, such as *Gm_GMP_1a* and *1b* (94.93%), *Gm_GMP_alpha-A* and *B* (95.84%), *Gm_GMP_2a and 2b* (96.99%), *GME_1a* and *1b* (91.38%), *GME_2a* and *2b* (93.15%), *Gm_GalLDH_1a* and *1b* (93.31%), and *Gm_MIOX_1a* and *1b* (93.58%), indicating genes derived from recent duplication events as depicted in the phylogenetic trees (Figure 2). Studies reported duplicated genes in *G. max* for other proteins such as EF1α [40], PTOX [41], and the AsA-GSH cycle [10]. In effect, duplicated genes are widespread in the soybean genome, covering around 75% of genes [42]. Concerning gene expression, *GMP* and *GalLDH* (D-mannose/L-galactose pathway) duplicated gene pairs revealed similar expression profiles regarding all tissues and development stages under control conditions; however, differences were observed under stress conditions. Different expression profiles for *GME* and *MIOX* also prevailed regarding various tissues and stress conditions (Figure 3, Figure 4, Figure 5, Figure 6, Figure 7, Figure 8 and Figure 9; Supplementary Tables S3–S7). Thus, these data indicate that duplicated genes in *G. max* gained functional diversity (neofunctionalization) or divided original function (subfunctionalization) between duplicated genes, mainly induced by stress conditions. In addition, other genes such as *GalUR_3* and *MIOX_3b* were not detected (or scarcely detected) in any tissue/condition, suggesting a possible loss of function (*pseudogenization*—nonfunctional genes) [43,44].

Regarding the general expression profiles of all genes from *G. max*, it was evident that genes from the D-mannose/L-galactose pathway were ubiquitously expressed with higher mRNA levels (Figure 3; Supplementary Table S3), showing fluctuations of up/downregulation among gene members during development and stress conditions (Figure 4, Figure 5, Figure 6, Figure 7, Figure 8, Figure 9 and Figure 10; Supplementary Tables S4–S7). In contrast, genes from the L-gulose, D-galacturonate, and Myo-inositol pathways were usually expressed at low levels or not expressed under control conditions, despite some ubiquitously expressed genes (*GulLO_1c and 3*, *GalUR_4*, *and MIOX_1a*, *1b* and *2b*). Specific gene members were strongly stimulated or induced under different stress conditions (Figure 5, Figure 6, Figure 7, Figure 8 and Figure 9; Supplementary Tables S3–S7). These data highlight that the D-mannose/L-galactose pathway is the main constitutive AsA biosynthetic pathway in *G. max*. This outcome underlines studies in *Arabidopsis* with mutants that had highlighted D-mannose/L-galactose as the main pathway for AsA supply in this model species [45,46,47,48]. Other genetic and biochemical studies also suggested D-mannose/L-galactose as the major AsA biosynthetic pathway in streptophytes [49]. Despite the main role of the D-mannose/L-galactose pathway, our findings point to a mutual regulation with other AsA biosynthetic pathways regarding developmental stages and stress conditions. During germination, increased AsA levels (Figure 10) reflect a significant increase in the mRNA levels of most of the genes from all the AsA biosynthesis pathways (Figure 4; Supplementary Table S4), suggesting that all the proposed pathways cooperatively contribute to the total AsA level.

It has been observed that a high upregulation of genes encoding proteins of the AsA-GSH cycle from different cellular compartments reinforces the critical role of AsA during *G. max* seed germination [10]. Germination depends on ROS to break seed dormancy and promote germination [50]. However, this process needs to be fine-tuned to avoid excessive ROS. The presented data point to an interaction between AsA biosynthesis and the AsA-GSH cycle to maintain redox homeostasis during germination in *G. max*. AsA’s beneficial effect on germination has been evidenced in other leguminous plants such as alfalfa [51] and cowpea [52], where the exogenous application of AsA increased the germination rate and stress tolerance. Advancing AsA biosynthesis regulation in *G. max*, we observed the adverse effect of PBZ (a gibberellin inhibitor) on AsA biosynthesis genes (Figure 4; Supplementary Table S4); a similar response was previously detected for AsA-GSH cycle genes [10]. Gibberellin (GA) and abscisic acid (ABA) are antagonistically acting phytohormones during seed germination, with ABA maintaining seed dormancy and GA promoting germination, and a homeostatic GA/ABA balance needs to be reached to boost germination [53,54]. Therefore, GA inhibition could cause a GA/ABA ratio imbalance favoring the ABA effect and limiting germination by a mechanism involving AsA biosynthesis decrease.

Consistent with the high mass fraction of AsA found in young leaves (Figure 10B), gene expression of the D-mannose/L-galactose route indicated a remarkable activation, followed by a moderate enhancement of the L-gulose and D-galacturonate pathways in leaves at the vegetative stage (Figure 5A; Supplementary Table S5). Mutant analyses showed that D-mannose/L-galactose is dominant among the AsA biosynthetic pathways in photosynthetic tissues [55]. In addition, the intense increase in mRNA levels of organellar AsA-GSH cycle genes in the same experiments supports the biosynthetic requirement of AsA [10]. Indeed, the essential role of AsA in the vegetative phase has been observed in several plant species, and exogenous AsA application substantially improved biochemical and physiological parameters [56,57]. Interestingly, the aging of the leaves changed the expression profile of the AsA biosynthesis gene, with a prevailing downregulation of D-mannose/L-galactose genes and upregulation of specific genes from the L-gulose (*GulLO_1f* and *1g*), D-galacturonate (*GalUR_2*, *4* and *5*), and Myo-inositol (*MIOX_1a* and *1b*) pathways (Figure 5A; Supplementary Table S5). These findings support the decreased AsA mass fractions observed across the developmental stages of leaves (Figure 10).

Cotyledon senescence maintained this tendency but with the upregulation of specific genes such as *GMP_1a*, *2a*, *2b*, and all *GGP* of the D-mannose/L-galactose pathways (Figure 5B; Supplementary Table S5). The upregulation of Myo-inositol genes in aged tissues presented a remarkable difference compared to leaves at the vegetative stage (Figure 3). A decrease in Myo-inositol biosynthesis in apple leaves was associated with extensive programmed cell death [58]. Hence, the activation of the Myo-inositol AsA biosynthesis pathway in aging *G. max* tissues could contribute to controlling Myo-inositol and may have a minor contribution to increasing AsA synthesis during senescence.

In stress conditions, a similar tendency was observed during senescence, stimulating/inducing the gene expression of the L-gulose, D-galacturonate, and Myo-inositol pathways. This was remarkable, with substantial increases in mRNA levels or a strong induction of specific genes. However, stress responses varied based on the type of stress, duration of exposure, and plant tissue types. Depending on stress, specific D-mannose/L-galactose genes were also upregulated or occurred with significant downregulation but always maintained basal mRNA levels (Figure 7, Figure 8 and Figure 9; Supplementary Tables S6 and S7). This general expression pattern supports the conclusion that the D-mannose/L-galactose pathway could function as the major and constitutive AsA biosynthesis pathway. In contrast, the other three AsA pathways could be complementary and indispensable pathways in response to development and environmental fluctuations.

Given the vital importance of AsA for plant metabolism, it is essential to take into account that stresses may reduce the AsA mass fraction in plant tissues, affecting the efficiency of the D-mannose/L-galactose pathway and/or the increased ROS scavenging activity using AsA [59]. In this regard, the downregulation of key genes from the D-mannose/L-galactose pathway was consistent with the reduced AsA mass fractions in leaves and roots under PEG and NaCl conditions (Figure 10 and Figure 11). In addition, the upregulation of AsA-GSH cycle genes under the same experiments from the current study supports the idea of increased AsA consumption for ROS scavenging activities [10].

In former studies, our group observed an extensive upregulation of specific genes encoding APX from different cell compartments in response to the same abiotic and biotic stresses in *G. max* that were investigated here [10]. In this scenario, it is possible to assume that alternative AsA biosynthesis pathways could be activated to attenuate AsA decrease or maintain or increase AsA levels in plant cells. Thus, cultivars/genotypes that manage this cellular reprogramming well, increasing AsA levels, would adapt with enhanced stress tolerance.

In the abiotic and biotic stresses evaluated in this study, three gene members revealed the potential to be selected as candidates for plant breeding: *GMP_2a* (D-mannose/L-galactose pathway), *GulLO_1f* (L-gulose pathway), and *MIOX_3a* (Myo-inositol pathway). The *GMP_2a* gene was ubiquitously expressed; however, it was upregulated in cotyledon senescence, abiotic stresses, and biotic stresses (in leaves) (Figure 7, Figure 8 and Figure 9; Supplementary Tables S6 and S7). In biotic stresses, *GMP_2a* revealed an upregulation associated with resistant genotypes (Figure 8 and Figure 9; Supplementary Table S7). Concerning *GulLO_1f* and *MIOX_3a*, generally, they were scarcely detected in control conditions, but they were strongly induced under different stress conditions (Supplementary Tables S6 and S7). These genes also revealed higher transcript levels linked to resistant genotypes in roots (Supplementary Tables S6 and S7). Numerous engineering strategies using AsA biosynthetic genes have been proposed, aiming to increase AsA level/plant stress tolerance [60]. Among these, ten used *GMP* genes; however, only one study was associated with tolerance (tested with the application of methyl viologen as a stress inducer) [61]. In rice and *Arabidopsis*, MIOX gene overexpression resulted in enhanced tolerance to drought, salt, cold, and heat stresses [24,61,62]. For GulLO genes, eight strategies using *Arabidopsis* or rat genes originated different species resistant to various abiotic stresses [21,24,63]. Despite the success, researchers still focus on model species (rice and *Arabidopsis* or rats) as gene sources. This study explored *G. max* genes as a promising alternative. Duplication events have shaped these *G. max* genes’ evolutionary history and may have introduced enhanced resilience and adaptability to stress tolerance [64,65].

## 4. Material and Methods

### 4.1. Plant Materials and Growth Conditions

Soybean BRS Pala seeds were obtained from the germplasm bank of the Department of Phytotechnics at the Federal University of Ceara, Brazil. The seeds were disinfected with 70% (*v*/*v*) ethanol for 1 min, followed by immersion in a 1% (*v*/*v*) sodium hypochlorite solution for 3 min, and were then rinsed three times with sterile distilled water. After rinsing, the seeds (*n* = 140) were inoculated in sterile 50 mL test tubes containing 15 mL of MS medium without sucrose [66]. For seed germination, the tubes were stored in a germination chamber under a controlled temperature (25 ± 2 °C) and a 12/12 h photoperiod (light/dark) under a white fluorescent light of 10–15 μmol.m^2^·s^−1^ intensity provided by a fluorescent light-emitting diode (LED) [67,68]. The in vitro cultivation in MS medium was conducted for 27 days. Germinating seeds were collected at 12, 24, 36, and 48 h, whereas leaves were collected at three distinct time points: 4, 15, and 27 days. Samples’ fresh weights were taken and later placed under 105 °C for 24 h to determine the dry mass.

### 4.2. Abiotic Stress Induction

To investigate the impact of abiotic stress on AsA biosynthesis, 27-day-old seedlings were transferred to hydroponics using a 50% Hoagland nutrient solution [69], where they remained for 72 h in a B.O.D. chamber under the same photoperiod and temperature conditions as under in vitro cultivation acclimatization. Subsequently, the seedlings were subjected to stress treatments. The acclimatized seedlings were then transferred again to hydroponics, now containing 100% Hoagland nutrient solution, in 3 L pots and divided into three groups. Then, each condition (control, saline stress, and water stress) was represented by three different pots with 10 seedlings each, where a pot represents one biological replicate. At each time point (24 and 48 h), three seedlings were collected from each biological replicate (one pot); in total, there were nine seedlings per condition (control, salt, and drought stress). The control group received only 100% Hoagland solution, the saline stress group received 100% Hoagland solution with 100 mM NaCl, and the water stress group received 100% Hoagland solution with 200 g·L^−1^ PEG 6000 (polyethylene glycol). Leaves and roots were collected at 24 and 48 h after initial treatment. After collection, the samples (three biological replicates and three technical replicates) were frozen in liquid nitrogen and stored at −80 °C until the preparation of extracts for ascorbate quantification.

### 4.3. Quantification of AsA

For the quantification of AsA, 0.1 g of the plant material was homogenized with 2.5 mL of 5% TCA solution and centrifuged at 15,000× *g* for 12 min at 4 °C [70]. Then, the aliquot of supernatant (50 µL) was homogenized with 25 µL of 100 mM potassium phosphate buffer (pH 7.7) and 175 µL of the solution containing 10% TCA, 8.8% phosphoric acid, 0.8% 2,2-bipyridyl, and 0.3% iron trichloride and incubated at 37 °C for 60 min. Absorbance reading was performed at 525 nm in a microplate reader (Synergyx Mx, Biotek, Winooski, VT, USA). The results were calculated concerning the AsA standard curve and expressed as AsA mg·g^−1^ dry matter (FM).

### 4.4. Identification and Retrieval of Genes Associated with AsA Biosynthesis

The genes encoding the 4 AsA biosynthesis pathways (D-mannose/L-galactose, L-gulose, D-galacturonate, Myo-inositol) corresponding to GDP-mannose pyrophosphorylase (GMP), GDP-L-galactose phosphorylase (GGP), L-galactose-1-P phosphatase (GPP), L-galactose dehydrogenase (GalDH), L-galactono-1,4-lactone dehydrogenase (GalLDH), GDP-D-mannose 3′,5′-epimerase (GME), L-gluconolactone oxidase (GulLO), galacturonate reductase (GalUR), and Myo-inositol oxygenase (MIOX) were primarily identified in the genomic and cDNA data of *Arabidopsis* from the GenBank database (http://www.ncbi.nlm.nih.gov) accessed on 1 April 2023. Subsequently, via BLASTn (E-value threshold: 1 × 10^−1^), their orthologous cDNAs were searched in the *G. max* genome, also available in the GenBank database. The cDNAs and corresponding proteins for each gene were retrieved from RefSeq mRNA and/or protein databases and used in gene expression and phylogenetic analyses, respectively. Detailed information about the sequences is provided in Supplementary Table S1.

### 4.5. Phylogenetic Analyses

The corresponding proteins of each gene (41) encoding AsA biosynthesis pathways in *G. max* and their homologous protein sequences (26) of *Arabidopsis. thaliana* along *Amborella trichopoda* sequences (outgroup) were uploaded to the Mega 11 program using the neighbor-joining (NJ) method [71]. The protein sequence alignment was performed using the Muscle software embedded in MEGA11 version 0.1. The parameters for the NJ method were as follows: bootstrap method with 1000 replicates (971 were successful), substitution type “amino acids”, substitution model “Poisson model”, rates among sites “uniform”, and gaps/missing data treatment “pairwise deletion”.

### 4.6. In Silico Expression Analyses of AsA Pathway-Linked Genes in G. max RNA-seq Data

Expression analyses of genes encoding GMP, GGP, GPP, GalDH, GalLDH, GME, GulLO, GalUR, and MIOX were performed using Magic Blast [39] in a diverse set of soybean RNA-seq data obtained from the Sequence Read Archive (SRA) at NCBI GenBank. The evaluated SRA Bioproject were sourced from various tissues of *G. max*, including seeds, roots, stems, and leaves, across developmental stages and stress conditions. The conditions included both biotic (such as salt, water deficit, and submergence) and abiotic (including *Phakopsora pachyrhizi*, *Heterodera glycines*, *Tetranychus cinnabarinus*, *Fusarium virguliforme*) stresses and hormone treatments (e.g., paclobutrazol, PBZ). Supplementary Table S2 contains detailed information about each RNA-seq datum (transcriptome/bioproject) evaluated in this study. To achieve high alignment accuracy, the “word length” was set to a minimum value of 48 to enhance the Magic Blast mapping specificity. The mapped reads of each cDNA were counted using HTSeq2 version 2.0 [40]. Then, the number of mapped reads was normalized using the RPKM (reads per kilobase of transcript per million of mapped reads) method [41].

### 4.7. Statistical Analyses

Gene expression variance was determined by one-way ANOVA, followed by the Bonferroni test or *t*-test. The differences between data were considered significant at *p* < 0.05. Finally, the log_2_ fold-change (Log2 FC) of RPKM means was used to construct a heatmap using the online public heatmapper tool http://www.heatmapper.ca/expression/ accessed on 1 June 2023. For tissue development, the statistical analyses were performed by comparing data from advanced developmental stages with the initial stage as a reference, while in stress conditions, the variance was calculated by taking the expression values of the control as a reference. To explore general expression levels, the log2 of the RPKM values (log2 RPKM) was used in the heatmap, with scales ranging from 0 to 2.5.

## 5. Concluding Remarks

*G. max* exhibits notable gene duplication events, highlighting its potential as a compelling model species for studying the evolutionary dynamics of AsA synthesis. The higher number of AsA-encoding genes in this species compared to *Arabidopsis* can be attributed to gene duplication events, mainly involving neo/subfunctionalization. Gene expression analyses suggested that the four proposed AsA biosynthesis pathways are actively functioning in *G. max* and revealed a differential regulation of these pathways depending on the circumstances at different periods of the soybean’s life cycle. The D-mannose/L-galactose pathway plays a predominant role in AsA biosynthesis. Nevertheless, the L-gulose, D-galacturonate, and Myo-inositol pathways also contribute to AsA synthesis dependent on tissues, developmental stages, and stress conditions. The expression data revealed that in developing tissues/organs, higher AsA mass fractions could be associated with the upregulation of genes from all four proposed pathways (except for Myo-inositol pathways in leaves). This was particularly observed in young leaves and germinating seeds. Under abiotic stresses, reduced AsA mass fractions were consistent with reduced key transcript levels of the D-mannose/L-galactose pathway; however, gene upregulation of alternative pathways of AsA biosynthesis indicates a fine regulation to maintain AsA levels availability for ROS scavenging activity. RNA-seq data of cultivars/genotypes with contrasting tolerance to biotic stresses also revealed potential stress resistance-linked genes (marked with asterisks in Figure 11). The genes *GMP_2a* from D-mannose/L-galactose, GulLO*_1f* from the L-gulose pathway, and *MIOX_3a* from the Myo-inositol pathway exhibited remarkable overexpression under stress conditions. Despite not being among the major sources of AsA, these analyses also demonstrated that germinating seeds, leaves, and roots of *G. max* contained adaptive fractions of AsA in dry masses. These findings together are promising for future experimental studies that aim to validate the highlighted candidate genes for their use in functional marker-assisted plant holobiont selection [72] or bioengineering strategies to improve plant stress resilience and plant resources for vitamin C.

## Data Availability

Data supporting the findings of this study are available within the article and Supplementary Material. The evaluated Bioprojects can be reached using the given accession number in Supplementary Table S2.

## Supplementary Materials

The following supporting information can be downloaded at https://www.mdpi.com/article/10.3390/ijms26104678/s1. References [73,74,75,76,77,78,79,80,81,82,83,84] are cited in the supplementary materials.
