# Supplementary material for: Tissue- and Condition-Specific Biosynthesis of Ascorbic Acid in Glycine max L.: Insights from Genome-Wide Analyses of Pathway-Encoding Genes, Expression Profiling, and Mass Fraction Determination
Source: Int J Mol Sci. 2025 May 14;26(10):4678. doi: 10.3390/ijms26104678 (PMC12111785; doi:10.3390/ijms26104678)
Supplement: Supplementary file 1 [file ijms-26-04678-s001.zip › Suppl. Table S1.pdf]

Supplementary Table S1- Distribution and classification of genes involved in AsA biosynthesis pathways in *Glycine max* using *Arabidopsis thaliana* homologous genes as reference.

| | | *Arabidopsis thaliana* | *Glycine max* | |
|---|---|---|---|---|
| AsA biosynthesis pathways | Genes | Gene members [Accession in NCBI] (Locus Gene ID) | Gene members [accession in NCBI] (Locus Gene ID) | Gene members (name based in phylogeny) |
| D-mannose / L-galactose | GDP-mannose pirofosforilase (GMP) | At_GMP [BT000697.1] (AT2G39770) | Gm_GMP1 [NM_001289311.2] (GLYMA_14G065900) | GMP_1a |
| | | At_GMP [DQ056626.1] (AT3G55590) | GMP1_like [NM_001289330.2] (GLYMA_02G250800) | GMP_1b |
| | | At_GMP [AY142530.1] (AT4G30570) | | GMP_1c |
| | | | GMP2 [XM_014763491.3] (GLYMA_11G223700) | GMP_2a |
| | | | GMP [NM_001255333.2] (GLYMA_18G034400) | GMP_2b |
| | | At_GMP_alpha-A [NM_179555.3] (AT1G74910) | GMP_alpha [XM_003523011.5] (GLYMA_04G178400) | GMP_alpha_A |
| | | | GMP_alpha-B [XM_026128930.1] (GLYMA_06G186400) | GMP_alpha_B |
| | | At_GMP_alpha-C [NM_001335239.1] (AT2G04650) | GMP_alpha [XM_003553202.5] (GLYMA_19G050100) | GMP_alpha_C |
| | GDP-L-galactose phosphorylase | At_GGP [NM_118819.3] (AT4G26850) | GGP1-Like [NM_001254055.3] (GLYMA_14G021800) | GGP_1a |
| | | At_GGP [NM_124894.5] (AT5G55120) | GGP1 [XM_003519540.5] (GLYMA_02G292800) | GGP_1b |
| | | | GGP1 [XM_003551824.4] (GLYMA_18G092900) | GGP_1LikeA |
| | | | GGP1-Like [XR_001389622.3] (LOC100803717) | GGP_1LikeB |
| | L-galactose-1-P phosphatase (inositol monophosphatase) | At_IMP [NM_111155.3] (AT3G02870) | Gm_IMP [XM_014777349.3] (GLYMA_07G266200) | GPP_L |
| | | At_IMPL1 [NM_102857.2] (AT1G31190) | Gm_IMP-3-Like [NM_001255695.3] (GLYMA_15G115500) | GPP_1 |
| | | At_IMPL2 [NM_001342523.1] (AT4G39120) | Gm_IMP-3-Like [NM_001255373.3] (GLYMA_09G011100) | GPP_2 |
| | L-galactose dehydrogenase | At_GalDH [NM_119525.6] (AT4G33670) | Gm_GalDH [NM_001254312.2] (GLYMA_08G064100) | GalDH_1a |
| | | | Gm_GalDH [XM_003528360.5] (GLYMA_07G185100) | GalDH_1b |
| | L-galactono-1,4-lactone dehydrogenase | At_GalLDH [NM_114662.3] (AT3G47930) | Gm_GalLDH [XM_003535854.5] (GLYMA_10G104100) | GalLDH_1a |
| | | | Gm_GalLDH [NM_001249443.3] (L GLYMA_02G166300) | GalLDH_1b |
| | | At_GME | Gm_GME1 [NM_001289329.2] | GME_1a |

| | | | | |
|---|---|---|---|---|
| D-mannose / L-galactose + L-gulose | GDP-D-mannose 3',5'-epimerase | [NM_001203488.1]<br>(AT5G28840) | (GLYMA_19G244600) | |
| | | | Gm_GME2<br>[XM_041014232.1]<br>(GLYMA_03G247000) | GME_1b |
| | | | Gm_GME2<br>[XM_006606397.4]<br>(GLYMA_10G104100) | GME_2a |
| | | | Gm_GME-like<br>[NM_001255835.2]<br>(GLYMA_10G162000) | GME_2b |
| L-gulose | L-gulonolactone oxidase (D-arabinono-1,4-lactone oxidase) | At_GulLO1<br>(NM_102963.2)<br>(AT1G32300) | Gm_GulLO-6<br>[XM_003554734.5]<br>(GLYMA_19G259300) | GulLO_1a |
| | | | Gm_GulLO-6<br>[XM_003521776.5]<br>(GLYMA_03G260600) | GulLO_1b |
| | | | Gm_GulLO-6<br>[XM_003548358.5]<br>(GLYMA_16G020100) | GulLO_1c |
| | | | Gm_GulLO-6<br>[XM_003548356.5]<br>(GLYMA_16G020000) | GulLO_1d |
| | | | Gm_GulLO-4<br>[XM_006604847.4]<br>(GLYMA_19G259400), | GulLO_1e |
| | | | Gm_GulLO-6<br>[XM_003548136.5]<br>(GLYMA_16G019900) | GulLO_1f |
| | | | Gm_GulLO-4<br>[XM_003529830.5]<br>(GLYMA_07G050900) | GulLO_1g |
| | | At_GulLO2<br>[NM_130241.3]<br>(AT2G46750) | | GulLO_2 |
| | | At_GulLO3<br>[NM_121192.2]<br>(AT5G11540) | Gm_GulLO-3<br>[XM_003551100.5]<br>(GLYMA_17G195100) | GulLO_3 |
| | | At_GulLO4<br>[NM_125032.4]<br>(AT5G56490) | | GulLO_4 |
| | | At_GulLO5<br>[NM_130240.3]<br>(AT2G46740) | | GulLO_5 |
| | | At_GulLO6<br>[NM_130242.2]<br>(AT2G46760) | | GulLO_6 |
| | | At_GulLO7<br>[NM_125030.1]<br>(AT5G56470) | | GulLO_7 |
| D-galacturonate | (Galacturonate reductase)<br>Aldo/keto reductase; chalcone reductase | Aldo/keto reductase<br>[NM_104687.1]<br>(AT1G59950) | CHR1 or CHR6<br>[NM_001367007.1]<br>(GLYMA_02G307300) | GalUR_1 |
| | | Aldo/keto reductase<br>[NM_104688.4] (AT1G59960) | CHR2 or CHR1<br>[NM_001249044.2]<br>(GLYMA_14G005700) | GalUR_2 |
| | | | CHR<br>[XM_003547504.4]<br>(GLYMA_15G189300) | GalUR_3 |
| | | | CHR4<br>[NM_001367005.1]<br>(GLYMA_20G031100) | GalUR_4 |
| | | | CHR5<br>[NM_001367006.1]<br>(GLYMA_18G285800) | GalUR_5 |
| | | MIOX1<br>[NM_101319.4]<br>(AT1G14520) | MIOX1<br>[NM_001369341.1]<br>(GLYMA_07G013900v4) | MIOX_1a |

| | | | | |
|---|---|---|---|---|
| Myo-inositol | myo-inositol oxygenase | | MIOX1 [XM_014779129.3] (GLYMA_08G199300) | MIOX_1b |
| | | MIOX2 [NM_127538.4] (AT2G19800) | MIOX2 [XM_003529019.4] (GLYMA_07G126600) | MIOX_2a |
| | | | MIOX [NM_001255706.2] (GLYMA_05G224500) | MIOX_2b |
| | | | MIOX2-Like [NM_001254143.2] (GLYMA_01G005100) | MIOX_3a |
| | | | MIOX2-Like [NM_001254143.2] (GLYMA_01G005100) | MIOX_3b |
| | | MIOX4 NM_118759.5 (AT4G26260) | | |
| | | MIOX5 NM_125047.3 (AT5G56640) | | |
| | | | | |
| **Protein sequences from Amborella trichopoda were used as outgroup in the phylogenetic analyses** | | | | |
| D-mannose / L-galactose | | XP_006846369.1 mannose-1-phosphate guanylyltransferase 1 [Amborella trichopoda] | | Amt_GMP_1a |
| | | XP_006846372.1 mannose-1-phosphate guanylyltransferase 1 [Amborella trichopoda] | | Amt_GMP_1b |
| | | XP_020523187.1 mannose-1-phosphate guanyltransferase alpha isoform X1 [Amborella trichopoda] | | Amt_GMP_Alpha |
| | | XP_006852721.1 L-galactose dehydrogenase [Amborella trichopoda] | | Amt_GalDH |
| | | | | |
| L-gulose | | XP_006846581.3 probable L-gulonolactone oxidase 6 isoform X1 [Amborella trichopoda] | | Amt_GulLO_1 |
| | | XP_006833405.1 L-gulonolactone oxidase 3 isoform X1 [Amborella trichopoda] | | Amt_GulLO_3 |
| | | XP_020520578.1 L-gulonolactone oxidase 2 isoform X1 [Amborella trichopoda] | | Amt_GulLO_2 |
| | | ERN02007.1 hypothetical protein AMTR_s00045p00092460 [Amborella trichopoda] | | Amt_GulLO_4 |
| | | | | |
| D-galacturonate | | ERN11058.1 hypothetical protein AMTR_s00024p00110840 [Amborella trichopoda] | | Amt_GalUR_1 |
| | | XP_006849478.2 non-functional NADPH-dependent codeinone reductase 2 [Amborella trichopoda] | | Amt_GalUR_2 |
| | | XP_011625257.1 non-functional NADPH-dependent codeinone reductase 2 [Amborella trichopoda] | | Amt_GalUR_3 |
| | | XP_006849594.1 non-functional NADPH-dependent | | Amt_GalUR_4 |

| | | | | |
|---|---|---|---|---|
| | | codeinone reductase 2 [Amborella trichopoda] | | |
| | | | | |
| Myo-inositol | | XP_006853784.2 inositol oxygenase 1 isoform X1 [Amborella trichopoda] | | Amt_MIOX_2 |
| | | XP_020528791.1 inositol oxygenase 1 [Amborella trichopoda] | | Amt_MIOX_1 |
