# Supplementary material for: Tissue- and Condition-Specific Biosynthesis of Ascorbic Acid in Glycine max L.: Insights from Genome-Wide Analyses of Pathway-Encoding Genes, Expression Profiling, and Mass Fraction Determination
Source: Int J Mol Sci. 2025 May 14;26(10):4678. doi: 10.3390/ijms26104678 (PMC12111785; doi:10.3390/ijms26104678)
Supplement: Supplementary file 1 [file ijms-26-04678-s001.zip › Suppl. Table S2.pdf]

Supplementary Table S2. Bioproject details of RNA-seq experiments used to obtain the gene expression in *G. max*

Physiological stage/ treatment	BioProject acession	Experiment details	Tissue/Cell type	Biologica l replicates	Reference
	PRJNA449429	Seeds were germinated in water or paclobutrazol (PBZ) at 28°C with 12/12h photoperiod (dark/light) and harvested at 12, 24 and 36 hours after imbibition (HAI).	Seed (Embryonic axis)	Three	Gazara et al., 2019
	PRJNA388955	Transcriptome analysis of soybean (Glycine max) embryos at four seed developmental stages (cotyledon, early, mid and late maturation), mature dry seeds, and seedlings [eight days after seed sowing (DAS)]. We have analyzed the gene expression only in mature dry seeds and seedlings (8 DAS).	Seeds (Embryo)	Three	Data are published only in SRA database from GenBank (NCBI)
	PRJNA262564	Time course Expression analysis of Cotyledon Development and Leaf Development using RNA-seq on distinct time points in Williams-82 plants. For cotyledons, 3 stages were sequenced [4, 15 and 27 days after germination (DAG)]. For leaves, 5 stages were sequenced. Stage 1 (leaves at V3 stage), stage 2 (21 days after stage 1), stage 3 (40 days after stage 1), stage 4 (49 days after stage 1), stage 5 (56 days after stage 1). Stages 4 and 5 were marked by the appearance of signs of leaf senescence.	Leaves and Cotyledons	Three	Brown and Hudson, 2015
Seed Development	PRJNA395215	Developing seeds for 4 soybean experimental lines designated as: (i) 3mlpa, (ii) 3MWT, (iii) 1mlpa, and (iv) 1MWT were sequenced. Five stages based on seed lengths were selected. They corresponded to 2-4 mm (stage1), 4-6 mm (stage2), 6-8 mm (stage3), 8-10 mm (stage4), and 10-12 mm (stage5), respectively.	Seed	Three	Redekar et al., 2017
Abiotic stress	PRJNA432861	Salt-treatment (0.9%) soybean seedlings, on leaf and root in a time-course experiment (0h, 1h, 2h, 4h, 24h and 48h). Soybean plants (C08 genotype) under hydroponic conditions at V1 stage were used.	Leaves /Roots	Three	Data are published only in SRA database from GenBank (NCBI)
	PRJNA259941	Water deficit at times 0h, 6h, 12h, and 24h. This study was performed in leaves of two contrasting genotypes, Benning (drought sensitive) and PI416937 (drought tolerant).	Leaves	Three	Data are published only in SRA database from GenBank (NCBI)
	PRJNA574626	This study was carried out in in leaves and roots of soybean plants (Williams 82) under submergence and drought followed by recovery. Submergence at times 0d (control), 1d, 2d and 3d; drought at times 0d (control), 5d and 6d. The recovery was evaluated 1 day after stress interruption. Plants at V1 stage were used.	Leaves /Roots	Three	Data are published only in SRA database from GenBank (NCBI)
	PRJNA246058	The salt treatment was applied by transferring the seedlings into 100 mM NaCl solution. For the dehydration treatment, plants were removed from the germination paper and left in air under water-limiting conditions to impose dehydration stress. Root tissues were harvested after 0 (control), 1, 6 and 12 hr of stress treatments. Plants (Williams 82) at v1 stage (first trifoliolate stage) were used.	Roots	Three	Belamkar et al., 2014
		Transcriptomic data from a time course comparison of SMV-G7-infected			

Biotic stress	PRJNA564957	resistant (L29, Rsv3-genotype) and susceptible (Williams82, rsv3-genotype) soybean cultivars. Plants at first, fully, expanded trifoliolate stage were inoculated with SMV strain G7 (SMV-G7). Unifoliolate leaves were dusted with carborundum powder and rubbed with SMV-G7 inoculum. The inoculated unifoliolate leaves were collected at 0 (control), 2, 4, 6, and 8 hours post inoculation (hpi) and stored at -80°C.	Leaves	Two	DeMers et al., 2020
	PRJDB7011	Transcriptome data of cultivars BRS184 and NIL (Rpp3) containing (Rpp3 resistance gene of FT2) treated with Soybean rust (Phakopsora pachyrhizi) and mock infection. Samples were collected at time point 6 hours after the beginning of light condition and 24 hours after inoculation with three biological replicates at stage V2.	Leaves	Three	Hossain et al., 2018
	PRJNA515005	Transcriptional responses of soybean (var. S15-L5) to two neonicotinoid insecticides, thiamethoxam applied as seed treatments and imidacloprid applied as soil drench. A subset of these plants were also exposed to spider mite to study the effects of neonicotinoid insecticides on plant-arthropod interactions. Three weeks after germination, twelve untreated soybean plants at V3 stage were randomly chosen and exposed to a soil drench using 0.024 g/100mL of water. Spider mite (T. cinnabarinus) colonies were established naturally in greenhouse infestations. Mites were maintained in plants at least 3 generations before the onset of experiments.	Leaves	Three	Wulff et al., 2019
	PRJNA514200	Two cultivars of soybean, Williams 82 (PI518671) and MN1806CN were used in this experiment. PI518671 is susceptible to both HG Type 0 (race 3) of the *Heterodera glycines* (SCN) and Soybean aphid (SBA). MN1806CN is resistant to HG Type 0 (race 3) of the SCN but susceptible to SBA. Samples (roots) of control (mock), SCN, SBA and SCN-SBA were collected at 5 and 30 days after infestation.	Roots	Three	Neupane et al., 2019
	PRJNA534069	Two Soybean (Glycine max) near-isogenic lines (NILs), TN09-16 (NIL-S) and TN09-29 (NIL-R), which exhibit susceptible and resistant responses, respectively, to SCN (cyst nematode) HG type 0 (race 3) were used in this experiment. Surface-sterilized soybean seeds were germinated on wet germination paper in the dark at 26°C for 3 d. Healthy 3-d-old seedlings were inoculated with c. 3000 second-stage juvenile (J2s) of SCN HG type 0 (race 3) per seedling, by spreading the nematodes across the whole root. At 5 d post-SCN inoculation, roots tissues were collected from both inoculated and no inoculated soybean roots in three biologically independent replicates.	Roots	Three	Rambani et al., 2020
	PRJNA549915	Germinated soybean seedlings (cv Sloan) were sprayed with spores of asexual macroconidia (*Fusarium virguliforme*) and placed in a growth chamber at 12°C for 7 d and then 25°C for 7 d. Thus, samples (roots) of six pooled plants for mock and inoculated were collected for RNA isolation at 0, 2, 4, 7, 10, and 14 DAI.	Roots	Three	Baetsen-Young et al., 2020
	PRJNA631275	Soybean (Glycine max) plants from the cultivar Williams 82 were grown in the	Flower, Opened flower, Unopened flower,		Arikit et al., 2014

		greenhouse under 16 h light/8 h dark at 25°C. RNA-seq was used to characterize gene expression in soybean from a wide range of tissues.	Anther, Ovary, Seed stage 1, Seed stage 2, Seed cotyledon stage , Pod 1 cm, Pod shell 10DAF, Pod Shell 14DAF, Young Leaf, Leaf V3, Nodule, Root	N/A	
	PRJNA326110	Soybean seeds were germinated in soaked cotton at 28ºC. In addition to dry seeds, seeds were harvested at 3, 6, 12, 24 hours after imbibition (HAI).	Seed (Embryonic axis)	Two	Bellieny-Rabelo et al., 2016
