# Supplementary material for: Tissue- and Condition-Specific Biosynthesis of Ascorbic Acid in Glycine max L.: Insights from Genome-Wide Analyses of Pathway-Encoding Genes, Expression Profiling, and Mass Fraction Determination
Source: Int J Mol Sci. 2025 May 14;26(10):4678. doi: 10.3390/ijms26104678 (PMC12111785; doi:10.3390/ijms26104678)
Supplement: Supplementary file 1 [file ijms-26-04678-s001.zip › Suppl. Table S3.pdf]

Supplementary Table S3. RPKM values of the expression of genes associated with AsA biosynthesis pathways in *Glycine max*

	Bioproject: PRJNA631275														
	Flower	Unopened flower	opened flower	Anther	Ovary	Seed stage 1	Seed stage 2	Seed cotyledon stage	pod 1 cm	pod shell – 10 DAF	pod shell- 14 DAF	young leaf	leaf V3	nodule	root
Gm_GMP_1a	41.36	47.98	37.12	10.62	30.89	12.18	5.88	18.14	56.68	52.74	72.80	42.56	120.28	14.23	59.18
Gm_GMP_1b	25.97	39.62	29.64	11.68	28.68	10.38	7.52	18.79	28.84	54.62	35.92	30.41	89.08	10.19	29.71
Gm_GMP_2a	8.27	15.32	11.52	12.89	13.85	1.32	1.98	5.08	10.03	13.10	13.72	8.19	14.89	3.18	10.56
Gm_GMP_2b	8.96	19.06	11.83	24.55	16.26	5.42	5.82	5.62	12.43	19.88	15.03	7.33	16.31	1.95	8.20
Gm_GMP_alpha_A	14.53	12.91	12.50	6.66	10.15	5.85	2.85	3.45	27.31	15.91	22.23	13.66	26.68	1.90	4.59
Gm_GMP_alpha_B	11.97	19.43	9.10	15.71	14.92	8.83	3.37	7.36	35.03	10.48	17.74	16.12	30.72	3.99	4.41
Gm_GMP_alpha_C	4.86	7.13	5.03	2.84	7.76	2.64	1.43	2.66	3.68	4.54	4.46	4.77	9.64	4.35	5.35
Gm_GGP_1likeA	30.87	37.42	46.81	28.00	32.16	28.75	10.35	46.23	19.75	17.47	16.67	32.80	46.93	10.15	27.25
Gm_GGP_1likeB	9.89	20.35	21.02	7.35	54.13	8.06	3.00	16.47	10.83	10.73	9.71	11.58	16.55	8.54	5.98
Gm_GGP_1a	84.57	170.78	246.39	32.62	115.56	15.33	10.20	34.50	55.76	69.89	58.03	121.79	218.18	16.24	9.02
Gm_GGP_1b	74.29	167.61	320.06	37.86	86.21	8.20	5.36	13.79	36.57	44.69	36.45	59.49	171.93	10.03	12.37
Gm_GPP_1	8.11	32.45	26.95	14.11	28.14	11.21	7.93	8.88	5.92	4.03	3.73	12.38	24.08	6.49	6.69
Gm_GPP_2	1.04	8.88	8.15	2.31	15.35	1.18	0.37	1.91	2.24	2.46	1.61	6.26	13.63	0.68	3.60
Gm_GPP_L	1.11	3.30	4.17	1.27	5.88	1.83	1.13	0.79	2.44	2.28	1.89	1.95	1.39	0.72	6.07
Gm_GalDH_1a	16.47	18.46	11.40	4.25	16.81	16.67	17.11	11.53	22.94	43.27	29.48	32.04	26.49	17.26	19.13
Gm_GalDH_1b	1.69	0.27	1.06	0.08	0.85	6.12	0.47	8.46	0.53	0.55	1.01	1.48	1.01	0.00	0.00
Gm_GalLDH_1a	6.12	8.68	4.66	2.04	9.39	4.69	2.02	6.49	9.92	6.05	7.23	11.07	9.57	4.28	4.78
Gm_GalLDH_1b	3.35	3.31	2.98	0.73	4.93	1.34	1.40	1.71	3.16	2.72	2.37	5.47	7.96	1.40	1.71
Gm_GME_1a	25.68	59.36	52.35	6.44	35.56	8.00	6.85	15.47	36.74	30.73	38.62	41.95	89.79	9.32	3.15
Gm_GME_1b	7.05	23.37	14.26	1.96	14.85	4.26	2.30	4.11	3.61	1.63	4.20	5.47	6.78	0.16	0.08
Gm_GME_2a	31.60	47.04	92.38	10.61	78.59	5.64	3.24	12.94	32.94	46.08	50.11	29.87	80.08	12.14	12.90
Gm_GME_2b	29.29	34.74	53.68	10.24	44.16	12.10	6.07	8.49	25.82	33.50	31.81	33.15	70.86	7.81	22.44
Gm_GulLO_1a	0.00	0.00	0.00	0.00	0.00	0.00	0.00	0.00	0.00	0.00	0.00	0.00	0.00	0.60	9.77
Gm_GulLO_1b	1.07	1.81	0.29	0.97	0.25	0.00	0.07	0.00	0.62	0.96	1.04	0.94	0.94	3.60	7.70
Gm_GulLO_1c	0.49	0.50	0.33	0.02	0.62	0.24	0.07	0.18	0.39	0.16	0.37	0.43	1.65	2.58	2.23
Gm_GulLO_1d	0.00	3.09	3.98	3.17	4.31	0.09	0.00	0.00	0.00	0.00	0.00	0.00	4.96	1.35	0.12
Gm_GulLO_1e	0.53	0.13	0.25	0.00	0.06	0.00	0.00	0.00	0.25	0.95	0.40	0.00	0.41	0.41	0.00
Gm_GulLO_1f	1.41	0.05	0.54	0.05	0.12	0.00	0.00	0.00	0.14	0.14	0.07	0.00	0.19	0.15	3.55
Gm_GulLO_1g	0.00	0.09	0.13	0.08	0.00	0.00	0.00	0.00	0.35	0.27	0.25	0.49	0.81	0.06	0.00
Gm_GulLO_3	7.00	3.03	13.13	0.72	1.82	0.93	0.46	0.34	4.60	2.76	4.26	3.04	2.93	1.13	17.02
Gm_GalUR_1	0.15	0.09	0.58	0.02	0.12	0.00	0.00	0.00	0.00	0.00	0.14	0.81	0.00	2.32	65.56
Gm_GalUR_2	5.27	1.16	9.76	0.17	0.76	0.00	0.00	0.31	3.73	2.57	2.38	0.39	0.96	25.70	190.09
Gm_GalUR_3	0.00	0.02	0.00	0.00	0.02	0.00	0.00	0.00	0.00	0.00	0.00	0.00	0.02	0.00	0.00
Gm_GalUR_4	3.77	4.44	4.40	1.78	3.47	0.79	0.67	1.47	1.94	2.22	1.96	1.81	9.68	1.64	7.76
Gm_GalUR_5	3.14	1.27	4.59	0.06	0.34	0.00	0.00	0.00	4.84	4.87	11.97	11.42	0.36	17.05	281.78
Gm_MIOX_1a	13.67	8.95	13.90	1.69	0.97	0.80	0.38	0.25	6.07	6.83	14.92	4.34	0.08	3.60	0.88
Gm_MIOX_1b	47.19	17.66	38.42	3.46	0.30	1.44	0.37	1.68	1.79	7.30	7.16	0.20	0.06	0.51	3.34
Gm_MIOX_2a	1.61	5.36	4.48	0.52	0.78	0.00	0.00	0.00	3.15	1.81	3.35	22.00	24.51	1.29	0.52
Gm_MIOX_2b	57.78	9.96	54.01	0.81	3.01	5.54	6.97	9.10	5.92	12.11	22.91	0.48	0.16	4.90	2.27
Gm_MIOX_3a	30.19	0.07	10.72	0.02	0.02	0.57	0.00	0.31	0.94	0.00	0.13	0.00	0.37	2.52	0.20
Gm_MIOX_3b	0.13	0.09	0.39	0.02	0.31	0.00	0.00	0.00	0.00	0.00	0.00	0.00	0.02	0.00	0.18
