# Supplementary material for: Tissue- and Condition-Specific Biosynthesis of Ascorbic Acid in Glycine max L.: Insights from Genome-Wide Analyses of Pathway-Encoding Genes, Expression Profiling, and Mass Fraction Determination
Source: Int J Mol Sci. 2025 May 14;26(10):4678. doi: 10.3390/ijms26104678 (PMC12111785; doi:10.3390/ijms26104678)
Supplement: Supplementary file 1 [file ijms-26-04678-s001.zip › Suppl. Table S4.pdf]

Supplementary Table S4. Means of RPKM values ± SD (standard deviation) of AsA biosynthesis transcripts during germination in water and treated with paclobutrazol (PBZ), a gibberellin (GA) biosynthesis inhibitor. Statistical analysis, one-way ANOVA followed by Bonferroni test, was applied to 24 and 36 HAI (hours after imbibition) (water) to 12 HAI (water control) of bioproject PRJNA449429. While statistical analysis, t-test, was applied to seedlings 8 DAI to dry seeds (control) of the bioproject PRJNA388955 and to PBZ concerning respective water controls at times 12, 24, and 36 HAI of the bioproject PRJNA449429. Up- and downregulated genes are in green and red, respectively. Significant differences from the controls are indicated by * at $p < 0.05$.

	**PRJNA 449429**			**PRJNA 388955**		**PRJNA 449429**					
	**12 HAI – water (control)**	**24 HAI _ water**	**36 HAI _ water**	**Dry (control)**	**Seedling 8 DAI**	12 HAI		24 HAI		36 HAI	
						Water (control)	PBZ	Water (control)	PBZ	Water (control)	PBZ
Gm_GMP_1a	18.36±1.71	69.32±4.35*	84.71±15.98*	1±0.16	55.1±4.06*	18.36±1.71	18.39±5.5	69.32±4.35	41.44±6.8*	84.71±15.98	92.92±13.8
Gm_GMP_1b	5.12±0.8	43.39±3.84*	62.71±11.32*	1.54±0.21	22.28±1.84*	5.12±0.8	4.46±1.33	43.39±3.84	23.86±5.6*	62.71±11.32	67.34±5.72
Gm_GMP_2a	4.81±0.48	21.9±0.72*	24.05±5.3*	5.86±0.58	9.43±1.28*	4.81±0.48	4.34±0.99	21.9±0.72	13.15±2.37*	24.05±5.3	24.52±0.72
Gm_GMP_2b	3.59±0.23	12.52±0.45*	13.88±2.43*	2.05±0.47	7.79±0.25*	3.59±0.23	3.26±0.4	12.52±0.45	7.75±1.66*	13.88±2.43	14.48±2.17
Gm_GMP_alpha_A	3.59±0.58	16.01±2.46*	13.1±1.27*	0.56±0.07	10.54±1.28*	3.59±0.58	3.62±0.82	16.01±2.46	10.08±1.85*	13.1±1.27	16.41±1.53
Gm_GMP_alpha_B	3.82±0.23	17.87±2.5*	14.75±0.63*	0.82±0.11	13.03±1.22*	3.82±0.23	2.89±0.51	17.87±2.5	12.6±2.64	14.75±0.63	17.45±2.2
Gm_GMP_alpha_C	4.44±1.34	9.59±0.51*	7.12±0.97*	8.01±1.07	7.69±0.43	4.44±1.34	4.67±0.1	9.59±0.51	6.79±1.43	7.12±0.97	8.94±0.59
Gm_GGP_1likeA	13.74±3.53	31.29±1.04*	36.38±3.54*	51.65±3.15	39.57±2.34*	13.74±3.53	16.08±3.01	31.29±1.04	17.89±2.92*	36.38±3.54	36.16±2.44
Gm_GGP_1likeB	7.71±1.71	13.13±0.8*	8.57±1.26	6.82±1.24	7.35±0.98	7.71±1.71	9.58±1.94	13.13±0.8	7.67±1.42*	8.57±1.26	10.18±0.83
Gm_GGP_1a	14.13±6.1	25.36±3.57*	26.53±2.28*	10.53±2.43	125.72±3*	14.13±6.1	15±4.16	25.36±3.57	19.62±2.48	26.53±2.28	31.13±7.27
Gm_GGP_1b	6.14±3.03	8.96±1.31	14.32±3.6*	23.12±3.81	81.14±4.89*	6.14±3.03	7.48±0.9	8.96±1.31	7.18±1.03	14.32±3.6	19.77±10.93
Gm_GPP_1	6.14±1.98	22.6±1.24*	27.24±1.73*	0.04±0.07	7.23±0.33*	6.14±1.98	5.05±0.62	22.6±1.24	17.81±3.72	27.24±1.73	29.25±4
Gm_GPP_2	1.83±0.38	7.64±0.78*	7.38±1.91*	0.16±0.19	2.73±0.36*	1.83±0.38	1.82±0.34	7.64±0.78	5.62±1.18	7.38±1.91	6.64±0.57
Gm_GPP_L	0.03±0.04	0.79±0.12*	0.72±0.17*	0.01±0.02	0.27±0.03*	0.03±0.04	0.07±0.04	0.79±0.12	0.46±0.08*	0.72±0.17	1.41±0.46
Gm_GalDH_1a	4.37±0.62	19.04±0.69*	25.01±3.93*	1.1±0.22	11.63±0.74*	4.37±0.62	4.24±0.42	19.04±0.69	12.91±1.76*	25.01±3.93	26.51±2.29
Gm_GalDH_1b	0.39±0.14	0.65±0.18	0.67±0.1	0.29±0.49	0.59±0.15	0.39±0.14	0.21±0.01	0.65±0.18	0.44±0.14	0.67±0.1	0.63±0.26
Gm_GalLDH_1a	3.92±0.84	16.21±0.65*	15.31±1.16*	0.64±0.19	7.73±0.44*	3.92±0.84	4.35±0.57	16.21±0.65	9.48±0.82*	15.31±1.16	15.8±0.7
Gm_GalLDH_1b	2.53±0.59	8.77±0.53*	8.62±1.83*	0.41±0.13	6.23±0.41*	2.53±0.59	3.09±0.7	8.77±0.53	5.17±0.68*	8.62±1.83	9.86±0.57
Gm_GME_1a	54.54±14.53	37.39±6.08	33.24±5.37	28.79±1.42	15.6±3.19*	54.54±14.53	67.25±5.83	37.39±6.08	25.82±7.72	33.24±5.37	41.84±1.99
Gm_GME_1b	9.22±2.61	11.49±0.64	8.65±1.43	3.17±0.3	43.06±2.68*	9.22±2.61	11.74±2.47	11.49±0.64	7.68±0.92*	8.65±1.43	9.66±1.04
Gm_GME_2a	18±3.37	28.23±2.53	30.61±7.28*	130.03±8.62	35.25±2.93*	18±3.37	21.31±3.84	28.23±2.53	16.67±2.19*	30.61±7.28	33.57±6.71
Gm_GME_2b	11.71±1.14	20.99±1.93*	21.03±6.8	38.02±4.45	33.57±2.13	11.71±1.14	13.78±1.92	20.99±1.93	13.84±2.99*	21.03±6.8	20.18±1.89
Gm_GulLO_1a	0.01±0	0.26±0.04*	0.06±0.02	0.01±0	2.01±0.1*	0.01±0	0.02±0.01	0.26±0.04	0.21±0.04	0.06±0.02	0.36±0.36
Gm_GulLO_1b	0.23±0.13	4.2±0.63*	4.9±0.62*	0.01±0.02	1.11±0.29*	0.23±0.13	0.15±0.04	4.2±0.63	2.45±0.46*	4.9±0.62	7.75±0.79*
Gm_GulLO_1c	0.61±0.13	3.62±0.36*	5.72±1.02*	0.01±0.02	0.61±0.15*	0.61±0.13	0.52±0.1	3.62±0.36	2.07±0.24*	5.72±1.02	6.59±1.05
Gm_GulLO_1d	0.03±0.02	0.05±0	0.04±0.02	0.02±0.04	0±0.01	0.03±0.02	0.09±0.03*	0.05±0	0.02±0.02	0.04±0.02	0.08±0.02
Gm_GulLO_1e	0.07±0.03	0.26±0.02*	0.25±0.08*	0.08±0.01	0±0.01	0.07±0.03	0.06±0.01	0.26±0.02	0.05±0.01*	0.25±0.08	0.45±0.14
Gm_GulLO_1f	0.01±0.01	0.15±0.01*	0.12±0.03*	0.01±0.02	6.73±0.76*	0.01±0.01	0.04±0.01	0.15±0.01	0.1±0.02*	0.12±0.03	0.11±0.04
Gm_GulLO_1g	0±0	0.01±0.01	0.01±0.02	0.01±0.02	0.16±0.03*	0±0	0±0	0.01±0.01	0±0	0.01±0.02	0.01±0.01
Gm_GulLO_3	0.87±0.05	11.12±1.03*	14.12±1.22*	0.01±0.02	5.46±0.31*	0.87±0.05	1.01±0.32	11.12±1.03	4.31±0.65*	14.12±1.22	12.88±0.39
Gm_GalUR_1	2.56±0.43	6.46±1.29*	7.74±0.97*	0±0	8.48±1.26*	2.56±0.43	3.06±0.59	6.46±1.29	3.88±0.56	7.74±0.97	4.44±1.35*

Gm_GalUR_2	19.35±3.87	56.25±6.8*	57.11±11.11*	0.78±0.17	51.15±11.76*	19.35±3.87	25±4.44	56.25±6.8	41.09±5.17	57.11±11.11	78.4±11.48
Gm_GalUR_3	0±0	0±0	0.02±0.03	0±0	0.07±0.02	0±0	0±0	0±0	0±0	0.02±0.03	0±0
Gm_GalUR_4	4.65±1.02	8.34±0.25*	6.19±1.36	5.3±0.63	8±0.46*	4.65±1.02	5.69±1.39	8.34±0.25	5.78±0.96*	6.19±1.36	7.54±0.37
Gm_GalUR_5	27.01±5.84	132.57±10.37*	133.03±25.41*	0.04±0.07	24.54±5.31*	27.01±5.84	29.04±4.42	132.57±10.37	73.45±5.42*	133.03±25.41	156.78±12.91
Gm_MIOX_1a	0.63±0.22	1.35±0.21*	0.8±0.11	18.99±3.49	8.55±0.53*	0.63±0.22	0.88±0.17	1.35±0.21	1.02±0.29	0.8±0.11	2.75±2.38
Gm_MIOX_1b	1.87±1.03	4.63±0.63*	11.08±4.64*	88.09±3.82	39.52±1.99*	1.87±1.03	2.33±0.46	4.63±0.63	2.34±0.45*	11.08±4.64	11.14±4.09
Gm_MIOX_2a	0±0	0±0	0.1±0.02	0.01±0.02	16.43±2.1*	0±0	0±0	0±0	0±0	0.1±0.02	0.02±0.03*
Gm_MIOX_2b	1.8±0.2	6.23±0.98*	12.93±6.05*	2.37±0.42	122.47±5.71*	1.8±0.2	1.59±0.12	6.23±0.98	2.09±0.44*	12.93±6.05	11.46±2.47
Gm_MIOX_3a	0±0	0±0	0±0	0.33±0.08	1.8±0.64	0±0	0±0	0±0	0±0	0±0	0±0
Gm_MIOX_3b	0.06±0.05	0.03±0.05	0.01±0.02	0.07±0.12	0±0	0.06±0.05	0.13±0.02	0.03±0.05	0.02±0	0.01±0.02	0.01±0.02
