# Supplementary material for: Tissue- and Condition-Specific Biosynthesis of Ascorbic Acid in Glycine max L.: Insights from Genome-Wide Analyses of Pathway-Encoding Genes, Expression Profiling, and Mass Fraction Determination
Source: Int J Mol Sci. 2025 May 14;26(10):4678. doi: 10.3390/ijms26104678 (PMC12111785; doi:10.3390/ijms26104678)
Supplement: Supplementary file 1 [file ijms-26-04678-s001.zip › Suppl. Table S5.pdf]

Supplementary Table S5. Means of RPKM values ± SD (standard deviation) of AsA biosynthesis transcripts during the development of leaves, cotyledons, and seeds. Statistical analysis (one_way ANOVA followed by Bonferroni test) was applied to the first stage in bioproject PRJNA262564, to cotyledons at 4 days after germination (4 DAG) in bioproject PRJNA262564 and to stage 1 of each genotype in bioproject PRJNA395215. While statistical analysis (t-test) was applied to the young leaves in bioproject PRJNA631275. Up and downregulated genes are in green and red, respectively. Significant differences from the first stage in each bioproject are indicated by * at $p < 0.05$.

	**PRJNA 631275**		**PRJNA 262564**				
	**Young leaf**	**Leaf (V3 stage)**	**Stage 1 (V3 stage)**	**Stage 3**	**Stage 5**	**Stage 7**	**Stage 8**
Gm_GMP_1a	42.56±0	120.28±2.46*	29.98±5.32	9.38±0.84*	7.55±1.43*	8.08±0.63*	7.34±1.17*
Gm_GMP_1b	30.41±0	89.08±2.77*	29.36±10.6	35.4±6.69	15.55±2.27*	16.93±2.57*	10.79±1.72*
Gm_GMP_2a	8.19±0	14.89±0.42*	6.01±1.14	3.72±0.91*	2.44±0.57*	2.11±0.53*	2.31±0.44*
Gm_GMP_2b	7.33±0	16.31±0.78*	8.98±1.76	3.66±0.52*	2.69±0.33*	3.01±0.41*	4.02±1.12*
Gm_GMP_alpha_A	13.66±0	26.68±0.72*	2.9±0.38	0.47±0.16*	0.28±0.05*	0.09±0.01*	0.07±0.03*
Gm_GMP_alpha_B	16.12±0	30.72±1.36*	4.47±1.14	0.93±0.19*	0.56±0.13*	0.44±0.1*	0.37±0.44*
Gm_GMP_alpha_C	4.77±0	9.64±0.19*	2.11±0.34	2.91±0.61	3.13±0.72	3.46±0.09*	3.94±0.53*
Gm_GGP_1likeA	32.80±0	46.93±0.32*	35.39±3.37	24.89±1.94*	33.24±4.22	36.36±2.84	30.15±5.9
Gm_GGP_1likeB	11.58±0	16.55±1.12*	5.55±0.64	5.4±0.82	6.08±0.99	7.4±0.23	6.76±1.55
Gm_GGP_1a	121.79±0	218.18±0.28*	240.62±7.16	272.67±30.2	542.27±66.52*	644.71±39*	413.47±55.87*
Gm_GGP_1b	59.49±0	171.93±2.93*	192.42±6.22	169.44±27.15	176.91±18.52	188.21±16.77	148.85±9.2*
Gm_GPP_1	12.38±0	24.08±0.77*	27.69±4.34	24.14±8.31	8.67±2.41*	13.31±2.87*	9.85±1.39*
Gm_GPP_2	6.26±0	13.63±0.66*	16.57±6.87	15.35±2.76	1.8±0.53*	3.99±0.29*	2.48±1.13*
Gm_GPP_L	1.95±0	1.39±0.04	2.63±0.74	2.15±1.33	0.29±0.08*	0.79±0.18*	1.34±0.52
Gm_GalDH_1a	32.04±0	26.49±1.23	9.2±2.16	3.66±0.83*	2.33±0.08*	2.79±0.55*	2.11±0.9*
Gm_GalDH_1b	1.48±0	1.01±0.15	0.25±0.07	0±0*	0±0*	0±0*	0±0*
Gm_GalLDH_1a	11.07±0	9.57±0.31	2.74±0.25	2.13±0.6	2.89±0.39	2.81±0.19	1.76±0.47
Gm_GalLDH_1b	5.47±0	7.96±0.00	1.56±0.07	1.54±0.92	1.27±0.32	1±0.17	0.59±0.16
Gm_GME_1a	41.95±0	89.79±1.04*	66.39±13.53	21.3±2.15*	6.86±0.58*	2.95±0.56*	2.44±0.4*
Gm_GME_1b	5.47±0	6.78±0.28	6.2±1.74	0.94±0.16*	0.77±0.19*	0.55±0.1*	0.51±0.14*
Gm_GME_2a	29.87±0	80.08±0.16*	41.67±1.17	47.92±3.41	43.93±2.93	56±2.46*	36.57±7.16
Gm_GME_2b	33.15±0	70.86±2.97*	31.99±3.4	50.35±5.89*	30.11±4.04	25.14±1.87	24.58±6.28
Gm_GulLO_1a	0.00±0	0.00±0.00	0±0	0±0	0±0	0±0	0±0
Gm_GulLO_1b	0.94±0	0.94±0.05	1.03±0.18	0.19±0.03*	0.09±0.01*	0.05±0.03*	0.05±0.04*
Gm_GulLO_1c	0.43±0	1.65±0.31*	0.2±0.01	0±0*	0±0*	0±0*	0±0*
Gm_GulLO_1d	0.00±0	4.96±0.49*	0±0	0±0	0±0	0±0	0±0
Gm_GulLO_1e	0.00±0	0.41±0.07*	0.32±0.06	0.02±0.03*	0±0*	0±0*	0±0*
Gm_GulLO_1f	0.00±0	0.01±0.00	0.01±0.01	0.01±0.01	0.11±0.17*	2.16±0.26*	2.08±0.69*
Gm_GulLO_1g	0.49±0	0.81±0.30	0.03±0.03	0.07±0.02	0.13±0.06*	0.2±0.01*	0.19±0.02*
Gm_GulLO_3	3.04±0	2.93±0.84	1.28±0.16	0.28±0.07*	0.09±0.08*	0.04±0.02*	0.04±0.03*
Gm_GalUR_1	0.81±0	0.00±0.00*	0.06±0.03	0±0	0.05±0.09	0.36±0.32	0.17±0.07
Gm_GalUR_2	0.39±0	0.66±0.20	0.8±0.49	0.35±0.07	1.41±0.33	2.85±0.77*	4.81±1.11*
Gm_GalUR_3	0.00±0	0.02±0.02	0±0	0±0	0±0	0±0	0±0
Gm_GalUR_4	1.81±0	9.68±0.56*	0.78±0.21	0.59±0.05	1.41±0.27	2.66±0.47*	3.15±0.65*
Gm_GalUR_5	11.42±0	0.36±0.04*	0.6±0.21	0.22±0.16	1.79±2.32	2.68±0.38*	3.1±0.9*
Gm_MIOX_1a	4.34±0	0.08±0.09*	0.17±0.05	0.25±0.11	0.3±0.08	0.56±0.08	1.37±0.58*
Gm_MIOX_1b	0.20±0	0.06±0.03*	0.52±0.13	0.87±0.17	0.64±0.14	0.55±0.27	1.08±0.83
Gm_MIOX_2a	22.00±0	24.51±0.53	24.63±7.3	7.61±1.33*	0.3±0.11*	0.27±0.14*	0.21±0.17*
Gm_MIOX_2b	0.48±0	0.16±0.02*	0.16±0.16	0.04±0.01	0±0	0.01±0.02	0.16±0.28
Gm_MIOX_3a	0.00±0	0.07±0.03	0.14±0.01	0±0*	0±0*	0±0*	0.06±0.03*

Gm_MIOX_3b	0.00±0	0.02±0.03	0±0	0±0	0±0	0±0	0.01±0.02

	PRJNA 262564		
	**4 DAG**	**15 DAG**	**27 DAG**
Gm_GMP_1a	11.06±1.46	11.1±0.25	16.41±0.95*
Gm_GMP_1b	13.48±1.52	9.43±1.58*	8.33±0.77*
Gm_GMP_2a	1.69±0.21	4.4±1.02*	6.77±0.67*
Gm_GMP_2b	2.5±0.59	2.93±0.79	5.35±0.11*
Gm_GMP_alpha_A	12.33±1.21	1.11±0.27*	0.95±0.12*
Gm_GMP_alpha_B	13.83±1.42	0.66±0.07*	0.67±0.11*
Gm_GMP_alpha_C	1.89±0.17	5±0.45*	3.9±0.41*
Gm_GGP_1likeA	31.68±2.07	51.04±3.44*	49.1±1.38*
Gm_GGP_1likeB	3.54±0.44	9.04±0.51*	10.66±0.52*
Gm_GGP_1a	58.82±6.9	187.56±2.84*	265.82±31.25*
Gm_GGP_1b	25.32±2.02	103.98±2.85*	89.88±15.5*
Gm_GPP_1	11.86±0.87	4.99±0.78*	4.88±0.16*
Gm_GPP_2	5.12±0.29	3.99±1.16	1.87±0.11*
Gm_GPP_L	0.07±0.05	2.15±0.23*	0.78±0.27*
Gm_GalDH_1a	15.14±1.38	2.84±0.78*	2.27±0.21*
Gm_GalDH_1b	0.51±0.06	0±0*	0.03±0.01*

Gm_GalLDH_1a	4.85±0.29	5.22±0.15	3.65±0.3*
Gm_GalLDH_1b	2.86±0.26	1.48±0.24*	1.73±0.21*
Gm_GME_1a	274.12±28.62	3.19±0.58*	1.05±0.01*
Gm_GME_1b	2.06±0.38	0.58±0.13*	0.22±0.06*
Gm_GME_2a	50.83±2.21	36.07±1.02*	22.95±0.41*
Gm_GME_2b	31.23±2.46	49.98±2.85*	31.5±1.22
Gm_GulLO_1a	0±0	0±0	0±0
Gm_GulLO_1b	1.15±0.22	0.08±0.01*	0.33±0.05*
Gm_GulLO_1c	0.11±0.03	0±0*	0±0*
Gm_GulLO_1d	0±0	0±0	0±0
Gm_GulLO_1e	0.02±0.03	0±0	0±0
Gm_GulLO_1f	0.02±0	0.59±0.22	2.34±0.49*
Gm_GulLO_1g	0.06±0.11	0.01±0.01	0±0
Gm_GulLO_3	0.99±0.09	5.84±1.08	51.01±3.82*
Gm_GalUR_1	0.12±0.05	0.09±0.02	0.04±0.01*
Gm_GalUR_2	2.66±0.65	17.3±3.82*	3.26±0.5
Gm_GalUR_3	0.94±0.19	0±0*	0±0*
Gm_GalUR_4	3.57±0.32	5.87±0.55*	8.5±0.8*
Gm_GalUR_5	1.65±0.61	9.78±2.23*	1.04±0.5
Gm_MIOX_1a	0.12±0.04	0.05±0.01	4.59±0.82*
Gm_MIOX_1b	1.4±0.37	2.13±0.64	13.14±0.62*
Gm_MIOX_2a	0.06±0.05	0±0.01*	0±0*
Gm_MIOX_2b	0.54±0.43	3.68±1.03	24.33±4.2*
Gm_MIOX_3a	0.01±0.01	0±0.01	0.47±0.61
Gm_MIOX_3b	0±0	0±0	0±0

	PRJNA395215				
	3mlpa genotype				
	**Stage 1**	**Stage 2**	**Stage 3**	**Stage 4**	**Stage 5**
Gm_GMP_1a	26.02±4.33	35.77±1.41*	36.43±3.06*	33.39±2.85*	24.69±1.2
Gm_GMP_1b	38.17±3.07	34.74±2.29	35.78±5.43	28.69±5.78	16.84±0.26*
Gm_GMP_2a	12.86±0.8	13.79±0.43	13.68±1.23	11.07±1.09	9.99±0.98*
Gm_GMP_2b	24.63±2.15	29±2.26	26.44±0.72	19.3±3.17*	16.68±0.26*
Gm_GMP_alpha_A	10.07±0.6	10.89±0.74	14.74±2.34	15.86±3.46*	11.71±1.5
Gm_GMP_alpha_B	18.73±1.54	20.18±1.61	26.5±4.92	26.54±4.41	17.34±1.49
Gm_GMP_alpha_C	7.78±0.31	8.21±0.28	7.42±1	6.13±0.9*	4.42±0.18*
Gm_GGP_1likeA	104.63±8.46	81.78±3.76*	52.4±2.9*	42.78±2.89*	23.31±3.5*
Gm_GGP_1likeB	22.53±1.21	16.83±0.5*	10.69±0.72*	8.14±0.5*	6.28±0.53*
Gm_GGP_1a	66.51±4.71	101.09±7.08*	87.25±6.04*	76.45±9.2	87.38±5.73*
Gm_GGP_1b	47.84±3.24	54.55±4.36	59.77±2.48*	42.87±6.55	30.7±2.71*
Gm_GPP_1	38.25±3.16	36.05±2.92	30.49±3.37	30.8±7.78	17.54±2.87*
Gm_GPP_2	5.14±0.54	6.92±1.28	14.39±0.63*	18.91±2.48*	5.67±0.49
Gm_GPP_L	3.2±0.33	2.36±0.19*	2.35±0.22*	1.53±0.4*	0.37±0.02*
Gm_GalDH_1a	15.55±1.23	20.63±0.17	24.14±4.6*	24.31±4.17*	17.33±0.6
Gm_GalDH_1b	4.03±0.71	6.71±0.81*	7.1±1.21*	6.31±1.24	4.15±1.04
Gm_GalLDH_1a	10.22±0.1	12.4±0.53	10.78±1.47	10.47±1.81	8.19±0.04
Gm_GalLDH_1b	4.93±0.58	5.72±0.03	4.83±0.97	4.01±0.71	2.68±0.14*
Gm_GME_1a	22.22±1.03	42.86±3.02*	49.21±9.82*	38.05±5.15*	12.63±2.82
Gm_GME_1b	14.82±1.2	11.4±1.31*	7.29±0.94*	6.16±0.92*	5.04±0.82*
Gm_GME_2a	21.87±1.36	31.08±2.38*	25.01±1.26	18.54±2.8	13.37±1.5*
Gm_GME_2b	24.39±0.68	21.76±2.15	16.06±2.48*	11.28±1.84*	7.76±0.53*
Gm_GulLO_1a	0.02±0.03	0±0.01	0±0	0±0	0.01±0.01
Gm_GulLO_1b	0.17±0.05	0.02±0.01*	0.02±0.02*	0.01±0.02*	0±0*

Gm_GulLO_1c	0.44±0.06	0.55±0.14	0.84±0.05*	0.57±0.09	0.61±0.05
Gm_GulLO_1d	0.06±0.02	0.06±0.02	0.03±0.02	0.07±0.02	0.06±0.06
Gm_GulLO_1e	0.73±0.07	0.28±0.08*	0.55±0.34	0.37±0.1	0.25±0.06*
Gm_GulLO_1f	0.04±0.06	0±0.01	0.02±0.01	0±0.01	0±0
Gm_GulLO_1g	0±0	0.01±0.03	0±0	0±0	0.01±0.01
Gm_GulLO_3	0.72±0.15	0.69±0.16	0.92±0.29	0.87±0.11	1.33±0.09*
Gm_GalUR_1	0.06±0.03	0.65±0.16*	0.94±0.14*	1.52±0.15*	1.4±0.21*
Gm_GalUR_2	0.24±0.11	0.32±0.02	0.92±0.14*	1.27±0.35*	0.77±0.41
Gm_GalUR_3	0±0	0±0	0±0	0±0	0±0
Gm_GalUR_4	3.25±0.31	3.33±0.3	3±0.26	2.8±0.19	2.75±0.41
Gm_GalUR_5	0.15±0.12	0.87±0.14	3.79±0.88	19.02±2.15*	17.39±5.02*
Gm_MIOX_1a	4.73±1.29	1.94±0.5	3.92±2.24	0.45±0.13*	0.57±0.06*
Gm_MIOX_1b	11.95±2.53	2.21±0.7*	3.36±1.7*	0.42±0.07*	0.36±0.04*
Gm_MIOX_2a	0.17±0.12	0.04±0.01	0±0*	0±0*	0±0*
Gm_MIOX_2b	45.54±6.56	28.37±3.36*	37.28±10.67	5.4±1.14*	3.09±0.39*
Gm_MIOX_3a	0.05±0.02	0±0*	0±0*	0±0*	0.01±0.02*
Gm_MIOX_3b	0.02±0.03	0.02±0.03	0.03±0.03	0.03±0.03	0.05±0.04

	PRJNA395215				
	3MWT genotype				
	Stage 1	Stage 2	Stage 3	Stage 4	Stage 5
Gm_GMP_1a	14.91±1.63	25.23±0.51*	33.02±2.01*	27.94±2.74*	24.34±2.11*
Gm_GMP_1b	25.3±2.15	36.31±0.51*	35.33±2.93*	26.86±2.44	18.99±1.9*
Gm_GMP_2a	9.69±1.2	9.02±0.88	10.27±1.09	10.2±0.91	8.94±1.13
Gm_GMP_2b	16.99±0.58	20.91±1.02	20.43±2.41	17.84±1.53	14.35±2.29
Gm_GMP_alpha_A	13.65±1.34	11.2±1.62	14.57±0.98	15.31±1.15	13.15±1.86
Gm_GMP_alpha_B	23.63±1.62	20.94±1.14	23.31±1.19	23.76±2.18	20.86±1.87
Gm_GMP_alpha_C	7.08±0.79	7.01±0.55	6.05±0.42	5.16±0.74*	4.83±0.55*
Gm_GGP_1likeA	89.81±10.4	70.91±1.7*	56.95±4.4*	42.92±5.81*	30.87±3.88*
Gm_GGP_1likeB	23.35±1.36	22.08±1.92	14.45±1.57*	9.42±1.17*	8.08±0.41*
Gm_GGP_1a	38.75±3.62	153.18±8.02*	144.94±5.95*	118.02±5.07*	95.05±5.46*
Gm_GGP_1b	30.35±1.95	81.81±7.08*	71.99±4.41*	42.22±2.33*	30.08±3.16
Gm_GPP_1	68.35±7.84	33.39±1.87*	33.86±2.13*	28.27±2.72*	22.24±2.19*
Gm_GPP_2	5.9±0.57	10.44±1.64*	15.55±2.66*	10.69±1.8*	8.07±1.81
Gm_GPP_L	2.28±0.22	1.91±0.34	2.06±0.31	1.02±0.29*	0.67±0.12*
Gm_GalDH_1a	23.39±2.01	22.98±2.13	28.06±1.08*	23.57±1.74	20.27±2.06
Gm_GalDH_1b	4.65±0.47	6.2±1.36	6.78±0.26	6.99±1.29	4.61±0.99
Gm_GalLDH_1a	10.91±1.39	10.93±0.36	11.57±0.88	11.45±1.03	10.13±0.93
Gm_GalLDH_1b	4.2±0.36	4.83±0.06	4.57±0.13	4.11±0.67	3.02±0.36*
Gm_GME_1a	30.13±5.97	56.86±11.06*	80.54±8.57*	57.06±4.8*	30.02±5.8
Gm_GME_1b	13.79±4.83	10.61±0.36	11.7±0.95	9.82±0.85	7.73±0.35*
Gm_GME_2a	20.2±1.79	36.03±3.14*	34.15±6.48*	23.84±1.78	17.12±0.99
Gm_GME_2b	21.66±4.73	22.15±1.68	20.21±3.9	12.55±1.01*	8.74±0.36*

Gm_GulLO_1a	0.01±0.01	0±0	0±0	0±0	0±0
Gm_GulLO_1b	0.83±0.11	0.12±0.01*	0.01±0*	0±0*	0±0*
Gm_GulLO_1c	0.43±0.15	0.25±0.03	0.38±0.12	0.52±0.14	0.44±0.07
Gm_GulLO_1d	0.04±0.02	0.08±0.02	0.05±0.02	0.05±0.02	0.03±0.02
Gm_GulLO_1e	0.11±0.04	0.14±0.02	0.38±0.1*	0.37±0.07*	0.24±0.13
Gm_GulLO_1f	0±0	0.01±0.02	0±0	0.01±0.01	0±0
Gm_GulLO_1g	0±0	0±0	0.01±0.01	0±0	0±0
Gm_GulLO_3	1.2±0.34	0.85±0.15	0.62±0.12*	0.74±0.12*	0.66±0.02*
Gm_GalUR_1	0.04±0.08	0.92±0.2	2.71±0.47*	4.16±0.82*	4.1±0.24*
Gm_GalUR_2	0.2±0.02	0.17±0.05	0.68±0.11*	1.45±0.25*	0.9±0.26*
Gm_GalUR_3	0.03±0.04	0±0	0±0	0±0	0±0
Gm_GalUR_4	3.2±0.69	2.49±0.11	2.55±0.32	2.68±0.35	2.41±0.44
Gm_GalUR_5	0.2±0.03	0.65±0.08	4.67±1.18*	19.72±1.09*	19.65±2.03*
Gm_MIOX_1a	0.14±0.02	0.42±0.2	3.97±0.75*	4.31±1.11*	2.58±0.14*
Gm_MIOX_1b	0.97±0.34	1.14±0.29	1±0.24	0.44±0.07	0.59±0.04
Gm_MIOX_2a	0.16±0.02	0.01±0*	0±0*	0±0*	0±0*
Gm_MIOX_2b	3.5±1.02	3.94±0.63	4.22±1.09	2.4±0.16	1.64±0.21*
Gm_MIOX_3a	0±0	0±0	0±0	0.02±0.03	0±0
Gm_MIOX_3b	0.02±0.03	0±0.01	0±0	0.02±0.03	0.02±0

	PRJNA395215				
	1mlpa genotype				
	**Stage 1**	**Stage 2**	**Stage 3**	**Stage 4**	**Stage 5**
Gm_GMP_1a	23.33±3.07	29.75±1.51*	34.51±1.2*	26.76±0.12	25.52±4.14
Gm_GMP_1b	30.81±2.35	39.77±2.39*	39.18±1.91*	28.24±0.74	17.34±3.33*
Gm_GMP_2a	10.65±0.57	10.39±0.59	10.2±2.02	8.94±0.61	9.53±0.8
Gm_GMP_2b	19.33±0.79	23.69±1.74*	23.81±2.49*	17.37±0.87	16.69±1.82
Gm_GMP_alpha_A	12.66±1.94	12.98±1.55	13.71±1.86	12.79±1.16	13.03±2.17
Gm_GMP_alpha_B	22.7±3.16	22.68±1.27	23.69±3.19	20.4±1.97	18.67±3.29
Gm_GMP_alpha_C	7.92±0.1	8.11±0.19	7.39±1.26	4.78±0.16*	4.5±0.27*
Gm_GGP_1likeA	130.56±4.77	87.31±3.9*	58.46±4.01*	34.34±2.29*	22.79±3.24*
Gm_GGP_1likeB	26.86±1.55	19.87±1.62*	14.12±1.45*	8.4±0.15*	7.21±0.44*
Gm_GGP_1a	51.78±1	114.64±3.74*	108.27±14.13*	102.6±1.89*	109.61±6.91*
Gm_GGP_1b	38.64±4.79	73.29±4.72*	72.34±11.82*	53.85±1.47	33.76±5.06
Gm_GPP_1	58.88±8.31	41.69±1.58*	38.9±7.55*	27.14±1.07*	19.87±1.26*
Gm_GPP_2	6.17±0.56	12.37±3.56	18.99±6.36*	17.07±0.89*	8.42±1.88
Gm_GPP_L	2.79±0.17	1.95±0.38*	2.11±0.52	1.19±0.1*	0.66±0.1*
Gm_GalDH_1a	20.37±1.18	26.85±1.89*	32.19±3.29*	30.98±0.7*	21.28±3.55
Gm_GalDH_1b	6.5±0.69	8.95±0.21*	10.21±1.12*	7.97±1.25	4.85±0.64
Gm_GalLDH_1a	11.54±1.25	11.81±0.71	11.26±0.6	10.5±0.3	7.65±0.39*
Gm_GalLDH_1b	4.98±0.15	5.58±0.15	5.09±0.84	3.39±0.23*	3.04±0.22*
Gm_GME_1a	33.35±3.35	78.01±5.27*	98.68±15.47*	75.13±1.96*	24.44±5.03
Gm_GME_1b	18.15±0.58	12.07±1.12*	10.44±0.95*	5.71±0.15*	5.45±1.08*
Gm_GME_2a	20.61±0.89	34.12±2.08*	34.98±4.23*	23.82±0.52	13.56±2.94*
Gm_GME_2b	21.15±1.55	22.94±1.19	23.39±3.28	13.86±0.42*	7.88±1.61*
Gm_GulLO_1a	0.01±0.01	0±0	0±0	0.01±0	0.01±0.01
Gm_GulLO_1b	0.19±0.07	0.04±0.01*	0.02±0.01*	0±0*	0±0*
Gm_GulLO_1c	0.6±0.1	0.37±0.09*	0.47±0.06	0.8±0.08	0.7±0.13

Gm_GulLO_1d	0.06±0	0.09±0.02	0.07±0.02	0.03±0.01	0.05±0.01
Gm_GulLO_1e	0.13±0.03	0.19±0.03	0.4±0.14*	0.56±0.13*	0.34±0.06
Gm_GulLO_1f	0±0	0±0	0±0	0±0.01	0±0
Gm_GulLO_1g	0±0	0±0	0±0	0±0	0±0
Gm_GulLO_3	1.33±0.12	0.9±0.2*	0.97±0.12	0.71±0.03*	0.83±0.23*
Gm_GalUR_1	0.02±0.01	0.51±0.09*	0.49±0.1*	1.09±0.19*	1.09±0.13*
Gm_GalUR_2	0.08±0.02	0.2±0.03	0.33±0.09	0.56±0.13*	1.24±0.19*
Gm_GalUR_3	0±0	0±0	0±0	0±0	0±0
Gm_GalUR_4	2.94±0.08	3.17±0.38	3.17±0.78	2.78±0.23	2.81±0.28
Gm_GalUR_5	0.08±0.02	1.17±0.13	2.86±2.31	11.28±0.46*	18.81±2.08*
Gm_MIOX_1a	0.36±0.06	0.43±0.08	0.42±0.12	0.34±0.07	0.58±0.09*
Gm_MIOX_1b	0.84±0.08	1.02±0.47	0.74±0.2	0.35±0.07	0.41±0.1
Gm_MIOX_2a	0.02±0.03	0±0	0.01±0.01	0±0	0±0
Gm_MIOX_2b	2.72±0.28	4.35±0.64	4.19±1.46	1.6±0.41	1.72±0.24
Gm_MIOX_3a	0±0	0±0	0±0	0±0	0±0
Gm_MIOX_3b	0.05±0.01	0.02±0	0±0*	0±0*	0.07±0.03
	**PRJNA395215**				
	**1MWT genotype**				
	**Stage 1**	**Stage 2**	**Stage 3**	**Stage 4**	**Stage 5**
Gm_GMP_1a	30.55±3.94	34.28±4.31	36.84±2.68	30.44±0.27	24.31±2.09
Gm_GMP_1b	53.06±4.61	39.97±2.8*	42.74±4.33*	33.79±1.53*	16.84±3.93*
Gm_GMP_2a	9.73±1	10.63±0.69	10.93±1.15	9.64±0.47	9.22±0.33
Gm_GMP_2b	20.27±1.18	22.99±4.02	25.66±1.24	20.4±1.27	16.22±2.35
Gm_GMP_alpha_A	10.3±1.46	11.34±1.75	14.1±0.62*	13.35±0.53	10.71±1.34
Gm_GMP_alpha_B	20.43±4.16	23.96±3.06	31.42±2.75*	26.3±1.14	14.72±0.63
Gm_GMP_alpha_C	6.88±0.64	7.79±0.74	7.47±0.58	6.23±0.45	4.41±0.21*
Gm_GGP_1likeA	115.9±10.13	98.54±6.39	74.05±10.34*	50.53±6.39*	20.37±2.81*
Gm_GGP_1likeB	26.97±2.3	21.56±1.49*	15.75±1.14*	10.15±0.29*	6.32±0.66*

Gm_GGP_1a	58.58±5.53	85.15±7.61*	94.47±2.05*	87.04±1.71*	101.37±7.55*
Gm_GGP_1b	44.39±6.92	48.75±3.5	59.21±7.24*	60.07±4.17*	28.99±5.26*
Gm_GPP_1	45.27±5.27	46.64±8.22	44.05±1.76	38.46±2.86	15.59±1.64*
Gm_GPP_2	4.14±0.38	5.21±0.64	11.97±2.03*	23.75±2.03*	6.59±1.67
Gm_GPP_L	3±0.42	2.47±0.48	2.53±0.18	2.1±0.26*	0.67±0.07*
Gm_GalDH_1a	17.71±2.59	21.75±5.27	26.62±0.68*	29.38±2.21*	18.42±1.63
Gm_GalDH_1b	4.59±0.33	5.7±0.95	7.81±0.65*	7.89±1.32*	4.97±1.42
Gm_GalLDH_1a	10.29±1.51	10.92±1.11	11.39±0.48	10.67±0.47	7.61±0.86*
Gm_GalLDH_1b	4.94±0.33	5.02±0.25	5.37±0.27	4.29±0.34	2.7±0.4*
Gm_GME_1a	28.86±2.21	48.47±10.64*	99.45±3.52*	88.7±4.2*	26.01±4.72
Gm_GME_1b	17.5±2.54	12.57±2.04*	12.07±0.58*	8.24±0.77*	4.98±1.48*
Gm_GME_2a	22.27±1.86	27.47±4.82	34.87±2.89*	28.33±1.68	12.61±1.88*
Gm_GME_2b	18.31±0.59	19.49±0.71	25.93±3.08*	17.95±1.85	7.1±1.22*
Gm_GulLO_1a	0.01±0.01	0±0	0±0	0±0	0±0
Gm_GulLO_1b	0.08±0.02	0.05±0.04	0.02±0.01*	0±0*	0±0*
Gm_GulLO_1c	0.57±0.09	0.45±0.01	0.67±0.15	0.79±0.09	0.51±0.1
Gm_GulLO_1d	0.14±0.05	0.1±0.02	0.04±0.02*	0.04±0.01*	0.06±0.02*
Gm_GulLO_1e	0.25±0.04	0.29±0.04	0.41±0.1	0.48±0.02*	0.33±0.12
Gm_GulLO_1f	0±0	0±0	0±0	0±0	0±0.01
Gm_GulLO_1g	0±0	0.01±0.01	0±0	0±0	0±0
Gm_GulLO_3	1.31±0.42	1.18±0.13	0.99±0.26	0.63±0.09*	0.86±0.1
Gm_GalUR_1	0.09±0.06	0.27±0.07	0.44±0.06*	0.86±0.08*	0.67±0.1*
Gm_GalUR_2	0.15±0.03	0.3±0.07	0.27±0.06	1.01±0.12*	0.64±0.15*
Gm_GalUR_3	0±0	0±0	0±0	0±0	0±0
Gm_GalUR_4	3.18±0.1	3.06±0.13	3.49±0.19	3.43±0.16	2.44±0.31*
Gm_GalUR_5	0.13±0.04	0.5±0.1	1.17±0.28	11.02±4.31*	14.89±3.9*
Gm_MIOX_1a	0.41±0.14	0.73±0.15	1.26±0.15*	3.56±0.58*	2.12±0.33*
Gm_MIOX_1b	2.25±0.23	2.62±0.11	3.12±0.83	1.05±0.16*	0.94±0.22*

Gm_MIOX_2a	0±0	0.01±0.01	0±0.01	0±0	0±0
Gm_MIOX_2b	6.32±0.94	7.49±0.57*	10.19±2.01	4.55±0.59	3.51±0.37*
Gm_MIOX_3a	0.05±0.08	0±0*	0±0*	0±0*	0±0*
Gm_MIOX_3b	0.07±0.02	0.05±0.03	0.02±0.03	0±0*	0±0*
