# Supplementary material for: Tissue- and Condition-Specific Biosynthesis of Ascorbic Acid in Glycine max L.: Insights from Genome-Wide Analyses of Pathway-Encoding Genes, Expression Profiling, and Mass Fraction Determination
Source: Int J Mol Sci. 2025 May 14;26(10):4678. doi: 10.3390/ijms26104678 (PMC12111785; doi:10.3390/ijms26104678)
Supplement: Supplementary file 1 [file ijms-26-04678-s001.zip › Suppl. Table S6.pdf]

Supplementary Table S6. Means of RPKM values ± SD (standard deviation) of AsA biosynthesis transcripts in leaves and roots of soybean under NaCl (bioprojects PRJNA432861, PRJNA246058), drought (PRJNA574626), dehydration (PRJNA259941, PRJNA246058) and submergence (bioproject PRJNA574626). Statistical analysis (one-way ANOVA followed by Bonferroni test) was applied to the respective controls. Up and downregulated genes are in green and red, respectively. Significant differences are indicated by * at $p < 0.05$.

| | **PRJNA432861** | | | | | |
|---|---|---|---|---|---|---|
| | **NaCl (Leaves)** | | | | | |
| | **Control** | **1 h** | **2 h** | **4 h** | **24 h** | **48 h** |
| *Gm_GMP_1a* | 21.15±2.4 | 39.65±1.85* | 32.65±3.35* | 16.19±0.6* | 8.3±0.44* | 10.8±0.94* |
| *Gm_GMP_1b* | 22.51±0.57 | 32.77±1.67* | 17.43±2.14* | 8.28±0.25* | 9.81±0.75* | 7.33±0.11* |
| *Gm_GMP_2a* | 1.69±0.27 | 2.38±0.24 | 3.98±0.26* | 3.34±0.3* | 1.88±0.18 | 2.29±0.43 |
| *Gm_GMP_2b* | 1.8±0.2 | 1.96±0.12 | 2.32±0.18 | 2.22±0.03 | 2.54±0.57* | 2.48±0.19* |
| *Gm_GMP_alpha_A* | 0.32±0.04 | 0.33±0.05 | 0.37±0.01 | 0.57±0.06* | 0.16±0.01* | 0.44±0.06* |
| *Gm_GMP_alpha_B* | 0.72±0.09 | 1.81±0.41* | 1.56±0.25* | 1.7±0.23* | 0.37±0.1 | 0.58±0.07 |
| *Gm_GMP_alpha_C* | 1.97±0.32 | 2±0.24 | 3.39±0.14* | 3.81±0.21* | 3.17±0.42* | 4.9±0.33* |
| *Gm_GGP_1likeA* | 26.82±0.75 | 22.36±4.01 | 16.31±2.99* | 18.93±1.56* | 23.35±0.39 | 14.06±0.72* |
| *Gm_GGP_1likeB* | 2.91±0.04 | 5.95±0.74* | 3.56±0.67 | 4.89±0.34* | 3.49±0.08 | 2.53±0.35 |
| *Gm_GGP_1a* | 165.46±21.25 | 152.72±12.48 | 96.23±9.25* | 131.17±0.89* | 225.32±13.89* | 114.16±7.75* |
| *Gm_GGP_1b* | 249.74±17.21 | 135.52±14.98* | 99.95±9.44* | 87.21±5.21* | 335.34±16.92* | 135.06±10.62* |
| *Gm_GPP_1* | 15.94±1.11 | 20.78±0.87* | 10.88±1.55* | 10.06±0.95* | 19.22±2.69 | 11.05±0.51* |
| *Gm_GPP_2* | 4.79±0.61 | 4.17±0.36 | 3.62±0.11* | 2.35±0.08* | 4.05±0.49 | 4.89±0.13 |
| *Gm_GPP_L* | 0.14±0.03 | 0.17±0.04 | 0.13±0.02 | 0.15±0.04 | 0.07±0.02 | 0.08±0.01 |
| *Gm_GalDH_1a* | 3.79±0.45 | 4.13±0.6 | 3.02±0.75 | 4.3±0.15 | 2.37±0.54* | 4.88±0.51 |
| *Gm_GalDH_1b* | 0±0 | 0.06±0.1 | 0.03±0.04 | 0±0 | 0.06±0.1 | 0.14±0.05 |
| *Gm_GalLDH_1a* | 1.15±0.11 | 1.18±0.2 | 1.27±0.12 | 1.15±0.09 | 1.92±0.09* | 3.88±0.33* |
| *Gm_GalLDH_1b* | 0.34±0.01 | 0.26±0.06 | 0.39±0.08 | 0.71±0.07 | 0.82±0.24* | 2.49±0.3* |
| *Gm_GME_1a* | 49.05±6.41 | 56.21±13.61 | 43.39±2.24 | 42.17±5.96 | 65.02±20.77 | 86.71±15.05* |
| *Gm_GME_1b* | 27.43±4.08 | 10.68±2.5* | 4.21±1.01* | 1.9±0.39* | 2.89±1.21* | 3.23±0.82* |
| *Gm_GME_2a* | 71.03±1.63 | 57.63±5.9* | 45.4±4.92* | 34.98±0.41* | 70.72±1.58 | 53.35±2.81* |
| *Gm_GME_2b* | 31.9±0.61 | 27.43±1.46* | 19.08±1.4* | 16.59±0.61* | 23.33±1.5* | 24.76±1.49* |
| *Gm_GulLO_1a* | 0.02±0.03 | 0±0.01 | 0.02±0.01 | 0±0.01 | 0±0.01 | 0.02±0.01 |
| *Gm_GulLO_1b* | 1.17±0.23 | 1.2±0.05 | 1.03±0.17 | 1.18±0.19 | 1.5±0.21 | 0.1±0.03* |
| *Gm_GulLO_1c* | 0.09±0.03 | 0.11±0.02 | 0.08±0.01 | 0.1±0.03 | 0.05±0.03 | 0.02±0.01* |
| *Gm_GulLO_1d* | 0±0 | 0±0 | 0±0 | 0±0 | 0±0 | 0±0 |
| *Gm_GulLO_1e* | 0.04±0.01 | 0.07±0.01* | 0.03±0.01 | 0.02±0.01 | 0.02±0.01 | 0±0* |
| *Gm_GulLO_1f* | 0.02±0 | 0.04±0.02 | 0.06±0.01 | 0.02±0.01 | 0.02±0.01 | 0.31±0.07* |
| *Gm_GulLO_1g* | 0.09±0.03 | 0.06±0.02 | 0.07±0.01 | 0.02±0.01* | 0.02±0.01* | 0±0.01* |
| *Gm_GulLO_3* | 0.39±0.11 | 0.45±0.07 | 0.41±0.09 | 0.29±0.09 | 0.46±0.06 | 0.41±0.06 |
| *Gm_GalUR_1* | 0.02±0 | 0.13±0.06* | 0±0 | 0.06±0.02 | 0±0 | 0.04±0.01 |
| *Gm_GalUR_2* | 1.45±0.22 | 12.95±1.81* | 20.22±3.52* | 3.84±0.58 | 7.86±1.48* | 3.22±0.5 |
| *Gm_GalUR_3* | 0±0 | 0.01±0.02 | 0±0 | 0±0 | 0±0 | 0±0 |
| *Gm_GalUR_4* | 0.45±0.09 | 0.44±0.01 | 0.74±0.11 | 2.2±0.38* | 1.31±0.1* | 3.2±0.26* |
| *Gm_GalUR_5* | 0.29±0.03 | 0.22±0.14 | 0.94±0.16 | 1.86±0.4* | 1.7±0.54* | 2.94±0.54* |
| *Gm_MIOX_1a* | 0.08±0.02 | 0.06±0.01 | 0.13±0.08 | 0.47±0.1 | 1.24±0.34 | 30.39±4.74* |
| *Gm_MIOX_1b* | 2.48±0.65 | 0.89±0.18 | 2.51±0.45 | 1.87±0.23 | 18.68±5.85* | 81.01±10.3* |
| *Gm_MIOX_2a* | 4.08±0.42 | 7.2±1.56* | 2.41±0.3 | 0.98±0.07* | 1.78±0.77* | 2.72±0.27 |
| *Gm_MIOX_2b* | 1.38±0.61 | 0.84±0.07 | 1.2±0.27 | 0.38±0.06* | 0.91±0.1 | 1.72±0.24 |
| *Gm_MIOX_3a* | 0.12±0.03 | 0.14±0.05 | 0.1±0.03 | 0.13±0.03 | 0.05±0.01 | 0.03±0.01* |
| *Gm_MIOX_3b* | 0±0 | 0±0 | 0±0 | 0±0 | 0±0 | 0.01±0.01 |

| | PRJNA259941 | | | | | | | |
|---|---|---|---|---|---|---|---|---|
| | Dehydration (Leaves) | | | | | | | |
| | **Benning** (drought sensitive) | | | | **PI416937** (drought tolerant) | | | |
| | **Control** | **6 h** | **12 h** | **24 h** | **Control** | **6 h** | **12 h** | **24 h** |
| Gm_GMP_1a | 53.28±1.37 | 22.02±4.63* | 27.27±3.59* | 21.43±4.55* | 62.32±7.32 | 23.43±5.1* | 13.73±2.26* | 24.57±1* |
| Gm_GMP_1b | 22.51±3.28 | 7.34±0.47* | 9.55±1.04* | 18.79±3.74 | 33.57±5.77 | 8.03±0.27* | 9.49±1.34* | 29.88±3.54 |
| Gm_GMP_2a | 5.51±0.36 | 6.46±0.8 | 8.58±2* | 4.53±1.13 | 5.75±0.49 | 8.16±0.76* | 6.59±0.84 | 5.95±0.9 |
| Gm_GMP_2b | 4.56±0.31 | 3.59±1.05 | 5.54±0.77 | 4.55±0.59 | 5.42±0.57 | 4.68±0.53 | 5.31±0.69 | 5.78±1.7 |
| Gm_GMP_alpha_A | 2.84±0.83 | 0.57±0.18* | 0.52±0.12* | 0.18±0.04* | 6.86±1.41 | 1.5±0.24* | 0.83±0.12* | 0.16±0.02* |
| Gm_GMP_alpha_B | 6.83±0.79 | 3.1±1.44* | 2.82±0.33* | 0.25±0.04* | 9.3±2.47 | 6.9±1.09 | 1.58±0.14* | 0.46±0.13* |
| Gm_GMP_alpha_C | 7.99±1.22 | 9.4±0.79 | 8.52±1.23 | 3.46±0.5* | 7.58±0.44 | 10.46±1.19* | 9.16±1.08 | 5.32±1.16 |
| Gm_GGP_1likeA | 25.91±2.4 | 17.11±0.75 | 20.48±0.77 | 33.18±8.51 | 35.45±2.35 | 17.28±1.37* | 23.83±2.77* | 23.07±5.09* |
| Gm_GGP_1likeB | 12.68±1.68 | 15.27±1.47 | 14.78±3.27 | 7.81±2.4 | 18.01±1.79 | 17.06±1.17 | 14.57±2.07 | 6.33±0.8* |
| Gm_GGP_1a | 271.68±42.86 | 21.55±4.82* | 30.71±5.3* | 31.77±4.37* | 450.19±48.94 | 56.6±3.01* | 33.22±4.12* | 50.97±11.64* |
| Gm_GGP_1b | 93.08±7.36 | 32.46±3.12* | 77.84±13.22 | 135.81±11.35* | 126.84±4.14 | 53.44±4.29* | 64.66±5.86* | 106.27±16.57 |
| Gm_GPP_1 | 10.79±2.89 | 0.65±0.11* | 1.77±0.67* | 0.75±0.1* | 15.5±5.49 | 3.04±0.33* | 1.59±0.19* | 1.48±0.51* |
| Gm_GPP_2 | 5.16±1.41 | 1.14±0.53* | 0.57±0.05* | 0.17±0.02* | 2.7±0.48 | 1.62±0.33* | 0.5±0.27* | 0.7±0.16* |
| Gm_GPP_L | 1.82±0.52 | 1.1±0.25 | 7.29±9.95* | 0.18±0.04 | 1.82±0.47 | 7.95±1.31* | 1.74±0.36 | 1.35±0.59 |
| Gm_GalDH_1a | 7.55±0.89 | 2.32±0.26* | 2.35±0.55* | 1.27±0.28* | 12.81±3.24 | 3.74±0.68* | 4.02±1.17* | 1.49±0.31* |
| Gm_GalDH_1b | 0.28±0.06 | 0.26±0.09 | 0.37±0.09 | 0.12±0.03 | 0.64±0.3 | 0.69±0.61 | 0.16±0.04 | 0.59±0.38 |
| Gm_GalLDH_1a | 3.41±0.75 | 0.82±0.09* | 1.15±0.16* | 0.13±0.03* | 4.97±0.44 | 1.78±0.19* | 1.64±0.48* | 0.25±0.08* |
| Gm_GalLDH_1b | 1.92±0.55 | 1.8±0.21 | 1.5±0.01 | 1.28±0.36 | 2.45±0.18 | 2.94±0.26 | 2.06±0.56 | 0.93±0.18* |
| Gm_GME_1a | 18.2±3.88 | 3.55±0.55* | 2.74±0.85* | 4.45±0.67* | 39.53±2.78 | 4.72±0.63* | 5.54±0.71* | 2.62±0.49* |
| Gm_GME_1b | 11.19±1.91 | 2.5±0.48* | 1.08±0.46* | 2.49±0.34* | 23.37±3.02 | 0.71±0.16* | 1.42±0.31* | 2.16±0.32* |
| Gm_GME_2a | 87.57±7.5 | 10.71±2.71* | 22.04±0.92* | 13.39±2.13* | 142.97±7 | 14.2±3.2* | 24.24±4.34* | 17.01±2.79* |
| Gm_GME_2b | 31.54±1.86 | 26.92±1.87 | 27.64±4.19 | 17.01±1.87* | 39.19±2.31 | 41.39±2.81 | 27.68±0.6* | 19.13±3.78* |
| Gm_GulLO_1a | 0±0 | 0.02±0 | 0.08±0.01* | 0.12±0.02* | 0.03±0.02 | 0.07±0.05 | 0.44±0.29* | 0.04±0.02 |
| Gm_GulLO_1b | 0.46±0.19 | 0.05±0.02* | 0.04±0.02* | 0±0* | 1.25±0.31 | 0.14±0.04* | 0.04±0.02* | 0±0* |
| Gm_GulLO_1c | 0.52±0.1 | 0.08±0.05* | 0.06±0.05* | 0.01±0.01* | 0.86±0.14 | 0.21±0.1* | 0.04±0.02* | 0.05±0.04* |
| Gm_GulLO_1d | 0±0 | 0±0 | 0±0 | 0±0 | 0±0 | 0±0 | 0±0 | 0±0 |
| Gm_GulLO_1e | 0.04±0.02 | 0.01±0.01* | 0.01±0.01* | 0.02±0 | 0.47±0.07 | 0.27±0.13* | 0.1±0.04* | 0.03±0.01* |
| Gm_GulLO_1f | 0.48±0.11 | 4.87±0.58* | 8.34±0.75* | 61.4±6.51* | 0.69±0.06 | 2.18±0.24 | 6.65±0.9* | 43.65±6.83* |
| Gm_GulLO_1g | 1.92±0.42 | 1.53±0.24 | 0.7±0.19* | 0.2±0.06* | 4.12±1.11 | 2.65±0.73 | 2.22±0.42* | 1.05±0.52* |
| Gm_GulLO_3 | 0.96±0.39 | 0.17±0.03* | 0.23±0.2* | 0.2±0.01* | 1.84±0.28 | 0.23±0.13* | 0.64±0.17* | 0.06±0.02* |
| Gm_GalUR_1 | 0.5±0.14 | 4.49±1.46 | 27.39±5.69* | 0.58±0.15 | 0.33±0.11 | 7.84±1.6* | 4.03±1.55* | 4.32±1.04* |
| Gm_GalUR_2 | 57.52±10.45 | 135.6±12.23* | 388.44±70.61* | 77.02±11 | 15.79±2.66 | 336.19±4.67* | 78.7±3.02* | 93.2±15.81* |
| Gm_GalUR_3 | 0±0 | 0±0 | 0±0 | 0±0 | 0.02±0.03 | 0.02±0.03 | 0.01±0.02 | 0±0 |
| Gm_GalUR_4 | 2.59±0.38 | 18.64±1.4* | 13.36±2.36* | 13.37±1.12* | 3.28±0.43 | 19.19±2.36* | 16.32±3.2* | 21.79±4.32* |
| Gm_GalUR_5 | 4.24±0.81 | 12.83±2.57 | 52.03±14.25* | 9.41±2.28 | 2.31±0.46 | 19.9±2.75* | 16.93±2.81* | 15.96±7.24* |
| Gm_MIOX_1a | 2.83±0.8 | 2.93±0.5 | 1.33±0.61 | 21.56±4.04* | 2.51±0.48 | 1.48±0.22 | 4.85±1.82 | 6.42±1.28* |
| Gm_MIOX_1b | 6.68±0.87 | 14.82±1.66 | 6.07±2.31 | 62.03±6.86* | 2.45±0.27 | 4.18±1.08 | 12.31±2.87 | 45.4±7.7* |
| Gm_MIOX_2a | 6.54±1.05 | 0.4±0.08* | 0.4±0.14* | 0.1±0.03* | 24.75±10.09 | 1.43±0.28* | 0.34±0.19* | 0.05±0.01* |
| Gm_MIOX_2b | 198.85±17.26 | 16.73±4.59* | 4.27±0.44* | 6.82±1.64* | 301.77±32.69 | 44.83±9.19* | 15.1±5.19* | 10.78±3.12* |
| Gm_MIOX_3a | 0.05±0.02 | 0.67±0.21 | 0.47±0.6 | 43.8±6.21* | 0.14±0.02 | 0.15±0.08 | 1.71±0.33 | 63.45±15.5* |
| Gm_MIOX_3b | 0±0 | 0.08±0.03* | 0.03±0.01 | 0.07±0.02* | 0±0 | 0±0 | 0.15±0.15 | 0.03±0.06 |

| | PRJNA432861 | | | | | |
|---|---|---|---|---|---|---|
| | NaCl (Roots) | | | | | |
| | **Control** | **1 h** | **2 h** | **4 h** | **24 h** | **48 h** |
| *Gm_GMP_1a* | 31.85±4.59 | 30.18±2.15 | 25.12±2.85* | 14.69±0.95* | 9.31±1.81* | 16.93±2.28* |
| *Gm_GMP_1b* | 21.53±2.23 | 24.36±3.29 | 21.76±0.86 | 11.51±0.99* | 9.27±1.4* | 14.35±2.15* |
| *Gm_GMP_2a* | 6.72±0.6 | 6.11±0.5 | 6.61±1.89 | 4.11±0.28* | 4.43±0.29* | 7.19±0.61 |
| *Gm_GMP_2b* | 5.53±0.84 | 4.67±0.42 | 4.3±1.04 | 3.31±0.5* | 3.38±0.05* | 4.77±0.34 |
| *Gm_GMP_alpha_A* | 1.29±0.24 | 1.55±0.07 | 1.32±0.34 | 1.74±0.28 | 1.54±0.31 | 2.11±0.23* |
| *Gm_GMP_alpha_B* | 1.36±0.56 | 1.39±0.21 | 0.94±0.13 | 0.85±0.22 | 0.59±0.1* | 1±0.3 |
| *Gm_GMP_alpha_C* | 6.11±0.27 | 6.21±0.24 | 5.41±0.63 | 6.12±0.46 | 7.52±0.98 | 7.55±0.59 |
| *Gm_GGP_1likeA* | 12.04±1.26 | 8.31±1.12* | 10.97±2.14 | 12.73±1.07 | 7.78±0.46* | 7.89±0.86* |
| *Gm_GGP_1likeB* | 2.43±0.06 | 3.05±0.45 | 4.1±0.64* | 4.24±0.21* | 1.83±0.03 | 2.42±0.37 |
| *Gm_GGP_1a* | 4.16±0.41 | 3.78±0.83 | 4.52±1.07 | 6.85±0.57* | 11.13±1.63* | 9.6±0.94* |
| *Gm_GGP_1b* | 6.19±0.23 | 5.86±0.99 | 9.86±1.88 | 14.9±0.93* | 16.69±4.01* | 10.32±1.34 |
| *Gm_GPP_1* | 5.37±1.27 | 5.51±0.09 | 4.4±1.04 | 3.41±0.2 | 5.4±0.63 | 5.3±0.9 |
| *Gm_GPP_2* | 1.68±0.51 | 1.76±0.02 | 1.34±0.25 | 0.72±0.06* | 0.86±0.1* | 1.26±0.32 |
| *Gm_GPP_L* | 0.38±0.13 | 0.2±0.04* | 0.17±0.03* | 0.12±0.03* | 0.16±0.06* | 0.25±0.08 |
| *Gm_GalDH_1a* | 3.81±0.43 | 3.9±0.43 | 2.96±0.82 | 1.75±0.27* | 2.17±0.19* | 3.22±0.64 |
| *Gm_GalDH_1b* | 0.05±0.09 | 0.14±0.03 | 0.11±0.06 | 0.03±0.05 | 0.05±0.09 | 0.12±0.06 |
| *Gm_GalLDH_1a* | 3.16±0.45 | 3.45±0.14 | 2.65±0.64 | 1.83±0.23* | 2.31±0.27 | 3.21±0.61 |
| *Gm_GalLDH_1b* | 1.49±0.15 | 1.24±0.31 | 0.7±0.17* | 0.9±0.14 | 1.96±0.29 | 1.96±0.37 |
| *Gm_GME_1a* | 1.02±0.32 | 0.19±0.05* | 0.14±0.08* | 0.36±0.43* | 0.42±0.23 | 0.43±0.07 |
| *Gm_GME_1b* | 0.36±0.06 | 0.87±0.1 | 4.34±1.27* | 9.25±3.67* | 0.25±0.09 | 0.23±0.05 |
| *Gm_GME_2a* | 21.23±2.89 | 15.4±0.89 | 21.47±4.02 | 20.36±1.62 | 25.45±3.89 | 24.48±0.38 |
| *Gm_GME_2b* | 12.04±2.13 | 7.11±0.44* | 6.52±1.9* | 9.08±0.81 | 10±1.08 | 10.6±0.05 |
| *Gm_GulLO_1a* | 72.07±8.38 | 85.93±12.7 | 126.17±11.01* | 110.12±5.05* | 112.24±2.55* | 77.72±14.37 |
| *Gm_GulLO_1b* | 6.94±1.43 | 13.26±0.71* | 10.85±0.62* | 5.99±0.65 | 1.79±0.33* | 2.92±0.53* |
| *Gm_GulLO_1c* | 0.64±0.08 | 0.91±0.15 | 0.76±0.16 | 0.9±0.17 | 0.56±0.1 | 0.5±0.07 |
| *Gm_GulLO_1d* | 0.02±0.01 | 0.01±0.01 | 0.02±0.01 | 0±0.01 | 0.03±0 | 0.02±0.01 |
| *Gm_GulLO_1e* | 0±0 | 0±0.01 | 0.02±0.01* | 0±0 | 0.02±0.01* | 0.01±0.01 |
| *Gm_GulLO_1f* | 3.27±0.45 | 1.71±0.21 | 3.33±0.68 | 7.92±0.71 | 21.21±6.47* | 16.03±1.53* |
| *Gm_GulLO_1g* | 0±0 | 0.03±0.01* | 0.01±0.01 | 0±0 | 0±0 | 0.03±0* |
| *Gm_GulLO_3* | 2.53±0.43 | 1.32±0.07* | 1.14±0.25* | 1.34±0.11* | 1.3±0.06* | 1.19±0.17* |
| *Gm_GalUR_1* | 30.12±6.25 | 15.49±2.06 | 53.87±3.94* | 94.42±2.89* | 77.53±11.02* | 61.37±9.28* |
| *Gm_GalUR_2* | 99.29±12.04 | 342.57±24.17* | 283.36±15.37* | 232.46±10.38* | 237.78±33.09* | 178.76±26.8* |
| *Gm_GalUR_3* | 0±0 | 0±0 | 0±0 | 0±0 | 0±0 | 0±0 |
| *Gm_GalUR_4* | 5.13±0.47 | 4.14±0.1 | 4.23±0.36 | 7.44±0.3* | 8.9±0.61* | 7.05±0.57* |
| *Gm_GalUR_5* | 125.07±9 | 90.71±1.5 | 151.9±12.68 | 273.11±22.89* | 378.64±26.83* | 269.22±53.69* |
| *Gm_MIOX_1a* | 17.37±3.4 | 27.18±2.69* | 40.21±3.7* | 39.36±2.67* | 19.92±3.01 | 10.02±2.49 |
| *Gm_MIOX_1b* | 83.27±3.8 | 43.54±6.81* | 24.1±4.03* | 50.48±4.02* | 55.66±9.69* | 48.8±2.57* |
| *Gm_MIOX_2a* | 0.35±0.04 | 0.32±0.1 | 0.05±0.01* | 0.02±0* | 0±0* | 0±0* |
| *Gm_MIOX_2b* | 33.01±2.43 | 64.16±10.68* | 97.25±7.79* | 57.65±12.58* | 10.01±2.13* | 16.95±1.45 |
| *Gm_MIOX_3a* | 0.93±0.15 | 0.45±0.61 | 0.91±0.16 | 2.13±1.04 | 32.24±4.33* | 30.29±6.34* |
| *Gm_MIOX_3b* | 0.01±0.02 | 0.02±0.01 | 0.01±0.01 | 0.01±0.02 | 0±0 | 0.03±0.01 |

| | PRJNA246058 | | | |
|---|---|---|---|---|
| | Control | NaCl (Roots) | | |
| | | 1 h | 6 h | 12 h |
| *Gm_GMP_1a* | 38.27±2.69 | 31.46±0.81 | 71.52±31.7 | 35.96±4.85 |
| *Gm_GMP_1b* | 54.75±3.95 | 47.81±3.72 | 79.87±10.33* | 61.08±8.94 |
| *Gm_GMP_2a* | 21.53±1.77 | 25.34±4.26 | 18.4±2.94 | 14.04±0.91* |
| *Gm_GMP_2b* | 27.25±2.76 | 22.34±1.71 | 15.72±1.91* | 15.19±2.83* |
| *Gm_GMP_alpha_A* | 13.09±2.1 | 10.21±2.93 | 12.99±2.45 | 13.06±0.81 |
| *Gm_GMP_alpha_B* | 8.04±1.71 | 9.31±0.82 | 8.24±1.2 | 7.82±1.04 |
| *Gm_GMP_alpha_C* | 8±1.19 | 11.97±1.55 | 11.72±3.07 | 11.43±1.39 |
| *Gm_GGP_1likeA* | 33.75±1.55 | 31.9±2.34 | 25.47±1.01* | 27.92±4.1 |
| *Gm_GGP_1likeB* | 8.26±1.19 | 11.91±0.56* | 9.27±0.86 | 12±2.11* |
| *Gm_GGP_1a* | 80.89±29.39 | 53.75±0.37 | 33.88±7.8* | 31.15±3.86* |
| *Gm_GGP_1b* | 45.58±10.5 | 49.86±2.88 | 38.86±1.5 | 37.16±3.49 |
| *Gm_GPP_1* | 11.42±3.05 | 12.78±2.05 | 7.39±1.48 | 6.5±0.94 |
| *Gm_GPP_2* | 5.86±1.19 | 4.3±0.67 | 2.16±0.15* | 2.03±0.17* |
| *Gm_GPP_L* | 4.04±0.59 | 2.42±0.71 | 2.79±0.93 | 3.1±0.37 |
| *Gm_GalDH_1a* | 10.81±2.53 | 10.74±0.95 | 5.65±1.38* | 6.71±0.93* |
| *Gm_GalDH_1b* | 0.55±0.37 | 0.86±0.19 | 0.25±0.16 | 0±0* |
| *Gm_GalLDH_1a* | 6.85±1.01 | 7.23±0.09 | 4.58±1.12* | 4.97±0.51 |
| *Gm_GalLDH_1b* | 3.69±0.5 | 3.41±0.22 | 2.74±0.17* | 2.96±0.13 |
| *Gm_GME_1a* | 1.6±0.17 | 0.52±0.18 | 1.75±1.16 | 2.98±0.46 |
| *Gm_GME_1b* | 0.46±0.04 | 0.37±0.07 | 0.44±0.13 | 0.68±0.09* |
| *Gm_GME_2a* | 19.81±4.42 | 26.53±3.98 | 11.73±2.8 | 13.84±0.67 |
| *Gm_GME_2b* | 12.18±2.03 | 12.96±1.5 | 7.81±0.67* | 7.99±1.35* |
| *Gm_GulLO_1a* | 3.52±1.03 | 14.22±2.72* | 24.53±3* | 26.25±2.61* |
| *Gm_GulLO_1b* | 8.39±1.45 | 13.1±3.76 | 17.42±2.07* | 12.75±1.83 |
| *Gm_GulLO_1c* | 5.08±0.96 | 5.22±1.02 | 4.71±1.32 | 5.26±1.36 |
| *Gm_GulLO_1d* | 0.05±0.01 | 0.17±0.22 | 0±0 | 0.02±0.04 |
| *Gm_GulLO_1e* | 0.07±0.12 | 0±0.01 | 0±0 | 0.03±0.01 |
| *Gm_GulLO_1f* | 1±0.17 | 1.14±0.6 | 3.65±0.97 | 6.26±2.35* |
| *Gm_GulLO_1g* | 0.02±0.03 | 0.02±0.03 | 0.01±0.01 | 0±0 |
| *Gm_GulLO_3* | 11.69±2.87 | 7.52±0.45 | 4.58±0.59* | 10.27±3.59 |
| *Gm_GalUR_1* | 11.88±5.11 | 9.08±0.89 | 38.31±7.9* | 62.62±18.35* |
| *Gm_GalUR_2* | 64.01±19.1 | 133.84±12.46* | 158.9±28.99* | 324.25±19.61* |
| *Gm_GalUR_3* | 0±0 | 0±0 | 0±0 | 0.03±0.04 |
| *Gm_GalUR_4* | 7.74±0.72 | 8.57±1.15 | 9.93±2.03 | 11.51±1.2* |
| *Gm_GalUR_5* | 61.48±20.92 | 102.61±7.4* | 133.2±25.12* | 291.8±37.11* |
| *Gm_MIOX_1a* | 24.45±4.78 | 52.85±10.45* | 13.4±2.49 | 2.39±0.83* |
| *Gm_MIOX_1b* | 5.38±0.63 | 10.88±4.76 | 2.6±0.54 | 0.62±0.47 |
| *Gm_MIOX_2a* | 0.02±0.04 | 0±0 | 0.07±0.05 | 0.03±0.06 |
| *Gm_MIOX_2b* | 7.47±0.58 | 64.04±16.12* | 12.11±1.84 | 14.71±1.99 |
| *Gm_MIOX_3a* | 0.02±0.04 | 0.01±0.03 | 0.56±0.1* | 0.17±0.07 |
| *Gm_MIOX_3b* | 0±0 | 0±0 | 0.01±0.02 | 0.03±0.06 |

| | PRJNA246058 | | | |
|---|---|---|---|---|
| | Control | Dehydration (Roots) | | |
| | | 1 h | 6 h | 12 h |
| *Gm_GMP_1a* | 38.27±2.69 | 30.52±1.37* | 33.92±3.3 | 29.18±2.42* |
| *Gm_GMP_1b* | 54.75±3.95 | 48.02±7.1 | 45.73±11.53 | 44.99±1.8 |
| *Gm_GMP_2a* | 21.53±1.77 | 21.1±3.07 | 24.49±1.75 | 25.19±4.43 |
| *Gm_GMP_2b* | 27.25±2.76 | 20.43±1.2* | 24.88±1.85 | 23.22±2.03 |
| *Gm_GMP_alpha_A* | 13.09±2.1 | 6.82±0.76* | 15.87±1.97 | 9.78±1.86 |
| *Gm_GMP_alpha_B* | 8.04±1.71 | 5.59±1.34 | 8.17±0.63 | 5.52±0.66 |
| *Gm_GMP_alpha_C* | 8±1.19 | 10.28±1.75 | 10.02±0.88 | 9.72±1.37 |
| *Gm_GGP_1likeA* | 33.75±1.55 | 30.45±2.57 | 31.8±4.67 | 29.76±5.21 |
| *Gm_GGP_1likeB* | 8.26±1.19 | 11.17±2.2 | 10.54±1.33 | 10.91±1.48 |
| *Gm_GGP_1a* | 80.89±9.39 | 69.75±19.62 | 41.29±3.12 | 39.08±3.66 |
| *Gm_GGP_1b* | 45.58±10.5 | 35.74±12.28 | 22.22±6.4* | 21.12±4.42* |
| *Gm_GPP_1* | 11.42±3.05 | 11.71±2.57 | 11.5±2.25 | 12.85±1.31 |
| *Gm_GPP_2* | 5.86±1.19 | 3.35±0.67* | 5.98±0.92 | 5.6±0.78 |
| *Gm_GPP_L* | 4.04±0.59 | 2.51±0.36* | 3.15±0.39 | 2.83±0.69 |
| *Gm_GalDH_1a* | 10.81±2.53 | 13.19±1.49 | 12.37±0.23 | 10.77±1.21 |
| *Gm_GalDH_1b* | 0.55±0.37 | 0.55±0.18 | 0.11±0.19 | 0.21±0.37 |
| *Gm_GalLDH_1a* | 6.85±1.01 | 6.87±0.48 | 7.3±0.6 | 7.59±0.32 |
| *Gm_GalLDH_1b* | 3.69±0.5 | 3.87±0.33 | 4.05±0.27 | 2.74±0.24* |
| *Gm_GME_1a* | 1.6±0.17 | 1.35±0.49 | 1.06±0.16 | 0.51±0.11* |
| *Gm_GME_1b* | 0.46±0.04 | 0.44±0.06 | 0.54±0.1 | 0.52±0.02 |
| *Gm_GME_2a* | 19.81±4.42 | 32.2±4.61* | 15.77±1.33 | 19.42±2.26 |
| *Gm_GME_2b* | 12.18±2.03 | 8.64±1.52 | 11.06±1.96 | 10.69±2.35 |
| *Gm_GulLO_1a* | 3.52±1.03 | 8.91±1.31* | 3.63±0.89 | 2.55±0.43 |
| *Gm_GulLO_1b* | 8.39±1.45 | 8.07±0.71 | 9.14±1.66 | 6.39±2.18 |
| *Gm_GulLO_1c* | 5.08±0.96 | 4.26±0.18 | 5.7±0.66 | 4.86±1.05 |
| *Gm_GulLO_1d* | 0.05±0.01 | 0.02±0.04 | 0.02±0.03 | 0.02±0.03 |
| *Gm_GulLO_1e* | 0.07±0.12 | 0.03±0.04 | 0.02±0.03 | 0±0 |
| *Gm_GulLO_1f* | 1±0.17 | 0.73±0.27 | 0.36±0.14* | 0.71±0.12 |
| *Gm_GulLO_1g* | 0.02±0.03 | 0.07±0.02* | 0±0 | 0.12±0.02* |
| *Gm_GulLO_3* | 11.69±2.87 | 5.11±1.2* | 7.87±2.1 | 11.05±1.27 |
| *Gm_GalUR_1* | 11.88±5.11 | 7.36±3.05 | 23.74±8.22 | 27.66±4.42* |
| *Gm_GalUR_2* | 64.01±19.1 | 82.6±7.23 | 88.06±5.26 | 135.83±9.56* |
| *Gm_GalUR_3* | 0±0 | 0±0 | 0±0 | 0±0 |
| *Gm_GalUR_4* | 7.74±0.72 | 6.22±0.91 | 7.86±0.82 | 8.06±1.01 |
| *Gm_GalUR_5* | 61.48±20.92 | 58.7±17.02 | 141.63±12.33* | 119.03±22.51* |
| *Gm_MIOX_1a* | 24.45±4.78 | 41.9±6.74 | 21.65±3.65 | 27.61±11.54 |
| *Gm_MIOX_1b* | 5.38±0.63 | 6.17±0.81 | 1.74±0.61* | 1.32±0.55* |
| *Gm_MIOX_2a* | 0.02±0.04 | 0±0 | 0.09±0.03 | 0±0 |
| *Gm_MIOX_2b* | 7.47±0.58 | 55.47±8.15* | 20.14±2.81* | 28.55±3.13* |
| *Gm_MIOX_3a* | 0.02±0.04 | 0.1±0.17 | 0.05±0.08 | 0±0 |
| *Gm_MIOX_3b* | 0±0 | 0±0 | 0±0 | 0±0 |

| | PRJNA574626 (Drought) | | | | | | | |
|---|---|---|---|---|---|---|---|---|
| | Leaves | | | | Roots | | | |
| | **Control** | **5 D** | **6 D** | **REC** | **Control** | **5 D** | **6 D** | **REC** |
| *Gm_GMP_1a* | 9.45±1.22 | 7.74±0.86 | 8.02±0.69 | 23.89±1.88* | 55.14±11.43 | 20.13±1.81* | 20.67±1.53* | 84.85±9.84* |
| *Gm_GMP_1b* | 6.12±0.43 | 14.23±2.99* | 4.37±0.4 | 68.13±4.93* | 31.1±1.15 | 19.2±0.75* | 17.46±0.17* | 46.96±5.38* |
| *Gm_GMP_2a* | 3.15±0.31 | 3.72±0.97 | 3.01±0.08 | 3.14±0.07 | 10.51±1.54 | 5.92±0.46 | 6.49±0.49 | 10.57±3.74 |
| *Gm_GMP_2b* | 4.04±0.34 | 4.07±0.69 | 3.91±0.31 | 4.49±0.66 | 14.38±0.85 | 6.92±0.84* | 7.92±0.37* | 13.3±3.46 |
| *Gm_GMP_alpha_A* | 0.57±0.04 | 0.64±0.26 | 0.72±0.07 | 1.92±0.32* | 6.51±0.95 | 2.24±0.22* | 2.64±0.18* | 5.69±1.15 |
| *Gm_GMP_alpha_B* | 0.79±0.11 | 1.53±0.25* | 0.83±0.11 | 4.97±0.17* | 3.72±0.37 | 1.65±0.23* | 1.9±0.12* | 6.47±0.93* |
| *Gm_GMP_alpha_C* | 4.45±1.2 | 3.44±0.57 | 5.59±0.69 | 3.17±0.69 | 5.23±0.62 | 4.67±0.28 | 5.38±0.53 | 6.77±1.06 |
| *Gm_GGP_1likeA* | 10.15±1.74 | 10.73±2.61 | 9.19±0.72 | 19.73±1.7* | 10.59±1.54 | 7.1±1.05 | 10.07±0.32 | 10.62±2.62 |
| *Gm_GGP_1likeB* | 6.89±0.95 | 5.99±1.26 | 7.43±0.7 | 8.19±0.83 | 4.05±0.41 | 5.05±0.81 | 10.46±0.71* | 5.32±1.97 |
| *Gm_GGP_1a* | 84.65±5.4 | 209.94±303.61 | 26.48±2.87 | 252.94±2.98 | 35.52±2.16 | 18.11±1.57* | 10.94±0.61* | 16.34±2.07* |
| *Gm_GGP_1b* | 108.36±10.89 | 79.68±3.53* | 58.39±5.87* | 119.59±4.49 | 31.78±2.51 | 28.93±2.95 | 23.23±1.8* | 19.39±1.39* |
| *Gm_GPP_1* | 7.65±0.56 | 6.12±1.31 | 4.65±0.25 | 20.65±4.13* | 7.57±0.46 | 5.46±0.57 | 5.66±0.3 | 13.11±4.12* |
| *Gm_GPP_2* | 2.79±0.84 | 2.56±0.88 | 1.74±0.07 | 10.14±1.87* | 2.45±0.26 | 1.42±0.21 | 1.33±0.37* | 2±0.74 |
| *Gm_GPP_L* | 0.1±0.05 | 0.09±0.02 | 0.03±0.01* | 0.06±0.01 | 0.33±0.05 | 0.19±0.04* | 0.23±0.08 | 0.17±0.04* |
| *Gm_GalDH_1a* | 1.98±0.28 | 2.04±0.4 | 1.72±0.39 | 3.66±0.56* | 5.14±0.82 | 2.85±0.23 | 3.29±0.17 | 7.59±2.35 |
| *Gm_GalDH_1b* | 0.02±0.03 | 0.02±0.03 | 0.14±0.04* | 0.19±0.03* | 0.05±0.08 | 0±0 | 0.11±0.07 | 0±0 |
| *Gm_GalLDH_1a* | 2±0.19 | 1.68±0.2 | 1.71±0.26 | 2.73±0.23* | 3.98±0.1 | 2.9±0.38 | 3.52±0.08 | 5.66±1.21* |
| *Gm_GalLDH_1b* | 2.72±0.32 | 1.87±0.29* | 2.85±0.24 | 2.1±0.16 | 1.74±0.05 | 2.17±0.35 | 2.97±0.17* | 1.84±0.73 |
| *Gm_GME_1a* | 4.97±0.8 | 3.57±0.78 | 3.68±0.54 | 7.38±0.7* | 0.33±0.06 | 0.13±0.01* | 0.62±0.09* | 0.25±0.11 |
| *Gm_GME_1b* | 0.24±0.05 | 0.3±0.07 | 0.25±0.04 | 0.32±0.06 | 0.07±0.01 | 0.04±0 | 0.05±0.01 | 0.08±0.06 |
| *Gm_GME_2a* | 29.55±2.02 | 23.9±8.12 | 25.37±3.75 | 38.2±0.9 | 11.81±0.99 | 20.26±1.48* | 27.95±1.6* | 9.81±2.55 |
| *Gm_GME_2b* | 11.16±0.47 | 10.94±1.47 | 10.44±1.02 | 15.4±1.01* | 10.75±1.06 | 10.14±1.1 | 13.47±2.55 | 8.65±0.64 |
| *Gm_GulLO_1a* | 0±0 | 0.6±1.03 | 0±0 | 0±0 | 11.18±1.33 | 0.38±0.05* | 0.68±0.06* | 10.81±5.57 |
| *Gm_GulLO_1b* | 0.15±0.03 | 0.21±0.1 | 0.06±0.02 | 0.42±0.1* | 9.43±0.85 | 0.7±0.08* | 1.26±0.13* | 10.3±2.06 |
| *Gm_GulLO_1c* | 0.14±0.05 | 0.13±0.15 | 0.26±0.02 | 0.04±0.01 | 2.32±0.32 | 6.92±1.38* | 7.57±0.13* | 2.27±0.49 |
| *Gm_GulLO_1d* | 0.01±0 | 0.01±0 | 0.01±0.01 | 0±0 | 0.04±0.01 | 0.15±0.04* | 0.08±0.01 | 0.01±0.01 |
| *Gm_GulLO_1e* | 0.01±0 | 0.01±0 | 0.01±0.03 | 0±0.01 | 0.03±0.02 | 0.01±0 | 0.05±0.01 | 0.03±0.04 |
| *Gm_GulLO_1f* | 0.02±0.01 | 0.11±0.18 | 0.05±0.01 | 0.02±0 | 0.45±0.09 | 1.74±0.28* | 2.58±0.02* | 0.23±0.11 |
| *Gm_GulLO_1g* | 0.01±0.01 | 0.04±0.03 | 0.01±0.01 | 0.08±0.03* | 0±0 | 0±0.01 | 0±0 | 0.1±0.17 |
| *Gm_GulLO_3* | 0.16±0.05 | 0.21±0.06 | 0.23±0.06 | 0.14±0.05 | 4.57±0.05 | 2.42±0.29* | 2.86±0.27* | 0.58±0.2* |
| *Gm_GalUR_1* | 0.01±0 | 1.3±1.45 | 0.02±0 | 0.01±0.02 | 15.5±2.12 | 2.24±0.4* | 4.67±0.22* | 9.6±0.6* |
| *Gm_GalUR_2* | 1.3±0.13 | 1.19±0.39 | 1.85±0.32 | 2.13±0.48 | 76.35±7.7 | 104.93±6.54* | 148.03±7.23* | 34.04±8.27* |
| *Gm_GalUR_3* | 0±0 | 0±0 | 0±0 | 0±0 | 0±0 | 0±0 | 0±0 | 0±0 |
| *Gm_GalUR_4* | 0.75±0.08 | 0.86±0.12 | 3.34±0.08* | 0.75±0.12 | 3.65±0.5 | 2.68±0.2 | 3.41±0.38 | 2.77±0.43 |
| *Gm_GalUR_5* | 1.09±0.18 | 0.86±0.04 | 1.17±0.29 | 3.36±0.38* | 81.21±5.11 | 123.8±12.21* | 231.19±1.6* | 145.17±29.62* |
| *Gm_MIOX_1a* | 4.61±1.04 | 2.6±1.22 | 4.69±0.32 | 0.41±0.05* | 9.18±1.07 | 4.42±0.02* | 11.09±0.98 | 4.2±1* |
| *Gm_MIOX_1b* | 4.04±0.82 | 2.22±0.99* | 4.55±0.4 | 0.26±0.04* | 4.37±0.46 | 13.04±0.72* | 27.55±1.62* | 1.82±0.27* |
| *Gm_MIOX_2a* | 0.41±0.13 | 0.67±0.29 | 0.19±0.08 | 10.9±0.98* | 0.81±0.07 | 0.14±0.02* | 0.14±0.05* | 3.56±0.45* |
| *Gm_MIOX_2b* | 0.04±0.03 | 0.07±0.04 | 0.07±0.02 | 0.19±0.03* | 7.46±0.7 | 0.64±0.09* | 2.68±0.52* | 6.67±3.48 |
| *Gm_MIOX_3a* | 0.02±0.01 | 0.02±0.01 | 0.01±0.02 | 0±0 | 0.07±0.01 | 0±0* | 0.03±0.01 | 0.14±0.04* |
| *Gm_MIOX_3b* | 0±0 | 0±0.01 | 0±0 | 0±0.01 | 0.02±0 | 0.02±0.03 | 0.01±0.02 | 0±0 |

| | PRJNA574626 (Submergence) | | | | | | | | | |
|---|---|---|---|---|---|---|---|---|---|---|
| | Leaves | | | | | Roots | | | | |
| | **Control** | **1 D** | **2 D** | **3 D** | **REC** | **Control** | **1 D** | **2 D** | **3 D** | **REC** |
| *Gm_GMP_1a* | 31.72±4.21 | 20.24±1.02* | 14.09±0.68* | 15.36±3.77* | 30.39±0.74 | 78.84±0.41 | 62.36±4.11* | 47.11±6.9* | 21.36±2.41* | 75.36±5.98 |
| *Gm_GMP_1b* | 25.28±1.71 | 7.96±0.54* | 5.37±0.17* | 7.33±0.37* | 60.13±0.82* | 33.44±3.55 | 7.76±0.5* | 5.06±0.34* | 5.44±0.69* | 39.67±4.42 |
| *Gm_GMP_2a* | 2.95±0.26 | 4.72±0.14* | 4.89±0.46* | 5.09±0.45* | 3.94±0.15 | 10.95±0.54 | 4.15±0.49* | 2.64±0.29* | 2.13±0.12* | 8.18±1.28* |
| *Gm_GMP_2b* | 3.43±0.29 | 2.9±0.15* | 2.8±0.09* | 2.78±0.29* | 5.12±0.11* | 11.48±1.24 | 3.15±0.17* | 2.41±0.06* | 1.36±0.08* | 8.57±1.58* |
| *Gm_GMP_alpha_A* | 2.18±0.02 | 0.45±0.01* | 0.3±0.03* | 0.21±0.07* | 2.02±0.03* | 4.36±0.35 | 0.51±0.05* | 0.3±0.05* | 0.26±0.03* | 6.08±0.5* |
| *Gm_GMP_alpha_B* | 4.44±0.87 | 4.07±0.11 | 2.81±0.15* | 2.82±0.08* | 6.69±0.15* | 2.9±0.42 | 0.64±0.14* | 0.44±0.03* | 0.52±0.05* | 3.59±0.33* |
| *Gm_GMP_alpha_C* | 2.2±0.37 | 3.68±0.57* | 4.75±0.81* | 4.38±0.3* | 3.35±0.26 | 5.6±0.93 | 2.74±0.25* | 2.44±0.45* | 1.88±0.27* | 5.97±0.63 |
| *Gm_GGP_1likeA* | 17.13±1.16 | 12.29±1.16* | 9.67±0.45* | 25.35±1.32* | 21.88±0.97* | 11.99±1.01 | 5.71±0.24* | 6.88±0.28* | 5.71±0.28* | 11.65±0.53 |
| *Gm_GGP_1likeB* | 8.2±1.62 | 5.9±0.3 | 4.97±1.05 | 11.54±2.29 | 9.62±0.18 | 4.6±0.52 | 2.09±0.15* | 2.44±0.59* | 3.35±0.28* | 5.38±0.7 |
| *Gm_GGP_1a* | 183.59±13.85 | 81.92±8.98* | 54.28±2.64* | 187.81±16.78 | 302.45±9.25* | 18.23±0.43 | 1.47±0.29* | 1.26±0.11* | 1.26±0.07* | 20.4±0.43* |
| *Gm_GGP_1b* | 136.86±11.65 | 31.76±4.68* | 13.19±1.18* | 79.89±6.63* | 201.48±3.58* | 19.34±1.52 | 0.25±0.05* | 0.13±0.02* | 0.2±0.05* | 8.86±0.44* |
| *Gm_GPP_1* | 19.9±3.03 | 3.08±0.29* | 1.26±0.08* | 2.23±0.06* | 29.69±0.99* | 7±0.49 | 0.88±0.08* | 0.24±0.03* | 0.17±0.04* | 6.34±0.25* |
| *Gm_GPP_2* | 6.2±0.44 | 3.73±0.37* | 1.65±0.17* | 1.79±0.31* | 15.48±1.32* | 2.66±0.38 | 0.35±0.05* | 0.12±0.03* | 0.07±0.03* | 1.46±0.36* |
| *Gm_GPP_L* | 0.19±0.07 | 0.16±0.03 | 0.26±0.05 | 0.92±0.07* | 0.13±0.02 | 0.43±0.05 | 0.04±0.01* | 0±0* | 0±0.01* | 0.07±0.01* |
| *Gm_GalDH_1a* | 3.78±0.3 | 2.06±0.27* | 1.46±0.1* | 1.55±0.13* | 3.29±0.17 | 3.22±0.52 | 0.38±0.07* | 0.12±0.04* | 0.09±0.01* | 3.14±0.7 |
| *Gm_GalDH_1b* | 0.08±0.04 | 0.07±0.03 | 0.08±0.03 | 0.02±0.04 | 0.5±0.08* | 0.15±0.05 | 0±0* | 0±0* | 0±0* | 0.06±0.01* |
| *Gm_GalLDH_1a* | 1.72±0.28 | 2.77±0.3* | 2.54±0.1* | 2.32±0.31 | 3.06±0.13* | 3.69±0.2 | 0.84±0.09* | 0.41±0.09* | 0.39±0.06* | 4.14±0.34 |
| *Gm_GalLDH_1b* | 0.84±0.12 | 4.52±0.42* | 3.91±0.19* | 1.98±0.27* | 1.89±0.06* | 1.84±0.13 | 0.56±0.08* | 0.69±0.1* | 0.6±0.08* | 1.9±0.24 |
| *Gm_GME_1a* | 31.72±6.39 | 6.18±0.25* | 4.92±0.18* | 5.55±0.27* | 8.8±0.19* | 0.31±0.04 | 0.02±0.01* | 0.02±0.01* | 0.01±0.02* | 0.13±0.04* |
| *Gm_GME_1b* | 0.94±0.15 | 6.66±0.82* | 9.33±0.39* | 3.28±0.62* | 0.26±0.05 | 0.06±0.01 | 0.09±0.05 | 0.07±0.01 | 0.04±0 | 0.05±0.02 |

| | | | | | | | | | | |
|---|---|---|---|---|---|---|---|---|---|---|
| *Gm_GME_2a* | 22.78±6.68 | 20.64±2.08 | 20.1±1.15 | 36.28±2.66* | 38.22±1.23* | 14.86±1.99 | 1.67±0.24* | 2.08±0.32* | 1.32±0.14* | 6.18±1.49* |
| *Gm_GME_2b* | 7.44±1.55 | 18.22±0.13* | 20.36±0.82* | 18.11±0.69* | 13.1±0.37* | 9.15±0.85 | 0.32±0.08* | 0.29±0.1* | 0.34±0.06* | 4.6±0.43* |
| *Gm_GulLO_1a* | 0±0 | 0.01±0.01 | 0.03±0 | 0.04±0.04* | 0.01±0 | 14.4±2.99 | 14.04±1.04 | 13.89±0.91 | 9.99±1.19 | 35.77±1.91* |
| *Gm_GulLO_1b* | 0.79±0.15 | 0.58±0.12 | 0.19±0.03* | 0.19±0.05* | 0.96±0.02 | 6.95±0.38 | 5.13±0.57* | 2.13±0.19* | 1.39±0.13* | 9.85±0.84* |
| *Gm_GulLO_1c* | 0.13±0.04 | 0.07±0.01 | 0.05±0.01* | 0.07±0.01 | 0.11±0.04 | 1.79±0.08 | 0.57±0.08* | 0.45±0.08* | 0.2±0.02* | 1.98±0.5 |
| *Gm_GulLO_1d* | 0±0 | 0±0 | 0±0 | 0±0 | 0±0 | 0.09±0.02 | 0.03±0.01* | 0.1±0.01 | 0.09±0.02 | 0.1±0.01 |
| *Gm_GulLO_1e* | 0.05±0.01 | 0.03±0.01 | 0.01±0.03 | 0±0* | 0±0* | 0.01±0 | 0.01±0 | 0.01±0 | 0±0 | 0.01±0 |
| *Gm_GulLO_1f* | 0.01±0 | 0.11±0.02 | 0.18±0.05* | 0.58±0.12* | 0.03±0.02 | 0.39±0.03 | 0.61±0.13* | 0.65±0.02* | 0.54±0.08 | 0.68±0.03 |
| *Gm_GulLO_1g* | 0.05±0.04 | 0.2±0.04* | 0.16±0.06* | 0.17±0.03* | 0.06±0.02 | 0±0.01 | 0±0 | 0±0 | 0±0 | 0±0 |
| *Gm_GulLO_3* | 0.32±0.1 | 0.17±0.02* | 0.12±0.01* | 0.06±0.03* | 0.25±0.02 | 3.06±0.04 | 0.08±0.02* | 0.03±0.01* | 0.01±0.01* | 1.88±0.26* |
| *Gm_GalUR_1* | 0±0 | 0.19±0.02* | 0.35±0.08* | 0.51±0.05* | 0.01±0.02 | 15.53±2.69 | 0.21±0.04* | 0.21±0.03* | 0.17±0.04* | 2.8±0.73* |
| *Gm_GalUR_2* | 0.24±0.11 | 4.89±0.52* | 5.11±0.72* | 14.34±0.47* | 0.43±0.06 | 80.44±6.02 | 0.73±0.09* | 0.25±0.03* | 4.39±0.22* | 17.76±2.39* |
| *Gm_GalUR_3* | 0±0 | 0±0 | 0±0 | 0±0 | 0±0 | 0±0 | 0±0 | 0±0 | 0±0 | 0±0 |
| *Gm_GalUR_4* | 0.35±0.09 | 1.62±0.21* | 1.72±0.1* | 1.51±0.21* | 0.3±0.06 | 4.02±0.22 | 0.98±0.19* | 1.18±0.18* | 1.09±0.28* | 4.05±0.43 |
| *Gm_GalUR_5* | 0.1±0.04 | 1.7±0.3 | 4.03±0.28* | 8.4±1.49* | 0.48±0.07* | 99.7±7.58 | 35.4±1.34* | 17.41±3.71* | 9.44±0.58* | 44.44±8.27* |
| *Gm_MIOX_1a* | 0.02±0.03 | 0.49±0.11* | 0.74±0.11* | 1.36±0.25* | 0.03±0.01 | 5.28±0.72 | 2.48±0.36* | 2.29±0.2* | 2.92±0.44* | 4.06±0.42* |
| *Gm_MIOX_1b* | 0.02±0 | 11.16±1.88* | 36.4±4.11* | 32.45±2.99* | 0.12±0.04 | 6.83±1.24 | 9.37±0.42 | 5.36±0.31 | 5.83±0.13 | 6.89±0.44 |
| *Gm_MIOX_2a* | 3.21±0.25 | 2.01±0.54 | 0.23±0.02* | 1.02±0.32* | 16.33±1.34* | 0.43±0.06 | 0.01±0.02* | 0±0* | 0±0* | 0.07±0.03* |
| *Gm_MIOX_2b* | 0.08±0.02 | 33.46±1.47* | 38.34±1.47* | 16.59±1.21* | 0.12±0.02 | 18.95±2.3 | 5.83±0.62* | 6.99±0.13* | 13.41±0.7* | 5.19±0.57* |
| *Gm_MIOX_3a* | 0.1±0.03 | 0.07±0.01 | 0.4±0.06* | 0.4±0.05* | 0.05±0.01 | 0.09±0.02 | 0.11±0.02 | 1.11±0.15* | 2.39±0.48* | 1.1±0.21* |
| *Gm_MIOX_3b* | 0.01±0.02 | 0±0 | 0±0.01 | 0±0 | 0±0 | 0.02±0.01 | 0±0.01* | 0±0* | 0±0* | 0.01±0 |
