# Supplementary material for: Tissue- and Condition-Specific Biosynthesis of Ascorbic Acid in Glycine max L.: Insights from Genome-Wide Analyses of Pathway-Encoding Genes, Expression Profiling, and Mass Fraction Determination
Source: Int J Mol Sci. 2025 May 14;26(10):4678. doi: 10.3390/ijms26104678 (PMC12111785; doi:10.3390/ijms26104678)
Supplement: Supplementary file 1 [file ijms-26-04678-s001.zip › Suppl. Table S7.pdf]

Supplementary Table S7. Means of RPKM values ± SD (standard deviation) of AsA biosynthesis transcripts in roots of soybean under cyst nematode (SCN) + aphids (SBA) (bioproject PRJNA514200) and *Fusarium virguliforme* (PRJNA549915) as well as in leaves of soybean under SMV (bioproject PRJNA564957), *Phakopsora pachyrhizi* (bioproject PRJDB7011) and spider mite (+/_ insecticides) (bioproject PRJNA515005). Statistical analysis, one-way ANOVA followed by Bonferroni test, was applied with the respective controls of bioproject PRJNA514200, PRJNA564957, and PRJNA515005. While statistical analysis, the t-test, was applied concerning the controls of bioproject PRJNA549915 and PRJDB7011. Up and downregulated genes are in green and red, respectively. Significant differences are indicated by * at $p < 0.05$.

	**PRJNA564957**									
	**L29** (resistant)					**Williams82** (susceptible)				
	**Control**	**2 hpi**	**4 hpi**	**6 hpi**	**8 hpi**	**Control**	**2 hpi**	**4 hpi**	**6 hpi**	**8 hpi**
Gm_GMP_1a	42.01±2.69	46.92±4.58	41.83±4.44	37.32±3.89	45.55±3.81	40.89±4.07	42.53±1.91	38.63±1.56	32.48±2.04	53.19±11.66
Gm_GMP_1b	37.15±2.53	41.71±2.64	33.37±2.24	27.54±2.36*	27.84±1.3*	30.36±2.35	34.48±1.03	32.89±2.9	23.58±0.44*	29.53±0.19
Gm_GMP_2a	7.33±0.19	9.29±0.42*	9.64±0.62*	5.4±0.23*	7.17±0.03	6.57±0.89	7.98±1.13	8.6±1.67	5.76±0.27	7.19±0.8
Gm_GMP_2b	8.2±0.51	8.49±0.64	9.06±0.58	5.12±0.13*	6.36±0.16*	6.68±0.11	7.73±0.91	8.84±1.45	5.6±0.99	6.09±0.46
Gm_GMP_alpha_A	2.39±0.8	2.26±0.59	3.38±0.59	8.44±0.1*	12.14±0.25*	2.21±0.45	1.89±0.06	3.03±0.01	7.17±0.17*	10.66±1.77*
Gm_GMP_alpha_B	4.53±0.8	4.77±0.2	6.02±0.58	9.11±0.16*	12.29±1.43*	4.08±0.17	4.71±0.19	6.16±0.21	7.28±0.53	12.39±1.87*
Gm_GMP_alpha_C	1.63±0.02	1.44±0.2	2.04±0.46	3.29±0.23*	3.61±0.14*	1.21±0.06	1.2±0.16	2.08±0.02*	2.69±0.08*	3.58±0.11*
Gm_GGP_1likeA	46.13±0.06	32.59±1.61*	37.69±2.75*	28.1±0.84*	28.3±0.46*	47.02±1.91	34.11±0.48*	34.18±0.65*	29.85±2.09*	25.88±1.85*
Gm_GGP_1likeB	13.35±0.55	8.21±1.52*	7.88±0.24*	7.58±0.18*	7.48±0.55*	12.52±1.64	9.68±2.24	7.62±0.14	6.11±0.9*	6.87±0.12*
Gm_GGP_1a	166.85±22.17	249.6±25.27	250.61±36.02	123.85±15.59	63.34±4.29*	178.39±16.56	273.14±30.03*	212.43±17.34	98.49±1.39*	53.37±5.17*
Gm_GGP_1b	540.35±80.02	327.51±75.98	182.95±11.89*	126.37±11.4*	73.94±1.29*	600.47±23.05	405.59±24.77*	198.81±19.35*	96.21±11.81*	72.96±5.08*
Gm_GPP_1	30.3±0.62	30.95±3.5	21.03±0.83*	19.97±2.33*	16.3±0.01*	28.41±0.91	33.41±1.62	24.76±1.51	15.98±0.72*	19.07±3.67*
Gm_GPP_2	10.02±0.07	7.81±0.67*	5.04±0.06*	5.19±0.2*	5.12±0.26*	10.75±0.94	8.49±0.46*	5.21±0.25*	4.1±0.7*	4.84±0.19*
Gm_GPP_L	0.08±0.02	0.18±0.14	0.24±0.07	0.1±0.05	0.12±0.06	0.24±0.07	0.21±0.01	0.17±0.01	0.19±0	0.1±0*
Gm_GalDH_1a	11.96±0.14	6.73±0.26*	6.02±0.29*	13.06±0.01*	13.96±0.07*	11.88±0.31	6.4±1*	6.03±0.39*	11.52±0.54	14.71±2.56
Gm_GalDH_1b	0.55±0.26	0.48±0.25	0.79±0.41	1.04±0.12	1.83±0.29*	0.84±0	0.34±0.3	0.71±0.71	0.79±0.11	1.24±0.79
Gm_GalLDH_1a	3.86±0.38	3.64±0.28	2.6±0.2*	4.71±0.19	5.04±0.36*	3.87±0.09	3.42±0.37	2.52±0.14	4.72±0.78	4.52±0.31
Gm_GalLDH_1b	1.38±0.13	0.81±0.1	1.12±0.06	2.68±0.01*	3.33±0.45*	1.44±0.16	0.77±0.04*	1.01±0.01	2.83±0.19*	2.59±0.02*
Gm_GME_1a	78.96±3.62	76.77±8.03	59.49±0.42*	91.22±1.61	88.2±2.84	79.38±0.51	71.49±0.62	62.04±0.6*	76.78±0.58	86.95±7.73
Gm_GME_1b	9.8±2.05	6.38±0.28	4.89±0.03*	4.35±0.74*	5.36±0.43*	9.85±1.03	8.68±1.35	5.42±0.78*	3.97±0.33*	4.69±0.4*
Gm_GME_2a	70.97±3.14	78.95±12.29	45.43±2.11*	36.9±1.07*	38.92±1.32*	73.68±3.43	66.09±6.04	60.93±0.02	36.98±2.17*	42.1±0.77*
Gm_GME_2b	33.77±2.64	31.63±2.36	25.46±0.64*	21.68±0.08*	26.17±0.71*	36.33±0.97	30.02±1.94	26.38±0.85*	22.18±0.49*	27.92±4.45
Gm_GulLO_1a	0±0	0±0	0±0	0±0	0±0	0±0	0±0	0±0	0±0	0±0
Gm_GulLO_1b	0.42±0.12	0.55±0.03	0.85±0.31	1.67±0.06*	1.34±0.19*	0.5±0.08	0.54±0.09	0.71±0.11	2.8±0.49*	1.35±0.41*
Gm_GulLO_1c	0.17±0.04	0.36±0.03	0.31±0.12	0.38±0	0.35±0.11	0.27±0	0.29±0.04	0.27±0.02	0.54±0.16	0.23±0.09
Gm_GulLO_1d	0±0	0±0	0±0	0±0	0±0	0±0	0±0	0±0	0±0	0±0
Gm_GulLO_1e	0±0	0.01±0.01	0.01±0.01	0.02±0	0.01±0.01	0.01±0.02	0.01±0.01	0±0	0.02±0.02	0.02±0.01
Gm_GulLO_1f	0.04±0.04	0.02±0.03	0.02±0.01	0.01±0.02	0±0	0.05±0	0.01±0	0±0	0.02±0	0±0
Gm_GulLO_1g	0.08±0.04	0.03±0.02	0.04±0	0.12±0.01	0.16±0.16	0.03±0.05	0.04±0	0.06±0	0.07±0.08	0.18±0.02
Gm_GulLO_3	1.27±0.04	1.69±0.42	1.38±0.14	1.76±0.22	1.82±0.14	1.75±0.11	1.34±0.05	1.51±0.5	2.79±0.11*	1.56±0.24
Gm_GalUR_1	0±0	0.01±0.01	0.01±0.01	0±0	0.08±0	0±0	0.01±0	0±0	0.02±0.03	0.02±0.03
Gm_GalUR_2	0.49±0.18	0.29±0.08	0.37±0.32	0.28±0.04	0.59±0.19	0.41±0.23	0.42±0.07	0.83±0.25	0.57±0.31	0.91±0.66
Gm_GalUR_3	0.04±0.06	0±0	0±0	0±0	0.01±0.01	0.01±0.01	0.02±0.03	0±0	0.06±0.09	0±0
Gm_GalUR_4	0.4±0.23	0.34±0.02	0.41±0.02	1.17±0.11*	2.08±0.12*	0.45±0.06	0.31±0.02	0.37±0.08	1.3±0.14*	2.13±0.27*
Gm_GalUR_5	0.11±0.07	0.11±0	0.2±0.04	0.52±0.16	0.88±0.34*	0.04±0	0.11±0.02	0.19±0.07	0.46±0.06*	0.56±0.05*
Gm_MIOX_1a	0.07±0.05	0.05±0.03	0.13±0	0.05±0.01	0.54±0.39	0.09±0.05	0.07±0	0.08±0.03	0.59±0.41	0.31±0.18
Gm_MIOX_1b	0.48±0.28	0.23±0.19	0.2±0.07	0.08±0.09	0.99±0.59	0.41±0	0.17±0.03	0.2±0.03	0.69±0.23	0.85±0.82
Gm_MIOX_2a	34.53±2.5	68.58±5.02*	29.9±9.57	9.24±2.87*	10.73±3.77*	26.91±3.36	62.89±7.47*	51.79±4.44*	6.24±0.8*	12.71±5.04
Gm_MIOX_2b	0.25±0.16	2.06±0.53*	0.52±0.08	0.04±0.01	1.04±0.31	0.39±0	0.85±0.16*	1.02±0.23	0.2±0.05	1.32±0.77
Gm_MIOX_3a	0.21±0.07	0.16±0.08	0.05±0.02	0.1±0.02	0.1±0.07	0.14±0.01	0.08±0.02	0.05±0.07	0.19±0.03	0.2±0.13
Gm_MIOX_3b	0±0	0.01±0.01	0±0	0.01±0.02	0.01±0.01	0.01±0	0.02±0.03	0.02±0.02	0.01±0.01	0±0

	PRJDB7011			
	BRS184 (susceptible)		NIL(Rpp3) (resistant)	
	**Mock**	*P. pachyrhizi*	**Mock**	*P. pachyrhizi*
Gm_GMP_1a	7.92±0.55	8.81±0.91	11.06±1.36	7.2±1.8
Gm_GMP_1b	8.25±1.59	6.55±0.96	5.66±0.91	4.36±0.77
Gm_GMP_2a	5.39±0.8	3.66±0.88	2.33±1.14	5.55±1.14*
Gm_GMP_2b	2.93±0.83	1.75±0.36	2.22±0.71	1.54±0.6
Gm_GMP_alpha_A	0.38±0.14	0.83±0.09*	0.69±0.16	2.7±0.59*
Gm_GMP_alpha_B	1.99±0.48	4.7±1*	4.64±0.47	8.04±1.21*
Gm_GMP_alpha_C	2.42±0.65	3.88±0.71	2.3±0.33	3.88±0.5*
Gm_GGP_1likeA	16.92±3.08	22.21±4.36	20.55±4.28	21.38±2.66
Gm_GGP_1likeB	4.78±1.07	6.6±0.7	4.4±0.66	7.29±1.79
Gm_GGP_1a	183.14±7.64	104.66±36.44	187.9±25.76	104.67±8.04*
Gm_GGP_1b	58.07±9.38	34.51±6.92*	63.47±11.01	39.84±5.87*
Gm_GPP_1	2.48±0.56	1.92±0.31	2.91±0.44	1.2±0.21*
Gm_GPP_2	1.41±0.31	1.37±0.12	1.54±0.84	0.94±0.15
Gm_GPP_L	0.28±0.49	0.09±0.03	0.1±0.17	0.18±0.04
Gm_GalDH_1a	2.26±0.29	5.13±0.43*	5±0.87	3.95±0.55
Gm_GalDH_1b	0±0	0±0	0±0	0±0
Gm_GalLDH_1a	3.27±0.48	3.43±0.42	3.36±0.59	2.53±0.94
Gm_GalLDH_1b	2.44±0.4	2.64±0.54	1.96±0.14	1.64±0.37
Gm_GME_1a	4.89±0.88	3.78±0.52	9.36±2.07	8.59±0.95
Gm_GME_1b	2.03±0.25	2.07±0.19	3.09±0.33	1.58±0.24*
Gm_GME_2a	37.57±2.25	32.38±4.03	105.92±10.08	31.77±2.61*
Gm_GME_2b	25.72±1.88	27.96±6.19	35.26±2.75	22.55±3.35*
Gm_GulLO_1a	0.01±0.02	0±0	0.08±0.15	0.02±0.04
Gm_GulLO_1b	0.11±0.07	0.4±0.01*	0.1±0.06	0.93±0.64*
Gm_GulLO_1c	0±0	0±0	0±0	0.07±0.11
Gm_GulLO_1d	0±0	0±0	0±0	0±0
Gm_GulLO_1e	0±0	0.02±0.04	0.09±0.16	0±0
Gm_GulLO_1f	1.99±0.06	2.83±0.47	0.01±0.02	3.01±0.85*
Gm_GulLO_1g	0.01±0.01	0.07±0.07	0.31±0.08	0±0*
Gm_GulLO_3	0.06±0.04	0±0	0.04±0.08	0.19±0.06*
Gm_GalUR_1	1.63±1.31	21.83±4.23*	0.22±0.08	10.31±1.18*
Gm_GalUR_2	47.43±5.21	75.94±4.35*	5.03±0.57	58.97±8.09*
Gm_GalUR_3	0±0	0±0	0±0	0±0
Gm_GalUR_4	1.45±0.31	2.73±0.66	0.59±0.35	1.61±0.68
Gm_GalUR_5	1.81±1.57	44.29±10.52*	0.52±0.26	44.7±19.88*
Gm_MIOX_1a	0.35±0.38	0.1±0.17	0.05±0.03	1.12±1.25
Gm_MIOX_1b	0.21±0.03	0.89±0.18*	0.73±0.22	1.03±0.52
Gm_MIOX_2a	3.63±1.13	1.68±0.16	15.06±4.08	1.96±0.74*
Gm_MIOX_2b	1.35±0.38	2.8±0.69*	5.63±1.28	0.54±0.12*
Gm_MIOX_3a	0±0	0.19±0.33	0±0	0±0
Gm_MIOX_3b	0±0	0±0	0±0	0±0

	PRJNA515005					
	**Control**	**Thiamethoxam**	**Imidacloprid**	**Imidacloprid + Spider mite**	**Thiamethoxam + Spider mite**	**Spider mite**
Gm_GMP_1a	23.94±2.13	21.89±3.14	20.79±4.1	16.28±2.22*	21.56±0.73	21.08±1.86
Gm_GMP_1b	28.78±2.39	26.01±3.87	30.57±5.36	27.2±1.94	33.72±4.05	31.89±4.08
Gm_GMP_2a	3.2±0.36	2.69±0.07	2.93±0.22	2.72±0.6	2.91±0.6	2.51±0.36
Gm_GMP_2b	3.1±0.28	2.55±0.2	2.66±0.14	2.69±0.33	2.67±0.33	2.75±0.42
Gm_GMP_alpha_A	2.48±0.61	2.43±0.2	2.62±0.52	1.47±0.15*	1.45±0.13*	1.35±0.13*
Gm_GMP_alpha_B	2.9±0.86	2.92±0.55	2.99±0.65	2.08±0.33	1.86±0.32	2.07±0.21
Gm_GMP_alpha_C	3.65±0.11	2.77±0.61	2.81±0.64	3.46±0.62	2.89±0.06	2.88±0.5
Gm_GGP_1likeA	28.94±2.25	27.75±0.86	27.47±0.96	26.62±3.09	27.86±0.71	27.73±1.41
Gm_GGP_1likeB	7.11±0.5	5.93±0.05	6.38±1	5.92±0.54	5.76±0.27	5.82±0.38
Gm_GGP_1a	128.82±8.81	119.15±12.48	119.1±4.74	198.43±26.86*	173.68±11.62*	200.19±15.41*
Gm_GGP_1b	71.27±13.93	62.14±9.26	64.46±8.36	89.84±6.14	82.89±3.7	84.76±19.46
Gm_GPP_1	11.05±1.25	10.54±2.05	12.44±1.79	13.01±2.9	14.78±1.75	14.19±2.18
Gm_GPP_2	2.99±0.29	1.74±0.07*	2.4±0.34	3.14±0.96	3.37±0.31	3.78±0.48
Gm_GPP_L	0.22±0.04	0.28±0.05	0.26±0.02	0.27±0.03	0.2±0.03	0.3±0.05
Gm_GalDH_1a	5.56±1.24	5.69±0.58	6.22±0.33	5.3±0.87	5.72±0.37	5.14±0.19
Gm_GalDH_1b	0.13±0.03	0.15±0.04	0±0*	0.12±0.03	0.05±0.09	0.21±0.06
Gm_GalLDH_1a	3.27±0.73	2.64±0.17	2.96±0.31	3.21±0.36	3±0.24	2.71±0.52
Gm_GalLDH_1b	2.34±0.48	1.81±0.17	1.7±0.45	1.69±0.21	1.84±0.28	1.49±0.27*
Gm_GME_1a	33.67±4.35	32.5±0.85	44.25±6.63	41.48±4.78	47.86±3.33*	47.4±5.18*
Gm_GME_1b	3.11±0.41	5.05±1.2*	2.56±0.07	2.45±0.64	4.52±0.36	3.55±0.29
Gm_GME_2a	43.2±5.38	32.15±2.91	38.54±7.46	54.57±10.2	49.94±6.39	47.44±5.36
Gm_GME_2b	37.59±4.43	31.62±1.04	37.33±3.43	33.48±0.78	38.68±1.71	37.82±2.41
Gm_GulLO_1a	0±0	0±0	0±0	0±0	0±0	0±0
Gm_GulLO_1b	0.32±0.11	0.34±0.11	0.37±0.15	0.4±0.04	0.43±0.16	0.46±0.08
Gm_GulLO_1c	0.07±0.03	0.16±0.04*	0.07±0.01	0.15±0.02*	0.09±0.02	0.17±0.03*
Gm_GulLO_1d	0±0	0±0	0±0	0±0	0±0	0±0
Gm_GulLO_1e	0±0	0.01±0.01	0.01±0.01	0±0	0.01±0.02	0±0
Gm_GulLO_1f	0.12±0.03	0.06±0.01	0.03±0.01	0.04±0.01	0.08±0.02	0.03±0.05
Gm_GulLO_1g	0.25±0.04	0.23±0.07	0.09±0.03*	0.23±0.08	0.08±0.06*	0.22±0.03
Gm_GulLO_3	0.43±0.06	0.46±0.04	0.18±0.04*	0.43±0.04	0.42±0.03	0.48±0.06
Gm_GalUR_1	0.14±0.05	0.05±0.01	0.07±0.01	0.04±0.01*	0.08±0.04	0.1±0.04
Gm_GalUR_2	6.05±1.15	2.87±0.48*	1.54±0.1*	2.78±0.66*	2.77±0.33*	3.42±0.49*
Gm_GalUR_3	0±0	0±0	0±0	0±0	0±0	0±0
Gm_GalUR_4	1.54±0.14	1.23±0.22	1.37±0.23	0.99±0.21*	0.68±0.03*	0.78±0.18*
Gm_GalUR_5	3.3±0.43	0.99±0.17*	0.95±0.32*	0.83±0.08*	0.7±0.08*	1.17±0.17*
Gm_MIOX_1a	0.13±0.02	0.03±0.01	0.07±0.01	0.03±0.01	0.02±0.03	0.16±0.11
Gm_MIOX_1b	0.71±0.09	0.45±0.13*	0.43±0.08*	0.45±0.02*	0.23±0.1*	0.62±0.14
Gm_MIOX_2a	10.13±1.13	8.42±1.92	10.23±0.98	9.28±0.79	8.16±0.7	9.78±1.69
Gm_MIOX_2b	1±0.16	1.08±0.24	0.61±0.1	6.46±1.18*	4.05±1.49*	2.22±0.11*
Gm_MIOX_3a	0.01±0.03	0.01±0.02	0±0	0.01±0.02	0.04±0.01	0±0
Gm_MIOX_3b	0.01±0.02	0±0	0.01±0.02	0±0	0±0	0±0

PRJNA514200								
**PI518671** (SCN and SBA susceptible)								
	**5 days**				**30 days**			
	Control	Aphid	SCN	SCNAphid	Control	Aphid	SCN	SCNAphid
Gm_GMP_1a	57.91±4.18	87.35±7.13*	65.7±12.87	93.46±3.54*	71.88±10.7	69.12±5.29	75.78±5.05	79.55±34.31
Gm_GMP_1b	19.68±5	56.97±3.23*	42.97±4.69*	45.37±9.51*	62.55±5.91	60.51±22.45	53.91±3.87	44.1±7.1
Gm_GMP_2a	14±3.19	14.94±2.7	8.69±1.67	14.21±1.76	14.2±2.55	11.36±1.24	13.01±2.06	13.59±3.11
Gm_GMP_2b	8.41±1.86	15.81±2.86*	9.93±1.38	13.44±0.22*	14.54±1.39	14.62±1.32	11.24±1.74	11.91±1.34

Gm_GMP_alpha_A	1.47±0.35	5.34±1.05*	4.14±0.54*	4.39±0.76*	5.33±0.92	2.98±0.68	5.02±0.65	4.89±1.55
Gm_GMP_alpha_B	0.89±0.36	4.47±0.95*	3.79±0.41*	4.1±0.99*	4.82±0.5	3.1±0.3*	5.33±0.56	7.66±1.13*
Gm_GMP_alpha_C	10.8±0.64	12.53±0.79	11.22±1.24	11.99±1.15	11.2±0.41	10.56±1.16	9.94±0.21	9.97±1.77
Gm_GGP_1likeA	38.4±2.66	38.32±3	23.21±3.1*	28±3.65*	22.49±1.87	22.92±4.47	23±1.89	22.89±3.19
Gm_GGP_1likeB	12±1.48	13.1±2.81	13.8±0.41	13.46±1.78	10.64±1.33	11.56±2.22	12.79±1.4	13.24±1.84
Gm_GGP_1a	6.47±2.21	38.5±4.31*	57.43±5.9*	68.28±4.26*	39.98±6.26	51.63±6.52	61.78±2.24*	66.4±15.02*
Gm_GGP_1b	13.32±0.78	135.93±9.23*	230.99±39.04*	242.59±14.05*	125.99±23.72	146.98±17.99	148.11±15.07	135.36±13.52
Gm_GPP_1	7.17±1.04	29.25±2.85*	27.12±6.25*	22.01±3.51*	19.96±1.88	16.12±3.99	20.06±1.91	13.97±3.77
Gm_GPP_2	6.33±2.14	21.75±2.93*	19.07±3.39*	16.9±3.84*	14.24±1.09	8.83±1.55*	12.32±1.43	6.27±1.33*
Gm_GPP_L	1.45±1.14	28.68±11.47*	28.17±4.14*	23.15±3.17*	17.97±3.9	20.34±5.34	23.06±7.25	9.31±1.47
Gm_GalDH_1a	2.2±1.05	11.92±3.15*	10.64±2.68*	10.02±3.61*	11.99±2.47	9.38±2.53	13.35±1.26	17.07±4.26
Gm_GalDH_1b	0.05±0.09	0.14±0.03	0.05±0.09	0.09±0.05	0.36±0.08	0.17±0.07	0.29±0.14	0.27±0.18
Gm_GalLDH_1a	11.97±3.48	13.42±1.17	8.2±1.62	9.1±0.54	8.85±1.18	5.61±0.35*	7.32±0.56	7.53±1.39
Gm_GalLDH_1b	3.48±0.49	4.97±0.22*	3.55±0.29	3.94±0.24	4.03±0.15	2.96±0.42	3.51±0.68	4.12±0.33
Gm_GME_1a	0.56±0.3	2.17±0.5	10.52±2.83*	10.18±1.34*	14.07±1.84	10.81±3.66	25.19±4.28*	55.69±4.71*
Gm_GME_1b	0.07±0.05	0.19±0.04	0.15±0.08	0.16±0.07	0.33±0.11	0.38±0.04	0.2±0.06	0.28±0.06
Gm_GME_2a	13.88±2.19	48.13±7.32*	69.31±4.82*	69.14±8.11*	51.27±4.99	70.48±10.15	66±9.89	88.17±9.17*
Gm_GME_2b	7.47±0.97	67.5±6.47*	60.53±4.37*	65.83±10.28*	45.07±3.81	41.09±2.18	41.9±8.04	49.94±6.79
Gm_GulLO_1a	7.54±2.26	19.13±5.21	22.18±6.2	47.32±9.96*	45.61±13.11	50.95±13.31	20.88±2.64*	24.29±3.5
Gm_GulLO_1b	6.53±2.49	10.94±1.47	12.33±5.28	10.1±2.33	14.22±1.56	7.35±1.25*	11.24±2.63	9.37±2.31
Gm_GulLO_1c	0.81±0.21	2.33±0.09*	0.47±0.18	0.81±0.3	1.49±0.11	0.65±0.07*	1.34±0.15	1.25±0.22
Gm_GulLO_1d	0.09±0.06	0.05±0.08	0.05±0	0.01±0.01	0.01±0.01	0.05±0.04	0.1±0.01*	0.1±0.04*
Gm_GulLO_1e	0±0	0.02±0.03	0.3±0.22*	0.11±0.08	0.08±0.01	0.13±0.05	0.38±0.09	0.88±0.53*
Gm_GulLO_1f	6.34±1.96	5.36±0.35	4.17±0.53	4.34±0.48	3.17±0.9	2.26±0.31	3.95±1.47	3.95±0.94
Gm_GulLO_1g	0.02±0.03	0±0	0.03±0.01	0±0	0.06±0.02	0.06±0.05	0.08±0.02	0.06±0.02
Gm_GulLO_3	0.37±0.07	10.57±0.89*	5.07±0.83*	9.72±0.56*	8.56±0.5	4.47±0.55*	6.16±0.97*	5.02±1.11*
Gm_GalUR_1	8.03±3.23	85.43±11.09*	55.72±20.18*	44.96±9.7*	156.52±7.48	56.74±14.86*	91.04±7.62*	63.6±5.44*
Gm_GalUR_2	31.22±13.87	321.04±111.73*	388.71±42.61*	261.41±13.3*	514.02±26.88	312.9±45.14*	391.28±68.03*	303.66±26.78*
Gm_GalUR_3	0±0	0.02±0.03	0±0	0.03±0.02	0.53±0.08	0.15±0.05*	0.03±0.01*	0±0*
Gm_GalUR_4	15.03±4.13	17.63±3.92	17.2±0.54	17.99±3.1	19.47±0.63	14.07±1.66*	15.11±0.91*	15.49±2.48*
Gm_GalUR_5	143.65±76.05	661.3±118.75*	218.32±26.55	256.17±5.91	517.5±29.54	403.49±56.86*	358.61±40.01*	237.39±25.35*
Gm_MIOX_1a	1.61±0.56	1.77±0.3	13.05±5.24*	6.63±1.1	3.19±0.84	4.29±0.45	11.78±2.66*	19.65±1.67*
Gm_MIOX_1b	4.18±1.33	4.85±0.43	10.38±1.46*	7.3±1.67	3.6±0.3	2.98±0.49	4.69±0.94	9.45±2.13*
Gm_MIOX_2a	0.24±0.07	2.72±0.2*	1.04±0.25*	0.81±0.17*	0.59±0.09	0.26±0.05*	0.25±0.02*	0.3±0.11*
Gm_MIOX_2b	8.2±1.59	8.37±1.83	40.61±9.35*	19.08±3.01	16.49±1.95	25.37±6.8	24.99±6.48	42.91±6.7*
Gm_MIOX_3a	0.53±0.17	1.1±0.22*	1.74±0.23*	2.14±0.24*	1.27±0.42	0.71±0.06	1.77±0.34	7.48±2.32*
Gm_MIOX_3b	0.01±0.02	0±0	0.05±0.08	0.03±0.02	0.01±0.01	0.01±0.01	0±0	0.04±0.02

PRJNA 514200								
**MN1806CN** (SCN resistant and SBA susceptible)								
	**5 days**				**30 days**			
	Control	Aphid	SCN	SCNAphid	Control	Aphid	SCN	SCNAphid
Gm_GMP_1a	36.55±2.53	47.29±12.18	52.91±4.36	52.33±8.03	33.87±10.87	24.51±2.29	28.64±5.49	23.03±1.47
Gm_GMP_1b	29.74±4.57	36.79±5.02	35.26±2.59	42.62±10.64	40.04±10.09	25.06±2.95*	28.3±3.81*	19.97±3.51*
Gm_GMP_2a	14.53±2.62	15.62±2.07	15.45±0.63	13.85±2.72	9.3±1.53	8.32±1.07	10.44±0.39	11.11±1.19
Gm_GMP_2b	11.01±0.34	14.32±2.71	11.54±0.76	11.74±2.56	10.04±1.99	6.64±0.17	8.66±0.8	8.52±0.56
Gm_GMP_alpha_A	2.2±0.35	2.8±0.32	2.52±0.5	3.72±0.63*	2.12±0.54	2.02±0.42	1.72±0.37	2.42±0.39
Gm_GMP_alpha_B	1.31±0.16	0.78±0.19	1.74±0.33	2.85±0.54*	1.78±0.14	1.83±0.52	1.47±0.18	2.03±0.38
Gm_GMP_alpha_C	12.35±2.31	13.78±1.07	16.51±1.08*	10.91±0.71	9.94±0.16	9.93±1.38	9.08±0.69	10.22±0.81
Gm_GGP_1likeA	39.31±6.24	48.93±4.39	49.32±6.77	29.25±4.44	21.5±2.91	20.9±2.66	19.08±1.43	27.06±2*
Gm_GGP_1likeB	14.94±1.62	15.52±3.04	19.16±2	10.8±0.4	10.22±1.03	10.47±0.91	10.68±1.47	11.78±0.16
Gm_GGP_1a	39.83±5.46	30.14±2.89	68.34±5.65*	33.55±1.11	32.72±9.23	21.76±2*	30.4±4.01	33.64±3.11
Gm_GGP_1b	113.34±7.97	131.44±9.05	135.65±46.4	151.34±28.87	151.56±51.8	107.83±19.3	156.91±23.4	100.56±13.08
Gm_GPP_1	6.14±1.94	11.41±1.48	8.06±0.73	20.56±4.37*	14.47±6.28	14.59±3.85	14.64±2.17	7.36±2.66
Gm_GPP_2	6.3±0.85	8.82±1.65	6.06±0.67	9.43±1.86	7.42±1.06	6.95±0.6	7.01±1.19	3.98±0.77*
Gm_GPP_L	10.37±4.08	10.67±1.7	4.26±0.57*	5.86±0.88	31.9±11.99	33.88±3.83	17.17±3.18*	4.54±2.13*
Gm_GalDH_1a	4.39±1.32	4.61±0.89	6.5±0.75	9.79±2.24*	6.84±0.42	7.49±0.72	7.73±0.93	5.79±1.12
Gm_GalDH_1b	0±0	0.06±0.1	0.09±0.15	0.1±0.17	0.05±0.07	0±0	0±0	0.06±0.1
Gm_GalLDH_1a	7.17±1.32	8.9±3.6	8.67±0.77	8.65±0.82	8.24±2.2	7.4±0.76	6.64±0.72	8.65±0.55
Gm_GalLDH_1b	5.26±0.91	6.92±0.85	7.7±0.98	4.72±1.27	2.99±0.06	2.82±0.62	3.07±0.36	4.42±0.56
Gm_GME_1a	6.34±2.48	1.33±0.46*	5.91±1.71	9.37±2.19	10.12±7.2	1.29±0.1*	6.98±2.21	5.04±0.93
Gm_GME_1b	0.28±0.06	0.4±0.09	1.14±0.14*	0.42±0.07	0.27±0.14	0.2±0.05	0.08±0.02*	0.25±0.07
Gm_GME_2a	55.31±7.15	37.65±4.75	52.93±6.26	34.38±4.01	33.44±11.52	22.72±3.4	43.44±1.04	28.53±4.69
Gm_GME_2b	50.41±7.36	47.93±5.85	42.72±2.42	38.38±3.08	43.33±11.89	37.07±1.95	50.93±5.67	29.72±8.65
Gm_GulLO_1a	37.61±7.49	75.84±7.92*	81.34±15.89*	92.61±12.68*	45.9±11.06	77.88±9.48*	58.03±7.85	67.18±7.27*
Gm_GulLO_1b	3.75±1.06	7.76±1.36*	6.08±1.26	13.12±2.29*	11.43±1.44	17.1±2.72*	9.69±1.92	10.59±2.26
Gm_GulLO_1c	0.32±0.07	0.55±0.31	0.61±0.14	1.02±0.21*	1.37±0.88	1.47±0.16	0.54±0.04*	0.3±0.05*
Gm_GulLO_1d	0.03±0.02	0.04±0.02	0.02±0.01	0.04±0.02	0.08±0	0.34±0.18*	0.14±0.02	0.08±0.04
Gm_GulLO_1e	0.01±0.02	0.03±0.01	0.06±0.02	0.17±0.03	0.02±0.03	0.03±0.01	0.14±0.01	0.11±0.07
Gm_GulLO_1f	8.15±1.95	14.89±1.51*	17.16±3.38*	8.67±2.6	3.93±0.91	7.09±1.94*	6.6±1.21	6.42±0.34
Gm_GulLO_1g	0.01±0.02	0±0	0±0	0±0	0.01±0.01	0.02±0.03	0.01±0.01	0.01±0.02
Gm_GulLO_3	3.02±0.67	9.34±2.95*	5.59±1.02	7.2±0.62*	4.56±0.93	3.87±0.7	3.26±0.73	2.68±0.52
Gm_GalUR_1	112.88±20.09	105.15±6.85	61.9±10.76*	127.29±2.92	79.81±34.99	48.54±7.85	82.73±13.75	48.65±9.23
Gm_GalUR_2	196.62±19.7	223.39±7.38	255.87±44.86	216.98±22.79	364.26±35.67	365.96±57.11	367.43±49.85	156.9±16.33*

Gm_GalUR_3	0.1±0.18	0.12±0.08	0±0	0.08±0.13	0.21±0.1	0.05±0.01	0.54±0.16*	0.1±0.06
Gm_GalUR_4	20.37±1.74	23.02±1.31	21.66±1.63	18.91±1.93	22.84±0.29	24.19±1.78	23.17±1.64	20.68±1.84
Gm_GalUR_5	352.35±60.75	338.03±19.67	234.59±61.18*	367.92±35.9	285.17±11.24	213.85±23.81*	258.11±29.28	190.89±13.68*
Gm_MIOX_1a	11.72±2.8	14.5±1.49	30.52±6.94*	8.73±3.55	5.02±0.83	20.53±3*	5.48±0.92	4.53±1.01
Gm_MIOX_1b	21.99±4.12	19.59±4.67	31.38±9.04	8.92±1.24	4.27±0.92	8.51±0.84*	11.09±1.69*	4.84±1.03
Gm_MIOX_2a	1.34±0.18	1±0.18	0.41±0.1*	1.67±0.07	2.57±2.45	0.62±0.15	0.44±0.11	0.15±0.03*
Gm_MIOX_2b	31.02±8.67	40.09±11.62	62.04±17.08*	10.35±2.65	8.17±1.42	23.47±5.13*	19.79±3.27*	4.73±1.16
Gm_MIOX_3a	16.35±4.28	24.35±3.29	66.87±11.14*	1.8±0.31	1.78±0.9	11.23±6.86*	6.04±0.34	2.89±0.4
Gm_MIOX_3b	0±0	0.02±0.03	0.01±0.02	0.01±0.02	0.02±0.02	0±0	0±0	0±0

PRJNA 549915												
	0 day		2 days		4 days		7 days		10 days		14 days	
	**Mock**	**Fv**	**Mock**	**Fv**	**Mock**	**Fv**	**Mock**	**Fv**	**Mock**	**Fv**	**Mock**	**Fv**
Gm_GMP_1a	108.77±8.71	122.36±7.31	59.1±6.77	76.54±7.9	58.66±4.62	54.92±10.13	57.33±3.53	38.58±5.13*	75.53±10.16	45.94±4.82*	55.3±6.04	25.15±4.52*
Gm_GMP_1b	78.25±2.12	81.15±9.26	61.67±3.72	60.09±5.64	44.51±1.21	37.21±4.46	37.24±6.01	26.36±5.06	35.43±4.03	25.02±3.63*	20.61±3.1	15.71±1.56
Gm_GMP_2a	28.68±0.57	33.55±4.07	23.3±3.49	24.85±3.89	18.13±2.7	16.23±2.15	14.56±2.72	12.72±2.24	13.78±1.96	9.84±1.73	13.9±2.23	8.03±1.28*
Gm_GMP_2b	20.69±1.23	17.75±5.31	15.39±0.77	16.61±2.12	14.14±2.04	13.11±1.95	11.62±2.21	8.64±1.38	9.97±1.1	7.22±1.14	10.71±2.35	4.55±0.65*
Gm_GMP_alpha_A	17.44±0.99	11.38±1.58*	11.59±1.09	10.37±1.62	9.75±1.26	7.84±0.64	10.46±1.29	8.06±1.16	9.65±0.7	6.6±1.03	7.63±1.31	3.89±0.44*
Gm_GMP_alpha_B	25.01±2.65	18.9±4.51	19.03±1.79	12.63±1.49*	14±3.38	9.26±0.69	8.86±0.94	9.15±1.21	7.53±1.13	7.54±0.6	4.81±0.55	4.91±0.53
Gm_GMP_alpha_C	10.89±1.53	9.86±1	10.62±0.81	9.05±1.24	9.8±0.55	8.49±1.19	11.05±1.04	12.42±1.23	10.01±0.93	11.83±0.93	10.76±1.87	11.57±0.61
Gm_GGP_1likeA	50.14±1.63	44.17±2.29	43.28±0.28	39.94±1.7	39.91±0.78	38.65±2.53	39.66±2.9	35.85±1.57	36.22±2.51	39.7±3.59	42.28±0.3	39.27±3.69
Gm_GGP_1likeB	8.3±1.05	9.84±0.75	5.15±0.43	6.27±0.62	6.14±0.7	7.06±0.71	7.9±0.72	7.15±0.88	10.86±1.63	10.17±1.45	10.51±1.28	10.08±1.65
Gm_GGP_1a	150.24±0.39	86.56±15.91*	41.78±2.94	32.51±4.53	35.98±2.9	35.48±0.66	34.44±1.34	24.55±1.95*	27.63±4.81	30.51±2.84	33.46±1.73	29.23±7.93
Gm_GGP_1b	87.44±7.36	71.36±5.79	19.74±3.63	9.63±2.08*	10.15±0.74	14.63±2.47	9.06±1.1	10.27±1.32	5.75±1.25	22.46±2.69*	9.8±1.05	21.93±3.27*
Gm_GPP_1	21.39±1.37	12.93±1.25*	14.56±1.36	12.3±3.37	12.47±0.83	10.27±2.13	12.62±2.6	7.52±1.29	7.61±1.05	4.53±0.81*	6.71±1.01	4.6±0.33
Gm_GPP_2	6.25±0.54	3.47±0.78*	2.98±0.39	2.21±0.33	3.66±0.69	3.16±0.87	3.16±0.57	1.76±0.48*	2.4±0.39	1.33±0.22*	2.42±0.43	0.8±0.1*
Gm_GPP_L	1.24±0.21	1.07±0.31	0.31±0.07	0.89±0.2*	1.27±0.41	0.57±0.11	0.71±0.18	1.08±0.14	0.69±0.05	1.81±0.23*	0.34±0.07	0.99±0.12*
Gm_GalDH_1a	19.8±1.54	13.03±3.25	11.34±1.56	11.05±1.51	11.93±2.47	8.74±0.64	8.44±2.4	7.91±1.09	9.2±2.12	7.42±0.41	5.39±0.84	4.79±0.58
Gm_GalDH_1b	0±0	0±0	0.36±0.63	0.7±1.21	0±0	0.02±0.03	0±0	0.21±0.19*	0.13±0.22	0.01±0.02*	0.3±0.18	0.11±0.03
Gm_GalLDH_1a	7.93±0.51	6.89±1.31	6.62±1.19	7.84±1.04	7.09±1.02	5.64±0.19	6.79±0.87	5.61±1.4	7.53±0.78	5.43±1.21	9.12±0.92	5.19±0.96*
Gm_GalLDH_1b	5.77±0.25	4.9±0.54	6.77±0.8	4.02±0.73*	3.2±0.41	3.44±0.49	4.59±0.58	3.79±0.49	5.66±1.21	3.38±0.94	8.39±1.59	4.07±0.92*
Gm_GME_1a	12.45±3.67	3.78±1.16	2.77±0.95	1.2±0.59	0.63±0.45	0.51±0.16	0.31±0.08	0.63±0.2	0.42±0.18	3.86±0.81*	1.04±0.23	1±0.48
Gm_GME_1b	2.98±0.68	1.24±0.09*	1.24±0.11	1.56±0.22	0.82±0.15	1±0.19	0.81±0.05	0.84±0.24	0.64±0.1	1.21±0.21	0.61±0.06	0.98±0.42
Gm_GME_2a	19.25±4.05	15.7±2.98	15.65±1.96	16.78±4.07	19.9±3.53	21.33±4.75	26±4.47	25.01±3.66	34.92±3.56	32.3±1.01	53.57±2.48	34.67±2.05*
Gm_GME_2b	19.16±1.31	19.09±2.66	20.53±5.64	19.53±1.12	23.11±4.87	25.52±1.08	27.31±2.68	24.74±6.71	44.51±1.65	26.74±5.38*	72.01±5.68	27.31±2.9*
Gm_GulLO_1a	7.57±0.67	6.72±0.55	1.13±0.65	0.53±0.14	1.69±0.8	4.21±0.63*	3.22±0.61	32.15±6.08*	7.03±1.37	79.04±2.63*	5.98±2.37	67.63±10.88*
Gm_GulLO_1b	10.65±2.68	11.68±1.86	3.71±0.99	4.7±1.4	3.87±0.38	3.31±0.91	3.34±0.48	4.65±0.79	2.37±0.41	9.07±1.92*	1.14±0.32	5.8±1.58*

Gm_GulLO_1c	10.61±0.99	13.73±2.48	13.21±7.22	12.36±2.02	10.36±2.57	8.62±1.08	10.97±3.61	5.34±0.79*	7.48±0.91	4.16±0.32*	5.63±1.21	1.47±0.52*
Gm_GulLO_1d	0.03±0.01	0±0	0±0	0±0	0±0	0±0	0±0	0±0	0±0	0.02±0.01	0±0	0.02±0.02
Gm_GulLO_1e	0.04±0.01	0±0*	0±0	0±0	0±0	0.01±0.01	0.02±0.01	0.01±0.01	0.01±0.01	0±0	0.08±0.03	0.03±0.02
Gm_GulLO_1f	0.85±0.21	2.02±0.31*	1.35±0.42	5.2±0.5*	3.24±0.9	15.25±3.4*	4.3±0.89	25.33±7.67*	4.59±0.8	74.41±7.16*	3.21±0.87	92.8±18.97*
Gm_GulLO_1g	0±0	0±0	0±0	0±0	0±0	0.01±0.01	0±0	0±0	0±0	0.06±0.1*	0.01±0.01	0.01±0
Gm_GulLO_3	27.1±1.72	26.63±5.03	40.57±4.47	47.15±11.01	26.75±3.81	26.07±5.34	29.79±4.91	13.35±2.16*	26.87±3.73	7.51±0.55*	18.96±2.65	3.17±2.19*
Gm_GalUR_1	36.72±6.78	73.24±13.36	38.18±4	106.78±9.09*	44.17±8.14	155.59±22.81*	37.26±10.82	232.49±34.02*	18.15±2.27	366.82±27.96*	13.22±5.55	540.42±34.33*
Gm_GalUR_2	127.55±32.21	316.03±66.79*	150.19±30.09	298.51±20.64*	207.36±28.52	474.71±56.19*	154.02±15.12	799.92±80.89*	90.15±6.28	903.3±99.21*	72.94±32.55	1329.09±109.87*
Gm_GalUR_3	0.05±0.08	0±0*	0±0	0±0	0±0	0±0	0±0	0±0	0±0	0±0	0±0	0±0
Gm_GalUR_4	7.67±1.16	7.72±0.63	5.16±0.81	7.89±2.05	6.72±0.68	8.19±1.19	10.1±1.81	10.36±1.91	12.05±1.48	11.89±1.18	15.06±1.61	16.42±2.38
Gm_GalUR_5	126.28±9.93	208.93±30.1*	128.3±26.37	230.7±42.25*	176.6±21.68	327.93±45.74*	152.24±9.67	465.58±60.91*	118.43±9.73	812.11±124.58*	126.53±22.23	810.17±127.82*
Gm_MIOX_1a	15.4±4.18	6.49±1.7	3.23±0.96	3.82±1.47	2.89±0.7	3.4±0.45	3.24±0.61	5.78±1.16*	3.79±1.11	11.92±2.93*	14.66±0.49	11.37±4.36
Gm_MIOX_1b	44.59±4.15	25.84±3.53*	13.84±7.77	9.71±4.45	10.98±3.16	11.19±1.51	8.77±1.04	10.22±0.88	6.32±1.26	12.16±1.98*	10.97±2.95	15.45±2.7
Gm_MIOX_2a	0±0	0.07±0.08*	0±0	0.31±0.07*	0.1±0.02	0.11±0.04	0.13±0.13	0.23±0.12	0.02±0.03	0±0	0.03±0.01	0±0
Gm_MIOX_2b	119.61±7.39	130.68±57.56	63.14±18.89	96.23±7.68	66.87±15.66	104.95±31.14	76.15±8.65	150.23±6.98*	79.04±9.38	229.01±20.18*	149.88±9.66	218.94±27.9*
Gm_MIOX_3a	0.34±0.09	0.58±0.59	0.52±0.48	0.33±0.14	0.06±0.1	0.79±0.23*	0.45±0.16	48.25±19.31*	4.22±1.43	148.47±25.25*	1.96±0.57	210.73±11.41*
Gm_MIOX_3b	0±0	0±0	0.06±0.1	0.05±0.03	0.05±0.09	0±0.01	0±0	0±0	0±0	0.14±0.1	0±0.01	0±0.01
